# Effects of Dairy Intake on Markers of Cardiometabolic Health in Adults: A Systematic Review with Network Meta-Analysis

**DOI:** 10.1016/j.advnut.2023.03.004

**Published:** 2023-03-11

**Authors:** Eva Kiesswetter, Julia Stadelmaier, Maria Petropoulou, Jakub Morze, Kathrin Grummich, Isabelle Roux, Roberta Lay, Lisa Himmelsbach, Martin Kussmann, Christine Roeger, Malte Rubach, Hans Hauner, Lukas Schwingshackl

**Affiliations:** 1 Institute for Evidence in Medicine, Medical Center & Faculty of Medicine, University of Freiburg, Freiburg, Germany; 2 Institute of Medical Biometry and Statistics, Medical Center & Faculty of Medicine, University of Freiburg, Freiburg, Germany; 3 Department of Epidemiology and Health Promotion, School of Public Health, Center of Postgraduate Medical Education, Warsaw, Poland; 4 Department of Nutrition, Harvard T.H. Chan School of Public Health, Boston, MA, United States; 5 Cochrane Germany, Cochrane Germany Foundation, Freiburg, Germany; 6 Competence Center for Nutrition, Bavarian State Ministry for Food, Agriculture and Forestry, Freising, Germany; 7 Else Kröner-Fresenius-Center for Nutritional Medicine, ZIEL–Institute for Food and Health, Technical University of Munich, Freising, Germany; 8 Institute of Nutritional Medicine, School of Medicine, Technical University of Munich, Munich, Germany

**Keywords:** dairy products, energy intake, body weights and measures, glycemic control, cardiometabolic risk, systematic review, network meta-analysis

## Abstract

The health effects of dairy products are still a matter of scientific debate owing to inconsistent findings across trials. Therefore, this systematic review and network meta-analysis (NMA) aimed to compare the effects of different dairy products on markers of cardiometabolic health. A systematic search was conducted in 3 electronic databases [MEDLINE, Cochrane Central Register of Controlled Trials (CENTRAL), and Web of Science; search date: 23 September 2022]. This study included randomized controlled trials (RCTs) with a ≥12-wk intervention comparing any 2 of the eligible interventions [e.g., high dairy (≥3 servings/d or equal amount in grams per day), full-fat dairy, low-fat dairy, naturally fermented milk products, and low dairy/control (0–2 servings/d or usual diet)]. A pairwise meta-analysis and NMA using random-effects model was performed in the frequentist framework for 10 outcomes [body weight, BMI, fat mass, waist circumference, low-density lipoprotein cholesterol, high-density lipoprotein (HDL) cholesterol, triglycerides, fasting glucose, glycated hemoglobin, and systolic blood pressure]. Continuous outcome data were pooled using mean differences (MDs) and dairy interventions ranked using the surface under the cumulative ranking curve. Nineteen RCTs with 1427 participants were included. High-dairy intake (irrespective of fat content) showed no detrimental effects on anthropometric outcomes, blood lipids, and blood pressure. Both low-fat and full-fat dairy improved systolic blood pressure (MD: −5.22 to −7.60 mm Hg; low certainty) but, concomitantly, may impair glycemic control (fasting glucose—MD: 0.31–0.43 mmol/L; glycated hemoglobin—MD: 0.37%–0.47%). Full-fat dairy may increase HDL cholesterol compared with a control diet (MD: 0.26 mmol/L; 95% CI: 0.03, 0.49 mmol/L). Yogurt improved waist circumference (MD: −3.47 cm; 95% CI: −6.92, −0.02 cm; low certainty), triglycerides (MD: −0.38 mmol/L; 95% CI: −0.73, −0.03 mmol/L; low certainty), and HDL cholesterol (MD: 0.19 mmol/L; 95% CI: 0.00, 0.38 mmol/L) compared with milk. In conclusion, our findings indicate that there is little robust evidence that a higher dairy intake has detrimental effects on markers of cardiometabolic health.

This review was registered at PROSPERO as CRD42022303198.

**Statement of Significance**

This network meta-analysis evaluated the effects of different dairy products and different fat contents of dairy on anthropometric outcomes, blood lipids, glycemic control, and systolic blood pressure. The results indicate that there is little robust evidence that a higher dairy intake has detrimental effects on markers of cardiometabolic health.

## Introduction

Milk products from cows and other mammals are major components of traditional Western diets, especially in cold climates. In the United States, the recommended intake of milk or equivalent portions of cheese, yogurt, or other dairy products is 3 servings per day for adults and children aged 9 y or older. This amount is substantially higher than the current average intake among adults of 1.6 servings/d [1]. Also, in several other Western countries, 3 daily servings are recommended for adults [2]. The recommended amount mainly refers to the contribution of dairy to cover calcium requirements and reduce bone fracture risk [3]. However, the evidence on health benefits of a high intake of milk products remains inconsistent, and concerns exist about the risks of possible adverse health effects [3]. Moreover, the health effect of different types of dairy products such as naturally fermented products (e.g., yogurt or kefir) and their fat content (low fat: skimmed or semiskimmed products compared with full fat: products with its natural fat content) needs further investigation.

Several previously published systematic reviews of prospective observational studies showed that each daily serving increase in dairy was not associated with adiposity or weight gain [4] but was associated with a lower risk of hypertension [5], type 2 diabetes [6,7], and stroke [8]. For each daily serving increase in total dairy, full-fat dairy, low-fat dairy, milk, cheese, and yogurt, no association with the risk of coronary heart disease was observed [8,9]. Prospective observational studies provide many insights into diet–disease relationships and etiological research questions (e.g., by investigating an exposure such as the amount of dairy intake in relation to the incidence of a specific disease, such as type 2 diabetes). However, randomized controlled trials (RCTs), if well-designed and conducted, can give more robust answers to the research questions they address and are, hence, recommended as the preferred methodology for causal inference [10]. Several systematic reviews and pairwise meta-analyses of RCTs investigating dairy intake are also available, showing no effects on blood lipids [[11], [12], [13]], anthropometric markers [14], and systolic blood pressure [13,15] and inconsistent results on glycemic control [12,16,17]. Compared with the above-described pairwise meta-analyses, a network meta-analysis (NMA) enables a simultaneous analysis of all potential intervention options in a single approach. This offers a possibility to make quantitative comparisons of interventions that have not been directly compared in RCTs by using direct (i.e., from trials comparing dairy types directly: e.g., milk compared with yogurt) and indirect (i.e., from a connected root through ≥1 intermediate comparators) evidence. To the best of our knowledge, no NMA has been conducted to date that simultaneously compared the isocaloric effects of different types of dairy products and different amounts of fat on anthropometric outcomes, blood lipids, glycemic control, or systolic blood pressure.

Therefore, this systematic review with NMA aimed to investigate the comparative effects of dairy intake (e.g., control/low dairy; high dairy; low-fat, high dairy; and full-fat, high dairy) and specific dairy products (e.g., milk, yogurt, kefir, and cheese) on markers of cardiometabolic health in the general healthy adult population.

## Methods

We report this systematic review with NMA according to the PRISMA Extension for Network Meta-analyses (PRISMA-NMA) checklist [18] and the PRISMA Statement for Reporting Literature Searches in Systematic Reviews (PRISMA-S) [19]. The protocol of this study was predefined and registered in the International Prospective Register of Systematic Reviews (PROSPERO; registration number CRD42022303198). Deviations from the study protocol are reported in Supplemental Table 1.

## Systematic literature search

We conducted a comprehensive literature search in 3 electronic databases [MEDLINE (through OVID), Cochrane Central Register of Controlled Trials (through CRSO), and Web of Science (through Clarivate)] from inception to 23 September 2022. The search strategy combined 3 search blocks on “dairy products,” “outcomes” (e.g., cardiovascular diseases, hypertension, and weight change), and “study design” (i.e., RCTs) and was developed by an information specialist (KG). No language filter was applied. The detailed search strategies can be found in Supplemental Table 2. In addition, we conducted backward citation tracking on systematic and narrative reviews, identified by our searches, and screened the reference lists of all included studies.

## Eligibility criteria

We included studies in this systematic review fulfilling the following eligibility criteria:

### Population

We considered studies conducted in the general adult population (age 18 y or older). Studies focusing on children and adolescents, pregnant women, or patients with chronic diseases (e.g., cancer, chronic kidney disease, cardiovascular disease, and type 2 diabetes) were excluded.

### Intervention

We considered interventions focusing on the intake/consumption of dairy products (e.g., total dairy, full-fat dairy, low-fat dairy, and naturally fermented milk products). Nonbovine milk and dairy products (e.g., from sheep, goats, buffalos, and camels), milk/protein isolates (e.g., whey or casein), capsules, fortified dairy products (e.g., fortified with vitamin D, plant sterols/stanols, prebiotics, probiotics, or omega-3 fatty acids), and fermented milk products with additional microbiota strains (beyond those naturally occurring) were excluded.

### Comparator

We considered the intake of other dairy products, diets low in dairy intake, or usual diets as comparators. Studies were excluded if energy intake differed between the intervention and control arms within a RCT. Co-interventions (e.g., physical activity and calorie restriction) were allowed as long as they were balanced across the study arms within a RCT.

### Outcomes

As markers of cardiometabolic health, we considered anthropometric outcomes [body weight (in kilograms), BMI (in kilograms per squared meter), fat mass (in kilograms), and waist circumference (in centimeters)]; blood lipids [low-density lipoprotein (LDL) cholesterol (in millimoles per liter), high-density lipoprotein (HDL) cholesterol (in millimoles per liter), and triglycerides (in millimoles per liter)]; markers of glycemic control [fasting glucose (in millimoles per liter) and glycated hemoglobin (HbA1c; in percentages)]; and systolic blood pressure (millimeters of mercury). In addition, we considered dietary adherence measured by energy intake (in kilocalories per day) and further markers such as consumed dairy servings per day or counting empty packaging.

### Study design

We included RCTs with a parallel or crossover design. Crossover trials were considered for NMA only if data from the first intervention period were available to avoid potential carryover effects [20]. Regarding several of the chosen outcomes (e.g., glycated hemoglobin or body weight) [21] and the corresponding time needed for a response to a dietary intervention, we included RCTs of at least 12 wk of intervention.

## Study selection

After deduplication of search hits using Endnote 20 (Clarivate), 2 reviewers from a group of 4 (EK, JM, JS, LS) screened each title/abstract and full text of potentially eligible studies independently. On the full-text level, the reasons for exclusion were recorded. Any disagreements were resolved by discussion or with the help of a third reviewer (LS) if no agreement could be reached. The screening process was implemented using Covidence (Veritas Health Innovation) and was piloted with a set of 30 records that all involved reviewers screened.

## Data extraction

After identifying eligible articles, 2 reviewers from a group of 3 (EK, IR, JS) extracted the data independently in a piloted data extraction form (Microsoft Excel). Conflicts were solved by discussion or a third reviewer (EK, IR, JS) if no agreement could be reached. We extracted data on study characteristics [i.e., first author, publication year, study location (country), study design (parallel or crossover), duration (study, intervention, and follow-up) and sample size (n randomized)], participant characteristics [i.e., percentage of female, mean age, BMI, and health status (e.g., metabolic syndrome, overweight or obesity)], intervention characteristics [i.e., type of dairy, dose, provision, assessment and degree of compliance, and balanced co-interventions], study funding, conflicts of interest, and outcomes. For all outcomes, we extracted (ANCOVA-adjusted) mean postvalues or change scores and standard deviations (SD). If both, postvalues and change scores were available, we preferred postvalues for the analysis. In case a study did not report the mean and SD, we calculated values from the corresponding median, standard error, or interquartile range [22,23]. If studies reported the relevant data only in figures, we used the Web Plot Digitizer (https://automeris.io/WebPlotDigitizer/) for data extraction. For the analyses, data on LDL and HDL cholesterol, triglycerides, and fasting blood glucose given in milligrams per deciliter were converted to millimoles per liter [24,25] and data on energy intake from kilojoules per day in kilocalories per day [26]. If the percentage fat mass was reported, we calculated absolute fat mass (in kilograms) if data on body weight were also available. In the case of insufficient or missing information, we made 2 attempts to contact the corresponding study authors by e-mail.

## Risk of bias assessment

Two reviewers of a group of 3 (EK, JM, JS) assessed the risk of bias of each included study independently, and any disagreements were resolved by consensus. We used the Cochrane Risk of Bias tool 2.0 (RoB 2) for parallel RCTs [27] and its test version for crossover trials [28] to evaluate the risk of bias. RoB 2 considers 5 domains: bias arising from the randomization process, bias due to deviations from the intended interventions, bias due to missing outcome data, bias in the measurement of the outcome, and bias in the selection of the reported results. In the RoB assessment of crossover trials, the additional domain, “bias arising from period and carryover effects,” was evaluated. We were mainly interested in the effects of assignment to intervention. We judged each domain and the overall risk of bias as low RoB, some concerns, or high RoB. Additional guidance for the RoB 2 assessment is provided in Supplemental Table 3.

## Statistical analysis

This systematic review is a network of interventions. We initially performed a pairwise random-effects meta-analysis to estimate all possible pairwise relative effects for each outcome of interest. A frequentist NMA was performed to evaluate the summary intervention effects of each outcome, using the R package “netmeta” [[29], [30], [31]]. Continuous outcome data in both pairwise meta-analysis and NMA were synthesized using mean differences (MDs) with their 95% confidence intervals (95% CIs). We used the random-effects model owing to the expected between-study variability (heterogeneity) in the measurement of outcomes. The between-study heterogeneity of the intervention effects within each treatment comparison was assessed by *I*^2^ [32], and the magnitude of the between-study variance (τ^2^) was estimated using the generalized DerSimonian and Laird estimator and the Q-profile approach [33,34]. NMA validity depends on the consistency assumption. We evaluated each network for inconsistency globally using the random-effects design-by-treatment interaction model [35,36]. The network structure for each outcome was illustrated using network plots, and NMA results are presented using forest plots and league tables. Interventions were ranked using *P* scores, a frequentist version of the surface under the cumulative ranking curve [37]. *P* scores are values between 0 and 1, where a value of 1 means that a treatment always ranks best and a value of 0 means that a treatment ranks always worst. When the number of studies was more than 10, a comparison-adjusted funnel plot was drawn to assess publication bias and small study effects [38,39].

We defined the network nodes in 2 different ways. The first network type named “total dairy intake” consisted of the following nodes: control/low dairy; high dairy; low fat, high dairy; and full fat, high dairy. High dairy was defined by at least 3 servings/d, or an equal amount in grams per day. Control/low dairy was defined as the usual diet or a diet with 0–2 servings/d (mostly 0–1 serving), or an equal amount in grams per day. The definitions for low-fat and full-fat dairy were based on the common fat contents of dairy products: low-fat dairy includes skimmed or semiskimmed products (e.g., low-fat milk: 1.5% fat), and full-fat dairy products have its natural fat content (e.g., full-fat milk: 3.25%). The second network type called “dairy product intake” consists of the nodes control, milk, yogurt, kefir, and mixed dairy products (at least to different dairy products).

Sensitivity analyses were performed for the network of total dairy intake by further dividing the node control/low dairy into 2 separate nodes and by excluding RCTs rated as a high RoB for the respective outcomes. Subgroup analyses were conducted on the network of total dairy intake according to the type of diet (hypocaloric compared with eucaloric/ad libitum diet).

## Certainty of evidence

We evaluated the certainty of the evidence for 7 prioritized outcomes body weight, BMI, fat mass, waist circumference, LDL cholesterol, triglycerides, and systolic blood pressure according to the Grading of Recommendations Assessment, Development and Evaluation (GRADE) approach for NMA [40] based on the networks of total dairy intake and dairy product intake. One reviewer (EK) rated the certainty of evidence in each of the direct, indirect, and network estimates for each outcome. The results were reviewed by a second reviewer (LS); any disagreements were solved by a discussion. Direct evidence was rated based on the RoB, inconsistency, indirectness, and publication bias (if at least 10 studies were available). If the certainty of direct evidence was high and its contribution was at least as much as that of the indirect evidence, we did not rate the indirect evidence [40]. If the rating of indirect evidence was necessary, we used the certainty of direct estimates to inform indirect estimates considering the lowest of the ratings of the 2 direct comparisons forming the most dominant first-order loop. In the presence of serious intransitivity, we rated down the certainty of the indirect estimate. To address the certainty of network estimates, we compared the ratings for direct and indirect estimates. The estimate with the higher certainty was chosen and rated down if incoherence and/or imprecision were detected [40]. For the assessment of imprecision, we used the null effect for all outcomes except systolic blood pressure levels. In this study, we defined 2 mm Hg as a small effect [41]. All decisions to downgrade the certainty of evidence are given by informative footnotes. Evidence profiles were created to summarize the evidence in a transparent and informative format [42]. GRADE classifies the certainty of evidence by 4 levels: high, moderate, low, and very low.

## Results

**FIGURE 1:**
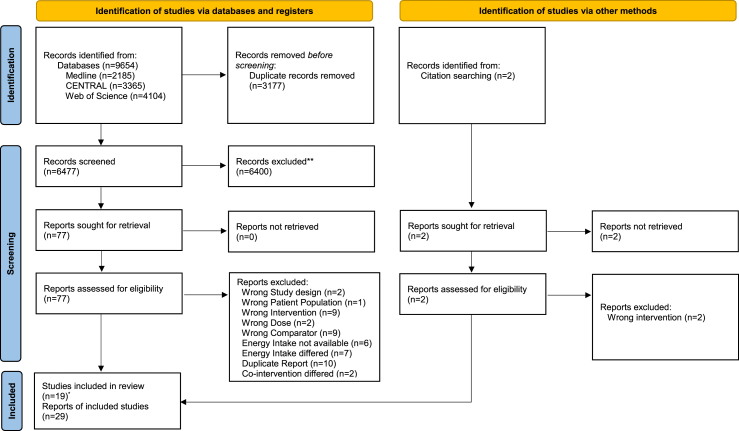
PRISMA 2020 flow diagram. ∗Two studies were reported in 1 record From: Page MJ, McKenzie JE, Bossuyt PM, Boutron I, Hoffmann TC, Mulrow CD, et al. The PRISMA 2020 statement: an updated guideline for reporting systematic reviews. BMJ 2021;372:n71. doi: 10.1136/bmj.n71. For more information, visit: http://www.prisma-statement.org/

The database searches resulted in 9654 hits. After deduplication, we screened the eligibility of 6477 titles/abstracts and of 77 full texts. Finally, we included 19 RCTs [[43], [44], [45], [46], [47], [48], [49], [50], [51], [52], [53], [54], [55], [56], [57], [58], [59], [60]] with 29 reports (one publication reported on 2 different RCTs) (Supplemental Table 4). The reasons for exclusion of full texts are given in Supplemental Table 5. The flow diagram of the search and screening process is depicted in Figure 1.

## Study and participant characteristics

The Table presents a summary of the main characteristics of included studies and participants; detailed information can be found in Supplemental Table 6. Eleven of the included RCTs were conducted in North America [45,48,49,51,52,[54], [55], [56],[58], [59], [60]], 4 in Scandinavia [44,47,50,57], 2 in Asia [46,53], and 1 in Turkey [43]. All but 1 RCT—a crossover trial [51] (not considered for NMA)—had a parallel design. The study duration lasted from 12 to 51.6 wk (Table).

**TABLE:** Summary of study and participant characteristics

	*n* (%)
Geographic location	
North America	12 (63.2)
Europe	5 (26.3)
Asia	2 (10.5)
Design	
Parallel	18 (94.7)
Crossover^1^	1 (5.3)
Intervention duration (mo)	
3	6 (31.6)
>3 to ≤6	10 (52.6)
>6	3 (15.8)
Sample size	
<50	7 (36.8)
50–100	7 (36.8)
>100	5 (26.4)
Sex	
Female	4 (21.0)
Male	1 (5.3)
Mixed	14 (73.7)
Age (y), mean	
≤35	4 (21.1)
35 to <65	15 (78.9)
≥65	0
Health status^2^	
Overweight/obesity	12 (63.2)
Metabolic syndrome	5 (26.3)

1 Not considered in the network meta-analysis.

2 Multiple mentioning possible.

Overall, the eligible RCTs included 1427 participants. The sample size ranged from 25 [45] to 213 [53]. The mean age of the participants ranged from 20.1 [48] to 64 y [52]. Four studies focused on females only [45,46,48,54] and 1 solely on males [53]. The other studies were conducted in mixed samples with an average proportion of females ranging from 41.7% to 93.0% [43,44,47,[49], [50], [51], [52],[55], [56], [57], [58], [59], [60]]. The baseline mean BMI ranged from 22 [45] to 35 kg/m^2^ [55]. Twelve studies included only participants with overweight/obesity based on the BMI [44,46,47,49,[54], [55], [56], [57], [58], [59], [60]].

Information on funding sources of included RCTs and declarations of potential competing interest of study authors can be found in Supplemental Table 7. Twelve of the included studies were at least partially funded by the National Dairy Council (52.6%) or by industry (10.5%).

## Intervention characteristics

Supplemental Table 8 presents the intervention characteristics of the included RCTs. Most of the RCTs (*n* = 17) compared 2 interventions arms, whereas 2 trials used a multiarm design with 3 interventions arms [48,52]. In 16 RCTs, interventions of high-dairy consumption (mostly defined by 3 or more servings/d) were compared with a low dairy or a control intervention [44,45,[47], [48], [49],^51^,[53], [54], [55], [56], [57], [58], [59], [60], [61]]. Seven interventions focused on low-fat dairy [45,47,48,[50], [51], [52],^54^] and 1 on full-fat dairy products [52], whereas 11 interventions used mixed products or did not specify the fat content [44,49,50,53,[55], [56], [57], [58], [59], [60]]. Five trials investigated the effects of specific dairy products (i.e., milk, kefir, yogurt, and cheese) compared with each other or to a control group [43,[45], [46], [47],^50^]. As co-intervention for all investigated groups, 9 RCTs used a hypocaloric diet (*n* = 9) [44,49,[53], [54], [55], [56],[58], [59], [60]], whereas the other trials focused on eucaloric (*n* = 9) [43,[45], [46], [47], [48],^50^,^51^,^57^,^59^] or ad libitum diets (*n* = 1) [52].

## Risk of bias

The results of the RoB assessment are provided in Supplemental Figures 1 and 2. A total of 66 RoB assessments were performed with separate assessments for anthropometric outcomes (*n* = 21) [[43], [44], [45], [46], [47], [48], [49], [50], [51], [52], [53], [54], [55], [56], [57], [58], [59], [60]], blood markers (*n* = 15) [43,44,46,47,[50], [51], [52], [53],[55], [56], [57], [58], [59]], blood pressure (*n* = 13) [43,44,46,47,[50], [51], [52], [53],[57], [58], [59], [60]], and energy intake (*n* = 17) [20,[43], [44], [45], [46], [47], [48], [49], [50], [51], [52], [53],^55^,^57^,^59^,^60^]. Across all outcome domains, no RCT was judged to have an overall low RoB. For 87.9% of the outcomes, the overall rating of RCTs was “some concerns”. A high RoB was identified for 3 RCTs in the outcome domain blood markers, for 2 RCTs each in the domains anthropometry and systolic blood pressure, and for 1 RCT for energy intake. In the domains “risk of bias arising from the randomization process” (65.1%), “deviations from the intended interventions” (89.3%), and “selection of the reported results” (95.4%) RCTs were rated for most outcomes with “some concerns.” In the domains “missing outcome data” (66.7%) and “measurement of the outcome” (74.2%), ratings mainly refer to a low RoB.

## Anthropometric outcomes

**FIGURE 2:**
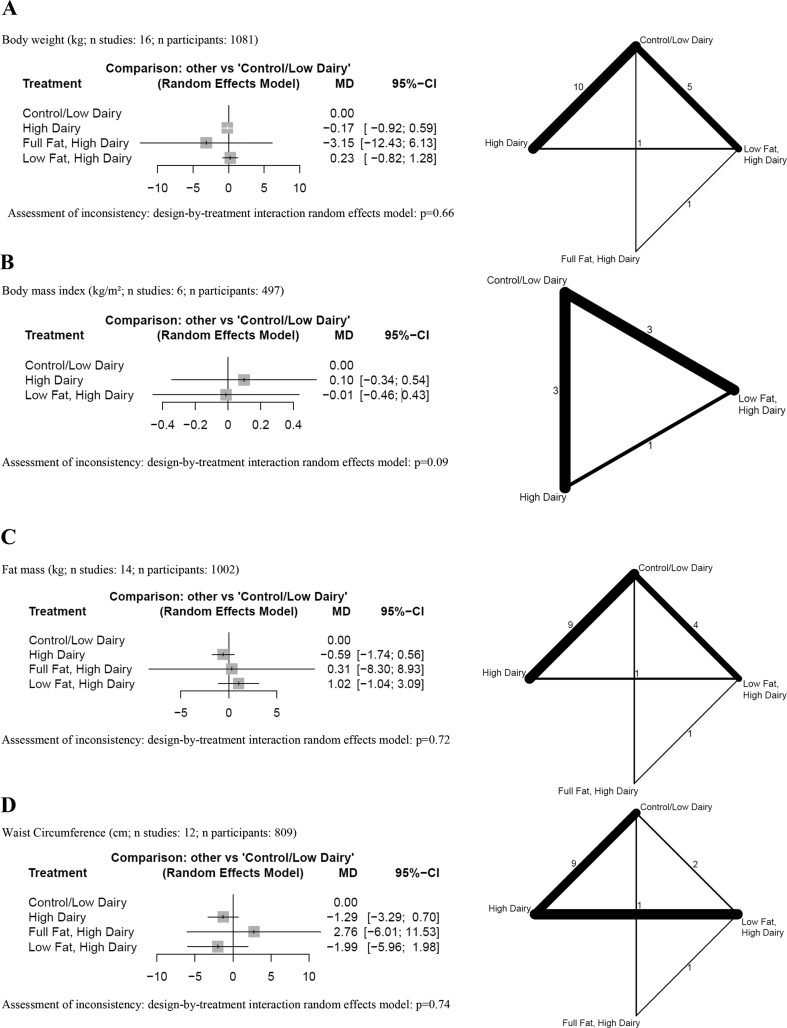

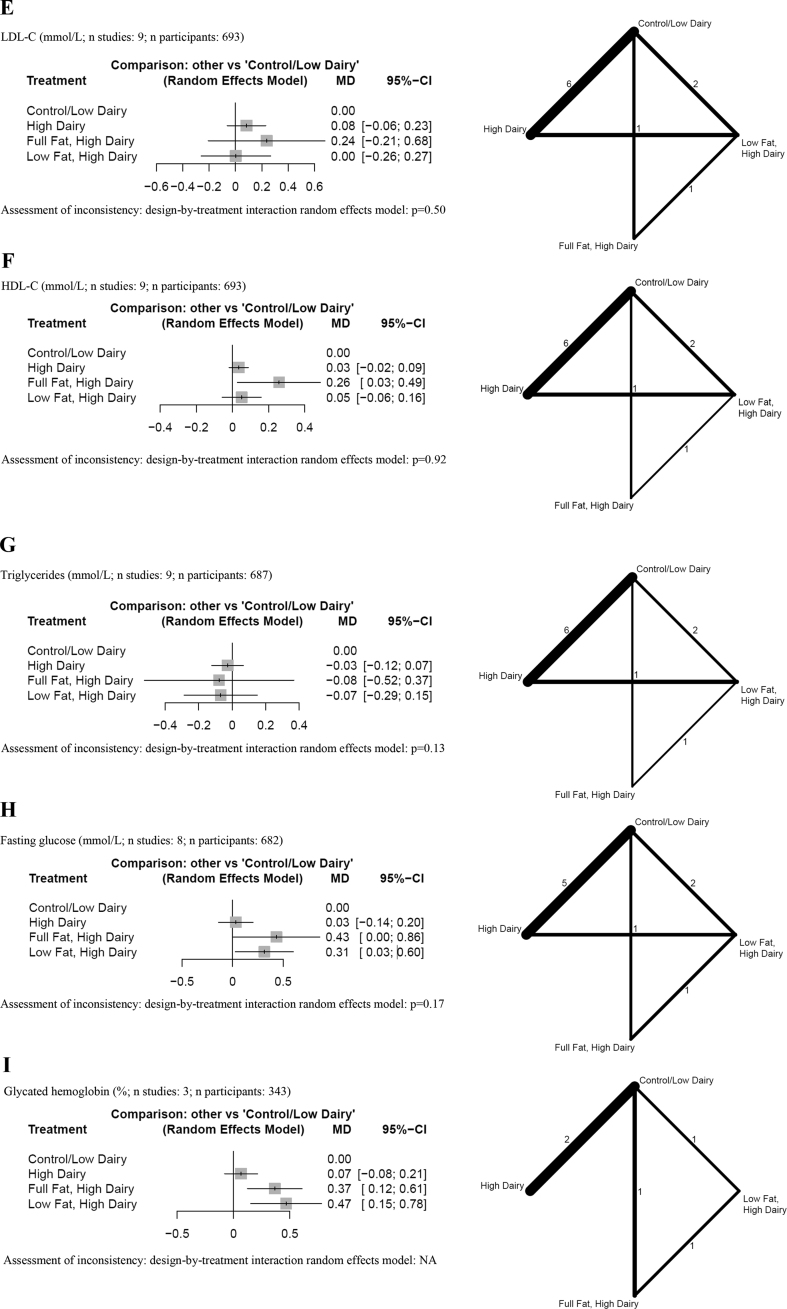

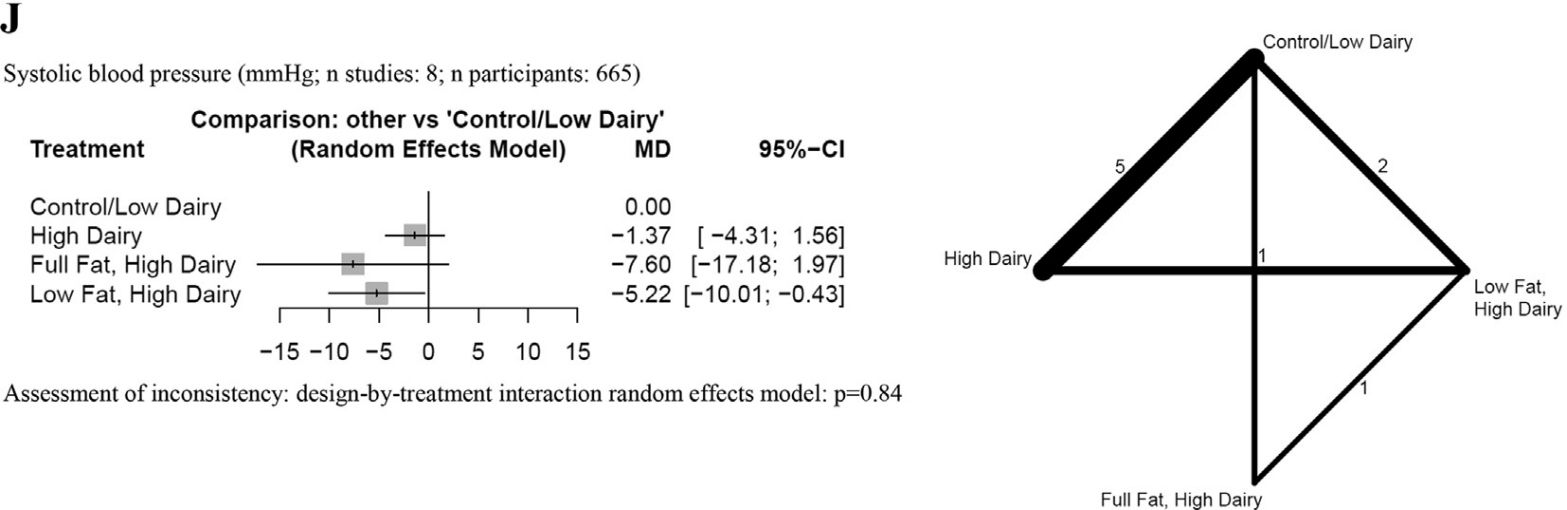
Forest plots summarizing MDs with 95% CIs and network plots for (A) body weight, (B) BMI, (C) fat mass, (D) waist circumference, (E) LDL cholesterol, (F) HDL cholesterol, (G) triglycerides, (H) fasting glucose, (I) glycated hemoglobin, and (J) systolic blood pressure as estimated from the network meta-analysis on total dairy intake with a combined control/low-dairy group. Control/low dairy, usual diet or a diet with 0–2 dairy servings/d or an equal amount in grams per day; high dairy, ≥3 dairy servings/d or an equal amount in grams per day; full-fat dairy, dairy products with its natural fat content; low fat dairy, skimmed or semiskimmed dairy products. Network plots: line width, weight from random-effects model comparing 2 treatments; numbers: the number of studies directly comparing treatments. MD, mean difference; NA, not applicable.

The results of the NMAs are presented in Figure 2A–D. We observed no detrimental effects of a high-dairy diet (irrespective of fat content), low-fat dairy or full-fat dairy diet over a control group (low in dairy) on body weight, BMI, fat mass, and waist circumference (low certainty) (Supplemental Tables 9–12) [44,45,[47], [48], [49], [50],[52], [53], [54], [55], [56], [57], [58], [59], [60]].

Regarding the type of dairy (Supplemental Figures 3A–C and 4A), no differences between milk, yogurt, kefir, mixed dairy products, and control were observed for all anthropometric outcomes (very low and low certainty) (Supplemental Tables 13–16) [[43], [44], [45], [46], [47], [48], [49],[52], [53], [54], [55], [56], [57], [58], [59], [60]]. The pairwise comparisons between milk and yogurt may result in a small reduction in waist circumference, favoring yogurt (MD: −3.47 cm; 95% CI: −6.92, −0.02; low certainty) (Supplemental Table 16) [46].

## Blood lipids

Results of the NMA are described in Figure 2E–G. We observed no detrimental effects of a high-dairy (irrespective of fat content), low-fat dairy, or full-fat dairy diet over a control group (low in dairy) on LDL cholesterol, HDL cholesterol, and triglycerides (low certainty) (Supplemental Tables 17–19) [44,47,50,52,53,[55], [56], [57], [58]]. Full-fat dairy may increase HDL cholesterol compared with a control diet (MD: 0.26 mmol/L; 95% CI: 0.03, 0.49 mmol/L) (Supplemental Table 19A).

Regarding the type of dairy (Supplemental Figure 3D–F), the comparison between yogurt and milk may result in a small reduction in triglycerides (MD: −0.38 mmol/L, 95% CI: −0.73, −0.03 mmol/l; low certainty) (Supplemental Table 20), and an improvement of HDL cholesterol (MD: 0.19 mmol/L, 95% CI: 0.00, 0.38 mmol/L) (Supplemental Table 21A), favoring yogurt. For all other comparisons, no differences were observed for LDL cholesterol, HDL cholesterol, and triglycerides (low certainty) (Supplemental Tables 20–22) [43,44,46,47,52,53,[55], [56], [57], [58]].

## Glycemic control

We observed no detrimental effects of a general high-dairy (without specification of fat content) diet over a control group (low in dairy) on fasting glucose (Figure 2H and Supplemental Table 19B) [44,47,50,52,53,[55], [56], [57]]. However, both low-fat dairy and full-fat dairy increased fasting glucose (MD: 0.31–0.43 mmol/L) (Supplemental Table 19B) and glycated hemoglobin levels (MD: 0.37%–0.47%) compared with a control diet (Figure 2I and Supplemental Table 19C).

Regarding the type of dairy, no differences between milk, yogurt, kefir, mixed dairy products, and control diets were observed for fasting glucose and glycated hemoglobin levels (Supplemental Figures 3G and 4B, Supplemental Table 21B and 23) [43,44,46,47,52,53,[55], [56], [57]].

## Blood pressure

The results of the NMA and the pairwise comparisons are described in Figure 2J and Supplemental Table 24. We observed no detrimental effects of a high-dairy diet (irrespective of fat content), low-fat dairy diet, or full-fat dairy diet over a control group (low in dairy) on systolic blood pressure (low certainty) [44,47,50,52,53,57,59,60]. Rather, we were able to show that low-fat dairy may result in a reduction for systolic blood pressure (MD: −5.22 mm Hg; 95% CI: −10.01, −0.43 mm Hg; low certainty) compared with a control low dairy diet. A similar point reduction was observed when a full-fat dairy diet was compared with a low dairy diet, but the effect was not statistically significant (MD: −7.60 mm Hg; 95% CI: −17.18, 1.97 mm Hg; low certainty).

Regarding the type of dairy, no differences between milk, yogurt, kefir, mixed dairy products, and control diets were observed for systolic blood pressure levels (very low and low certainty) (Supplemental Figures 3H and Supplemental Table 25) [43,44,46,47,52,53,57,59,60].

## Dietary adherence

The assessment of dietary adherence was reported in all RCTs (Supplemental Table 8). All RCTs used some kind of dietary record (e.g., 3-d dietary records, weighing records, or food frequency questionnaires) to monitor diets and/or counted the consumed dairy servings (*n* = 10) [43,[48], [49], [50], [51], [52], [53],[55], [56], [57]]. Furthermore, counting of empty packaging (*n* = 4) [46,47,52,60] or monitoring of body weight (*n* = 1) [54] were described as strategies to assess adherence. Most of the trials reported adequate dietary adherence (*n* = 15) [[43], [44], [45], [46], [47],^49^,^50^,[52], [53], [54],[56], [57], [58], [59]]. Two RCTs reported that approximately one-third of the participants did not meet adherence goals [55,60]; 2 further RCTs acknowledged potential adherence problems in the discussion [48,51].

Energy intake was assessed at the baseline and the end of the intervention in 10 RCTs [[43], [44], [45], [46], [47], [48], [49],^53^,^54^,^57^], whereas others reported energy intake during the intervention [[50], [51], [52],^55^,[58], [59], [60]]. The results of the NMA are described in Supplemental Figure 5 and Supplemental Table 19D. We observed slight differences in energy intake between some comparisons. Regarding the type of dairy, no differences between milk, yogurt, kefir, mixed dairy products, and control diet were observed for the energy intake (Supplemental Figure 3I and Supplemental Table 21C) [[43], [44], [45], [46], [47], [48], [49],[52], [53], [54], [55],[57], [58], [59], [60]].

## *P* scores and rankings

*P* scores are presented in Supplemental Tables 26 and 27. We did not identify any intervention that ranked the best across the outcomes for total dairy intake and dairy product intake.

## Inconsistency

The global test for inconsistency (i.e., design-by-treatment interaction random-effects model) indicated no evidence of statistically significant inconsistency in the NMA for all outcomes for “total dairy intake” networks. For “dairy product intake” networks, the global test for inconsistency indicated no evidence of statistically significant inconsistency in the NMA for the energy intake outcome, but we could not evaluate consistency in the NMAs for all other outcomes because they were star-shaped networks or were disconnected networks.

## Dissemination bias

The visual assessment of comparison-adjusted funnel plots suggested no evidence of small study effects for none of the outcomes (Supplemental Figures 6 and 7).

## Sensitivity and subgroup analyses

Sensitivity analyses for the networks of total dairy intake with separate control and low dairy groups are presented in Supplemental Figure 8. For anthropometric outcomes and blood lipids, the results were very similar to the main analysis. The increase in fasting glucose by full-fat and low-fat dairy lost significance (MD: 0.35 mmol/L; 95% CI: −0.19, 0.88 mmol/L; and MD: 0.24 mmol/L; 95% CI: −0.13, 0.62 mmol/L, respectively) when compared with that by the control diet. Similar results were obtained for the reduction of systolic blood pressure by low-fat dairy (MD: −2.71 mm Hg; 95% CI: −7.23, 1.81 mm Hg) compared with that by the control diet. Sensitivity analyses excluding RCTs with a high RoB for the respective outcomes were similar to the results of the primary analyses for interventions on the total dairy intake (Supplemental Figure 9). Sensitivity analyses were not applicable to BMI, fasting glucose, and glycated hemoglobin levels because no study was rated with a high RoB for the respective outcomes.

A subgroup analysis by the type of diet (hypocaloric; eucaloric/ad libitum diet) are presented in Supplemental Figure 10. Owing to the low number of studies, the network size (the number of nodes) differed between the subgroups. For most outcomes, no major differences compared with the primary analyses were found. For HDL cholesterol, only RCTs with high-dairy eucaloric/ad libitum diets showed a significant increase in concentrations compared with the control diets (MD: 0.15 mmol/L; 95% CI: 0.04, 0.26 mmol/L), whereas high-dairy, hypocaloric diets did not affect HDL cholesterol values (MD: 0.00 mmol/L; 95% CI: −0.05, 0.06 mmol/L). For triglycerides, the direction of the effect between high-dairy eucaloric/ad libitum diets (MD: 0.21 mmol/L, 95% CI: −0.14, 0.57 mmol/L) and high-dairy, hypocaloric diets (MD: −0.05 mmol/L; 95% CI: −0.15, 0.05 mmol/L) compared with that of control was opposite.

## Discussion

This is, to the best of our knowledge, the first NMA to evaluate the effects of different dairy products and different dairy fat contents on anthropometric outcomes, blood lipids, blood pressure, and glycemic control, including 19 RCTs and comprising 1427 participants.

In summary, a higher dairy intake (irrespective of fat content) showed no detrimental effects on body weight, BMI, fat mass, and waist circumference. Regarding the type of dairy, except for the comparison milk with yogurt, which showed a beneficial effect of yogurt on the waist circumference, no effects on anthropometric measures were observed. Similarly, no detrimental effect of a higher dairy intake (irrespective of fat content) on blood lipids and systolic blood pressure was found. Full-fat dairy products may increase HDL cholesterol compared with a control diet, whereas both low-fat and full-fat dairy products showed a beneficial effect on systolic blood pressure levels. When yogurt was compared with milk, a higher yogurt intake improved triglycerides and HDL cholesterol. Although, we observed, for glycemic control outcomes, no differences between the different dairy products; interstingly, both low-fat and full-fat dairy may increase fasting glucose and glycated hemoglobin levels. For most of the comparisons, this NMA yielded no important effects, and the certainty of evidence was mainly rated as low.

## Comparison with other studies

An NMA of 66 RCTs (duration ≥4 wk) comparing 10 food groups and enrolling 3595 adult participants indicated that dairy products were ranked worse than plant-based foods such as nuts, legumes, or whole grains for improving the markers of cardiometabolic health [62].

### Anthropometric outcomes

In line with our findings, a pairwise meta-analysis of 37 RCTs (duration ≥4 wk) did not show differences in body weight and fat mass change between the dairy intervention (0.5 to 5.24 servings/d) and control groups [63]. However, the results of our subgroup analyses did not confirm that high-dairy consumption in the absence of caloric restriction may slightly increase body weight (by 0.36 kg), whereas in the presence of caloric restriction, it may slightly reduce the waist circumference (−2.18 cm) and fat mass (−0.56 kg), as previously shown in the meta-analysis by Geng et al. [63]. In contrast to our findings, another meta-analysis showed that a higher intake of both low-fat and full-fat dairy products (duration, ≥4 wk) leads to a slight weight gain (0.41–0.82 kg) (12). A recently published overview of systematic reviews [14] on interventions for increased dairy intake (duration, ≥4 wk) concluded that the total dairy intake without energy restriction does not affect the body weight, waist circumference, and fat mass. However, increased dairy intake in combination with energy restriction was associated with the weight loss and reduced fat mass. Overall, the abovementioned effects of a high-dairy consumption on anthropometric outcomes seem to be of little clinical relevance.

### Blood lipids

In agreement with our findings, Derakhshandeh-Rishehri et al. [11] observed no detrimental effects of dairy foods on blood lipids when investigating both interventions lasting <12 and ≥12 wk and with ≤3 and >3 dairy servings. Notably, in this systematic review, the energy intake of the included trials was not considered. In contrast to our findings, a further meta-analysis excluding studies with caloric restriction showed no HDL cholesterol increasing effect of full-fat dairy products for interventions of at least 4 wk of duration [12].

### Blood pressure

In a Mendelian randomization study, the weak inverse association between dairy intake and systolic blood pressure level in observational studies was not supported by a comprehensive instrumental variable analysis and systematic review of existing RCTs with interventions mainly providing 3 or more dairy servings and durations of least 1 wk (*n* = 8; *n* = 5 with crossover design) [15]. Similar results were found in a second work including 12 RCTs (duration, ≥4 wk; various definitions of dairy intake) [13]. In contrast to this study, the abovementioned studies did not consider the degree of fat content. In this NMA, 10 RCTs were included, and we could detect a blood pressure–lowering effect of low-fat and full-fat dairy diet, which needs to be interpreted with caution because the certainty of evidence was rated as low.

### Glycemic control

In a systematic review by O'Connor et al (intervention duration, ≥1 wk) [16], similar to our findings, fasting glucose was positively associated with an elevated dairy intake (mainly low-fat and ≥3 servings) by 0.07 mmol/L; whereas, in disagreement with our findings, the glycated hemoglobin level was negatively associated with a higher dairy intake by −0.07%. The findings were driven by non–energy-restricted interventions. However, most included studies had a high RoB and the certainty of evidence was very low for fasting glucose and low for glycated hemoglobin levels [16].

## Possible biological mechanisms

### HDL cholesterol

The HDL cholesterol increasing effects of full-fat dairy could be due to its high content of saturated fatty acids, with the dominant fatty acids such as myristic acid (C14:0) and palmitic acid (C16:0), which were shown to raise HDL cholesterol levels when substituted for carbohydrates [64]. The HDL-raising effects of saturated fatty acids were reported to depend on the chain length and are greater with a shorter length [65].

### Blood pressure

Regarding the beneficial effects of low-fat and full-fat dairy products on systolic blood pressure levels, in meta-analyses of prospective observational studies, an inverse association between each 200-g/d increase for both low-fat and full-fat dairy products and risk of hypertension was observed [5]. Calcium, potassium [66], or lactotripeptides [67] as components of dairy products may contribute to the described antihypertensive effect. Calcium and potassium are, among others, responsible for an ionic balance of vascular membranes and regulate vasodilatation [66]. For lactotripeptides, an inhibition of the vasoconstrictive effect by the angiotensin I–converting enzyme is suggested as a potential mode of action [68].

### Glycemic control

One potential mechanism of action for the detrimental effects on fasting glucose and glycated hemoglobin levels could be a physiologic response to dairy-rich meals. The insulinotropic effect of dairy is higher than expected based on their modest glycemic indexes [69] and may be triggered by branched-chain amino acids of dairy foods. It is assumed that these amino acids act directly on the pancreatic β cells and, additionally, promote the release of the incretin glucagon-like peptide-1 from intestinal L-cells, as shown in vitro [70,71]. Repeated postprandial hyperinsulinemia induced by a regular dairy intake may foster insulin resistance [72].

### Beneficial effects of yogurt

We observed some beneficial health effects (improvement in waist circumference and blood lipids) of yogurt consumption compared with those from milk intake. This may be attributed to changes in nutritive and bioactive properties of dairy products during fermentation: Bioactive compounds, such as peptides with antihypertensive, antimicrobial, antioxidative, and immune-modulatory activities may be synthesized or released [73,74]. Lactic acid bacteria may produce bacteriocins, biogenic amines, and exopolysaccharides [75]. Furthermore, fermentation-associated bacteria can synthesize several B vitamins (e.g., folate, riboflavin, and vitamin B12) and, thereby, increase the nutritive content of dairy products [76,77]. Finally, the content of conjugated linoleic acid as a component of milk fat with known anti-inflammatory, anti-atherogenic, and antioxidative properties may increase during fermentation [78].

## Implications

### Recommended intake

According to Weaver [2], dietary recommendations for dairy mainly refer to 3 servings/d of milk, yogurt, or cheese in several countries such as Australia, China, France, Switzerland, and the United States. Regarding cardiometabolic health, total dairy consumption has not been clearly linked to the outcomes such as weight control, diabetes, or cardiovascular disease [3], which was similar to our findings. Furthermore, no clear advantage of consuming low-fat over full-fat dairy products has been found in this study and in other studies [3]. Based on the suggestion by Willett and Ludwig [3], the optimal intake of milk for an individual may depend on the overall diet quality.

### Influence of industry funding

A substantial proportion of the included studies was at least partially funded by the National Dairy Council or industry. In a recent systematic review, the authors found no clear evidence of an association between studies with food industry ties and the reporting of favorable results and conclusions compared with studies without industry ties [79].

## Strengths and limitations

This systematic review and NMA has several strengths and limitations that need to be considered. Among the strengths are the application of the NMA methodology, the a priori–deposited protocol, the comprehensive search strategy, RoB assessment, and the GRADE certainty of evidence assessment. Another strength was the concomitant inclusion of several markers of cardiometabolic health known to be well established. Moreover, we used strict inclusion criteria to ensure that dairy intake was the main difference between the study arms and that energy intake was similar between the studies. In all but 2 studies [46,52], the energy intake did not significantly differ between the intervention groups. In the study by Chen et al. [46], all analyses were adjusted for the differences in energy intake. The study by Schmidt et al. [52] was the only RCT using an ad libitum approach, showing a higher mean change in the energy intake in the full-fat dairy group than that in the other groups. However, because the baseline energy intake seemed to be lower in the full-fat dairy group, we considered these differences as not meaningful and decided to include the RCT. The similar results of the subgroup analyses for the hypocaloric approach—without the RCTs of Schmidt et al. [52]—to those of the main analyses support that the slight differences in the energy intake had no major effect.

Limitations of the current NMA are as follows: first, the certainty of evidence was generally rated as low. This was largely driven by RoB and imprecision: overall, 3 RCTs (15.8%) were rated with a high RoB for at least 1 outcome in at least 1 domain. Imprecision was driven by the low sample sizes of the various study arms; hence, the included studies and the meta-analyses may lacked power to detect differences within or between the groups. This might also have contributed to the inconclusive ranking results, preventing the identification of the best intervention across outcomes. Second, owing to the low number of RCTs, it was not possible to conduct several a priori–planned subgroup analyses, including study duration, gender, and geographical location. Third, the study duration was often short (89.5% of the RCTs, <12 mo); therefore, no data on patient-relevant outcomes such as cardiovascular disease, type 2 diabetes, or cancer were available. Fourth, analyses—especially those of dairy products—were limited, resulting in network nodes referring to single trials only. Moreover, regarding dairy products no comparisons with cheese, butter, or curd were possible owing to lacking data. Fifth, differences in the instruments (e.g., dietary record or food frequency questionnaire) and the timing (end of the intervention compared with during intervention) of measuring energy intake as a marker of adherence might explain some of the differences in the analyses of energy intake.

## Conclusions

In summary, a higher dairy intake (irrespective of fat content) showed no detrimental effects on anthropometric outcomes, blood lipids, and blood pressure. However, both low-fat and full-fat dairy improved systolic blood pressure levels but may concomitantly impair the glycemic control. Yogurt improved waist circumference, triglycerides, and HDL cholesterol compared with milk. Overall, it seems that current recommendations for dairy intake did not negatively influence markers of cardiometabolic health. However, because available RCTs mainly focused on total dairy intake, future studies should compare the effects of specific dairy products to generate more robust evidence.

## Author disclosures

EK, JS, MP, JM, KG, IR, RL, LH, MK, CR, and MR report no conflicts of interest. HH is a member of the Advisory Board of the Competence Center for Nutrition of the Bavarian State Ministry for Food, Agriculture and Forestry and a member of the Food-based Dietary Guidelines working group of the German Nutrition Society. LS is a member of the Editorial Board of Advances in Nutrition and a member of the Grading of Recommendations, Assessment, Development and Evaluations (GRADE) working group.

## Funding

This project was funded by the Bavarian State Ministry of Food, Agriculture and Forestry (Bayerisches Staatsministerium für Ern₠ahrung, Landwirtschaft und Forsten [StMELF]).

## Data Availability

This manuscript made use of publicly available data from published studies. Therefore, no original data are available for sharing.
