# Supplementary material for: Effects of Dairy Intake on Markers of Cardiometabolic Health in Adults: A Systematic Review with Network Meta-Analysis
Source: Adv Nutr. 2023 Mar 11;14(3):438–50. doi: 10.1016/j.advnut.2023.03.004 (PMC10201829; doi:10.1016/j.advnut.2023.03.004)
Supplement: Multimedia component 1 [file mmc1.pdf]

Supplementary Material

Effects of dairy intake on intermediate disease markers in adults - a systematic review with network meta-analysis

Eva Kiesswetter[1], Julia Stadelmaier[1], Maria Petropoulou[2], Jakub Morze[3,4], Kathrin Grummich[1,5], Isabelle Roux[1], Roberta Lay[1], Lisa Himmelsbach[6], Martin Kussmann[6], Christine Roeger[6], Malte Rubach[6], Hans Hauner[7,8], Lukas Schwingshackl[1]

[1]Institute for Evidence in Medicine, Medical Center & Faculty of Medicine, University of Freiburg, Freiburg, Germany.

[2]Institute of Medical Biometry and Statistics, Medical Center & Faculty of Medicine, University of Freiburg, Freiburg, Germany.

[3]Department of Epidemiology and Health Promotion, School of Public Health, Center of Postgraduate Medical Education, Warsaw, Poland.

[4]Department of Nutrition, Harvard T.H. Chan School of Public Health, Boston, MA, USA.

[5]Cochrane Germany, Cochrane Germany Foundation, Freiburg, Germany.

[6]Competence Center for Nutrition, Bavarian State Ministry for Food, Agriculture and Forestry, Freising, Germany.

[7]Else Kröner-Fresenius-Center for Nutritional Medicine, ZIEL – Institute for Food and Health, Technical University of Munich, Freising, Germany.

[8]Institute of Nutritional Medicine, School of Medicine, Technical University of Munich, Munich, Germany.

Table of contents

Supplemental Table 1: Deviations from protocol (PROSPERO; registration number CRD42022303198) 5

Supplemental Table 2: Search strategies for all electronic databases 6

Supplemental Table 3: Additional risk of bias guidance for the included randomized controlled trials 11

Supplemental Table 4: Reports of the 19 included Randomized Controlled Trials 14

Supplemental Table 5: Reasons for excluding studies at full-text screening (n=48) 17

Supplemental Table 6: Study and Participants' characteristics of included trials (n=19) 18

Supplemental Table 7: Reporting of study funding and conflict of interest statements in included trials (n=19) 21

Supplemental Table 8: Intervention characteristics of included trials (n=19) 24

Supplemental Table 9: GRADE evaluation of body weight (kg) for all comparisons (network total dairy intake) 37

Supplemental Table 10: GRADE evaluation of body mass index (kg/m²) for all comparisons (network total dairy intake) 38

Supplemental Table 11: GRADE evaluation of fat mass (kg) for all comparisons (network total dairy intake) 39

Supplemental Table 12: GRADE evaluation of waist circumference (cm) for all comparisons (network total dairy intake) 40

Supplemental Table 13: GRADE evaluation of body weight (kg) for all comparisons (network dairy product intake) 41

Supplemental Table 14: GRADE evaluation of BMI (kg/m²) for all comparisons (network dairy product intake) 42

Supplemental Table 15: GRADE evaluation of fat mass (kg) for all comparisons (network dairy product intake) 43

Supplemental Table 16: GRADE evaluation of waist circumference (cm) for all comparisons (disconnected; network dairy product intake) 44

Supplemental Table 17: GRADE evaluation of LDL-C (mmol/L) for all comparisons (network total dairy intake) 45

Supplemental Table 18: GRADE evaluation of triglycerides (mmol/L) for all comparisons (network total dairy intake) 46

Supplemental Table 19: League tables presenting results (mean differences with 95% confidence intervals) of the network meta-analysis on total dairy intake (white fields) as well as results of pairwise meta-analyses (light grey fields) for (a) HDL-C, (b) fasting glucose, (c) glycated hemoglobin (d) energy intake (outcomes without GRADE) 47

Supplemental Table 20: GRADE evaluation of triglycerides (mmol/L) for all comparisons (network dairy product intake) 48

Supplemental Table 21: League tables presenting results (mean differences with 95% confidence intervals) of the network meta-analysis on dairy product intake with a combined control and low dairy

group (white fields) as well as results of pairwise meta-analyses (light grey fields) for (a) HDL-C, (b) fasting glucose, (c) energy intake (outcomes without GRADE). 49

Supplemental Table 22: GRADE evaluation of LDL-C (mmol/L) for all comparisons (network dairy product intake).. 50

Supplemental Table 23: Results (mean differences with 95% confidence intervals) of pairwise meta-analyses using random effects model on mixed dairy products vs. control and kefir vs. milk for glycated hemoglobin (%).. 51

Supplemental Table 24: GRADE evaluation of systolic blood pressure (mmHg) for all comparisons network total dairy intake) 52

Supplemental Table 25: GRADE evaluation of systolic blood pressure (mmHG) for all comparisons (network dairy product intake) 53

Supplemental Table 26: Relative ranking of interventions on total dairy intake (P-scores*)............ 54

Supplemental Table 27: Relative ranking of interventions on intake of dairy products (P-scores*).. 55

Supplemental Figure 1: Summary of the risk of bias assessment with RoB2 for the included RCTs with parallel design (n=18); 58

Supplemental Figure 2: Summary of the risk of bias assessment with RoB2 for the included RCT with cross-over design.. 59

Supplemental Figure 3: Forest plots summarizing mean differences with 95% confidence intervals and network plots for (A) body weight (B), body mass index (C) fat mass (D) LDL-C, (E) HDL-C, (F) triglycerides, (G) fasting glucose, (H) systolic blood pressure and (I) energy intake as estimated from the network meta-analysis on dairy product intake. 64

Supplemental Figure 4: Disconnected network plots for outcomes (A) waist circumference and (B) glycated hemoglobin and interventions on dairy product intake 65

Supplemental Figure 5: Forest plot summarizing mean differences with 95% confidence interval and network plot for energy intake (kcal/d; n studies:14; n participants: 945) as estimated from the network meta-analysis on total dairy intake with a combined control/low dairy group..................................... 66

Supplementary Figure 6: Comparison adjusted funnel plot for (A) body weight, (B) fat mass, (C) waist circumference and (D) energy intake for the network meta-analysis on total dairy intake with a combined control/low dairy group 69

Supplemental Figure 7: Comparison adjusted funnel plot for (A) body weight, (B) fat mass, (C) LDL-C, (D) HDL-C, (E) triglycerides and (F) energy intake for the network meta-analysis on dairy product intake 73

Supplemental Figure 8: Forest plots of sensitivity analyses summarizing mean differences with 95% confidence intervals and network plots for (A) body weight (B), body mass index (C) fat mass (D) waist circumference, (E) LDL-C, (F) HDL-C, (G) triglycerides, (H) fasting glucose, (I) systolic blood pressure and (J) energy intake as estimated from the network meta-analysis on total dairy intake with separate control and low dairy groups. 79

Supplemental Figure 9: Forest plots of sensitivity analyses summarizing mean differences with 95% confidence intervals for (A) body weight, (B) fat mass, (C) waist circumference, (D) LDL-C, (E) HDL-C, (F) triglycerides, (G) systolic blood pressure and (H) energy intake as estimated from the network meta-analysis on total dairy intake with a combined control/low dairy group after excluding studies with high risk of bias 82

Supplemental Figure 10: Forest plots of subgroup analyses (hypocaloric diet [red] and eucaloric/ad libitum diet [black]) summarizing mean differences with 95% confidence intervals for (A) body weight, (B) fat mass, (C) waist circumference, (D) LDL-C, (E) HDL-C, (F) triglycerides, (G) fasting glucose, (H) systolic blood pressure and (I) energy intake as estimated from the network meta-analysis on total dairy intake with a combined control/low dairy group 85

References of included studies... 86

References of excluded studies 88

Supplemental Table 1: Deviations from protocol (PROSPERO; registration number CRD42022303198)

Domain	Deviation
Sensitivity Analysis	In addition to the pre-planned sensitivity analysis regarding risk of bias, we conducted a second sensitivity analysis by separating the group control/low dairy in a control and a low dairy group.
Subgroup Analysis	Planned subgroup analyses, i.e. based on intervention duration, gender, and geographical location were not performed due to insufficient data to perform network meta-analyses.<br>A non-pre-planned subgroup analysis was conducted based on the type of diet (hypocaloric vs. eucaloric/ad libitum) to consider potential effects of a caloric restriction on the outcomes.

Supplemental Table 2: Search strategies for all electronic databases

**Medline via Ovid** (23th September 2022)	
1	exp Dairy Products/
2	(dairy or milk or butter$ or ghee or cheese$ or cream$ or yogurt$ or yoghurt$ or kefir$ or buttermilk or koumiss).ti,kf. or (dairy or milk or butter$ or ghee or cheese$ or cream$ or yogurt$ or yoghurt$ or kefir$ or buttermilk or koumiss).ab. /freq=2
3	1 or 2
4	exp diabetes mellitus, type 2/
5	Prediabetic State/
6	exp insulin resistance/
7	hyperglycemia/
8	dyslipidemias/
9	hyperlipidemias/
10	hypercholesterolemia/
11	exp hypertriglyceridemia/
12	mortality/
13	exp neoplasms/
14	metabolic syndrome/
15	exp hypertension/
16	cardiovascular diseases/
17	heart diseases/
18	vascular diseases/
19	exp Stroke/
20	exp Arteriosclerosis/
21	overweight/
22	obesity/
23	obesity, abdominal/
24	obesity, morbid/
25	body weight changes/
26	weight gain/
27	weight loss/
28	or/4-27
29	((inflamm$ or metabolic$ or cardiometabolic or cardiovascular) adj5 (syndrome$ or disorder$ or outcome$ or biomarker$)).ti,ab,kf.
30	hypertens$.ti,ab,kf.
31	((high$ or increas$ or elevat$ or low$) adj5 blood pressure).ti,ab,kf.
32	(((cardiovascul* or cardiac* or heart or coronary or myocard* or pericard* or vascular or artery or arteries or arterial or vessel or vessels) adj3 (disease* or disorder*))
33	Arteriosclero$.ti,ab,kf.
34	(adipos$ or obese$ or obesit$ or overweight).ti,ab,kf.
35	((body mass index or bmi or fat mass or (body adj2 fat) or body composition$ or anthropometr$) adj5 (change$ or differ$ or reduc$ or low$ or increas$ or gain$ or elevat$)).ti,ab,kf.
36	(stroke or isch?em$ or cerebrovasc$ or apoplexy or ((brain$ or cerebral or lacunar) adj2 infarct$)).ti,ab,kf.
37	(prediabet$ or pre-diabet$).ti,ab,kf.
38	insulin resistan$.ti,ab,kf.
39	(dm2 or t2d or dm type 2 or type 2 diabet* or dm type II or type two diabet* or type II diabet* or dm type II).ti,ab,kf.

40	hyperglycemi$.ti,ab,kf.
41	HbA1c.ti,ab,kf.
42	(dyslipid?emia$ or Hyperlip?emia$ or Hyperlipid?emia$ or Lipide?mia$ or Lipe?mia$).ti,ab,kf.
43	(Hypercholesterolem$ or Hypercholester?emia$ or ((high or increas$ or elevat$ or low$) adj5 cholesterol$)).ti,ab,kf.
44	hypertriglycerid?emia$.ti,ab,kf.
45	mortality.ti,ab,kf.
46	(cancer$ or carcinoma$ or adenocarcin$ or tumor$ or tumour$ or neoplasm$ or neoplastic or neoplasia or malignan$ or metastases or metastasis or metastatic or carcinoid$ or oncol$).ti,ab,kf.
47	or/29-46
48	28 or 47
49	3 and 48
50	(Adolescent/ or Child/ or Infant/ or adolescen*.ti,ab,kf. or child*.ti,ab,kf. or schoolchild*.ti,ab,kf. or infant*.ti,ab,kf. or girl*.ti,ab,kf. or boy*.ti,ab,kf. or teen.ti,ab,kf. or teens.ti,ab,kf. or teenager*.ti,ab,kf. or youth*.ti,ab,kf. or pediatr*.ti,ab,kf. or paediatr*.ti,ab,kf. or puber*.ti,ab,kf.) not (Adult/ or adult*.ti,ab,kf. or man.ti,ab,kf. or men.ti,ab,kf. or woman.ti,ab,kf. or women.ti,ab,kf.)
51	49 not 50
52	randomized controlled trial.pt.
53	controlled clinical trial.pt.
54	randomized.ab.
55	placebo.ab.
56	*Clinical Trials as Topic/
57	randomly.ti,ab.
58	trial.ti.
59	or/52-58
60	exp animals/ not humans.sh.
61	59 not 60
62	51 and 61
**CENTRAL via CRSO (23th September 2022)**	
#1	((dairy or milk or butter* or ghee or cheese* or cream* or yogurt* or yoghurt* or kefir* or buttermilk or koumiss)):TI,AB,KY
#2	MESH DESCRIPTOR Dairy Products EXPLODE ALL TREES
#3	#1 OR #2
#4	MESH DESCRIPTOR diabetes mellitus, type 2 EXPLODE ALL TREES
#5	MESH DESCRIPTOR Prediabetic State
#6	MESH DESCRIPTOR insulin resistance EXPLODE ALL TREES
#7	MESH DESCRIPTOR hyperglycemia
#8	MESH DESCRIPTOR dyslipidemia
#9	MESH DESCRIPTOR hyperlipidemias
#10	MESH DESCRIPTOR hypercholesterolemia
#11	MESH DESCRIPTOR hypertriglyceridemia EXPLODE ALL TREES
#12	MESH DESCRIPTOR mortality
#13	MESH DESCRIPTOR neoplasms EXPLODE ALL TREES
#14	MESH DESCRIPTOR metabolic syndrome
#15	MESH DESCRIPTOR hypertension EXPLODE ALL TREES
#16	MESH DESCRIPTOR cardiovascular diseases
#17	MESH DESCRIPTOR heart diseases

#18	MESH DESCRIPTOR vascular diseases
#19	MESH DESCRIPTOR Stroke EXPLODE ALL TREES
#20	MESH DESCRIPTOR Arteriosclerosis EXPLODE ALL TREES
#21	MESH DESCRIPTOR overweight
#22	MESH DESCRIPTOR obesity
#23	MESH DESCRIPTOR obesity, abdominal
#24	MESH DESCRIPTOR obesity, morbid
#25	MESH DESCRIPTOR body weight changes
#26	MESH DESCRIPTOR weight gain
#27	MESH DESCRIPTOR weight loss
#28	#4 OR #5 OR #6 OR #7 OR #8 OR #9 OR #10 OR #11 OR #12 OR #13 OR #14 OR #15 OR #16 OR #17 OR #18 OR #19 OR #20 OR #21 OR #22 OR #23 OR #24 OR #25 OR #26 OR #27
#29	(inflamm* or metabolic* or cardiometabolic or cardiovascular) adj3 (syndrome* or disorder* or outcome* or biomarker*):TI,AB,KY
#30	hypertens*:TI,AB,KY
#31	((high* or increas* or elevat* or low*) adj3 blood pressure):TI,AB,KY
#32	(((cardiovascul* or cardiac* or heart or coronary or myocard* or pericard* or vascular or artery or arteries or arterial or vessel or vessels) adj3 (disease* or disorder*))):TI,AB,KY
#33	Arteriosclero*:TI,AB,KY
#34	(adipos* or obese* or obesit* or overweight):TI,AB,KY
#35	(((body mass index or bmi or fat mass or (body adj2 fat) or body composition* or anthropometr*) adj3 (change* or differ* or reduc* or low* or increas* or gain* or elevat*))):TI,AB,KY
#36	((stroke or isch?em* or cerebrovasc* or apoplexy or ((brain* or cerebral or lacunar) adj2 infarct*))):TI,AB,KY
#37	((prediabet* or pre-diabet*)):TI,AB,KY
#38	(insulin resistan*):TI,AB,KY
#39	((dm2 or t2d or dm type 2 or type 2 diabet* or dm type II or type two diabet* or type II diabet* or dm type II)):TI,AB,KY
#40	hyperglycemi*:TI,AB,KY
#41	HbA1c:TI,AB,KY
#42	((dyslipid?emia* or Hyperlip?emia* or Hyperlipid?emia* or Lipide?mia* or Lipe?mia*)):TI,AB,KY
#43	((Hypercholesterolem* or Hypercholester?emia* or ((high or increas* or elevat* or low*) adj5 cholesterol*))):TI,AB,KY
#44	hypertriglycerid?emia*:TI,AB,KY
#45	mortality:TI,AB,KY
#46	((cancer* or carcinoma* or adenocarcin* or tumor* or tumour* or neoplasm* or neoplastic or neoplasia or malignan* or metastases or metastasis or metastatic or carcinoid* or oncol*)):TI,AB,KY
#47	#29 OR #30 OR #31 OR #32 OR #33 OR #34 OR #35 OR #36 OR #37 OR #38 OR #39 OR #40 OR #41 OR #42 OR #43 OR #44 OR #45 OR #46
#48	#28 OR #47
#49	#3 AND #48
#50	(MESH DESCRIPTOR age groups EXPLODE ALL TREES)
#51	MESH DESCRIPTOR adult EXPLODE ALL TREES
#52	#50 NOT #51

#53	(adolescen* or child* or schoolchild* or infant* or girl* or boy* or teenor teensor teenager* or youth* or pediatr* or paediatr* or puber*) not (adult* or man or men or woman or women)
#54	#52 or #53
#55	#49 NOT #54
#56	(NCT0* or ACTRN* or ChiCTR* or DRKS* or EUCTR* or eudract* or IRCT* or ISRCTN* or JapicCTI* or JPRN* or NTR0* or NTR1* or NTR2* or NTR3* or NTR4* or NTR5* or NTR6* or NTR7* or NTR8* or NTR9* or SRCTN* or UMIN0*):AU
#57	#55 NOT #56
**Web of Science via Clarivate** (23th September 2022)	
1	TS=(dairy OR milk OR butter OR ghee OR cheese* OR cream OR yogurt* OR yoghurt* OR kefir OR buttermilk OR koumiss)
2	TI=(((inflamm* OR metabolic OR cardiometabolic OR cardiovascular) NEAR/3 (syndrome OR disorder OR outcome OR biomarker))) OR AB=((inflamm* OR metabolic OR cardiometabolic OR cardiovascular) NEAR/3 (syndrome OR disorder OR outcome OR biomarker))
3	TI=(((hypertens*))) OR AB=((hypertens*))
4	TI=((((high* OR increas* OR elevat* OR low*) NEAR/5 "blood pressure"))) OR AB=(((high* OR increas* OR elevat* OR low*) NEAR/5 "blood pressure"))
5	TI=(((cardiovascul* OR cardiac OR heart OR coronary OR myocard* OR pericard* OR vascular OR artery OR arteries OR arterial OR vessel OR vessels) NEAR/3 (disease* OR disorder*))) OR AB=((cardiovascul* OR cardiac OR heart OR coronary OR myocard* OR pericard* OR vascular OR artery OR arteries OR arterial OR vessel OR vessels) NEAR/3 (disease* OR disorder*))
6	TI=(((Arteriosclero*))) OR AB=((Arteriosclero*))
7	TI=(((adipos* OR obese* OR obesit* OR overweight))) OR AB=((adipos* OR obese* OR obesit* OR overweight))
8	TS=((("body mass index" OR bmi OR "fat mass" OR (body NEAR/2 fat) OR "body composition*" OR anthropometr*) NEAR/5 (change* OR differ* OR reduc* OR low* OR increas* OR gain* OR elevat*)))
9	TS=((stroke OR isch$em* OR cerebrovasc* OR apoplexy OR ((brain? OR cerebral OR lacunar) NEAR/2 infarct*)))
10	TI=(((prediabet* OR pre-diabet*))) OR AB=((prediabet* OR pre-diabet*))
11	TI=((("insulin resistan*"))) OR AB=(("insulin resistan*"))
12	TI=(((dm2 OR t2d OR "dm type 2" OR "type 2 diabet*" OR "dm type II" OR "type two diabet*" OR "type II diabet*" OR "dm type II"))) OR AB=((dm2 OR t2d OR "dm type 2" OR "type 2 diabet*" OR "dm type II" OR "type two diabet*" OR "type II diabet*" OR "dm type II"))
13	TI=(((hyperglycemi*))) OR AB=((hyperglycemi*))
14	TI=(((HbA1c))) OR AB=((HbA1c))
15	TI=(((dyslipid$emia* OR Hyperlip$emia* OR Hyperlipid$emia* OR Lipide$mia* OR Lipe$mia* OR Hypercholesterolem* OR Hypercholester$emia*))) OR AB=((dyslipid$emia* OR Hyperlip$emia* OR Hyperlipid$emia* OR Lipide$mia* OR Lipe$mia* OR Hypercholesterolem* OR Hypercholester$emia*))
16	TI=((((high OR increas* OR elevat* OR low*) NEAR/5 cholesterol))) OR AB=(((high OR increas* OR elevat* OR low*) NEAR/5 cholesterol))
17	TI=(((hypertriglycerid?emia*))) OR AB=((hypertriglycerid?emia*))
18	TI=(((mortality))) OR AB=((mortality)) | Exact search
19	TI=(((cancer* OR carcinoma* OR adenocarcin* OR tumor* OR tumour* OR neoplasm* OR neoplastic OR neoplasia OR malignan* OR metastases OR metastasis OR metastatic OR carcinoid* OR oncol*))) OR AB=((cancer* OR carcinoma* OR adenocarcin* OR tumor* OR tumour* OR neoplasm* OR neoplastic OR neoplasia OR malignan* OR metastases OR metastasis OR metastatic OR carcinoid* OR oncol*))

20	#1 AND (#2 OR #3 OR #4 OR #5 OR #6 OR #7 OR #8 OR #9 OR #10 OR #11 OR #12 OR #13 OR #14 OR #15 OR #16 OR #17 OR #18 OR #19)
21	TI=(((Adolescent OR Child OR Infant OR adolescen* OR child* OR schoolchild* OR infant* OR girl* OR boy* OR teen OR teens OR teenager* OR youth* OR pediatr* OR paediatr* OR puber*) NOT (Adult OR adult* OR man OR men OR woman OR women))) OR AB=((Adolescent OR Child OR Infant OR adolescen* OR child* OR schoolchild* OR infant* OR girl* OR boy* OR teen OR teens OR teenager* OR youth* OR pediatr* OR paediatr* OR puber*) NOT (Adult OR adult* OR man OR men OR woman OR women))
22	#20 NOT #21
23	TI=(((random* or "randomi?ed controlled trial" or rct or controlled trial or controlled clinical trial))) OR AB=((random* or "randomi?ed controlled trial" or rct or controlled trial or controlled clinical trial))
24	#22 AND #23

Supplemental Table 3: Additional risk of bias guidance for the included randomized controlled trials

**Domain 1: Risk of bias arising from randomization process**	
**1.1** Was the allocation sequence random?	See guidance.
**1.2** Was the allocation sequence concealed until participants were enrolled and assigned to interventions?	See guidance.
**1.3** Did baseline differences between intervention groups suggest a problem with the randomization process?	Check group sizes.<br>If p-values are given, check for significant differences in baseline characteristics between intervention groups<br>Check for baseline imbalances for key variables such as age, gender, health status, baseline values of outcomes.
**Domain 2: Risk of bias due to deviations from the intended interventions**	
**2.1** Were participants aware of their assigned intervention during the trial?	Blinding is likely not possible due to the nature of the included interventions → Y/PY
**2.2** Were carers and people delivering the interventions aware of participants' assigned intervention during the trial?	Blinding is likely not possible due to the nature of the included interventions → Y/PY
**2.3.** If Y/PY/NI to 2.1 or 2.2: Were there deviations from the intended intervention that arose because of the trial context?<br>**2.4** If Y/PY to 2.3: Were these deviations likely to have affected the outcome?	Check if<br>(a) additional interventions that were introduced were not consistent with trial protocol<br>(b) failure to implement the protocol interventions as intended was evident<br>(c) adherence was assessed and evaluate degree of adherence<br>Judge whether the above mentioned aspects/deviations had an impact on the outcome<br>Assessment of adherence (yes/no/no information); no information→ some concerns<br>Degree of adherence → high risk if large degree of non-adherence<br><u>Check if</u><br>- Percentage value is given (>80% low risk)<br>- Mean number of servings is given (should be close to that described in the intervention protocol)<br>- When high vs. low dairy was investigated, calcium intake could be an indicator of compliance<br>- Limitations are reported.
**2.5** If Y/PY/NI to 2.4: Were these deviations from intended intervention balanced between groups?	See guidance
**2.6** Was an appropriate analysis used to estimate the effect of assignment to intervention?	Check if ITT or modified ITT was used → Y/PY
**2.7** If N/PN/NI to 2.6: Was there potential for a	See guidance

substantial impact (on the result) of the failure to analyse participants in the group to which they were randomized?	
**Domain 3: Risk of bias due to missing outcome data**	
**3.1** Were data for this outcome available for all, or nearly all, participants randomized?	Note that imputed data should be regarded as missing data, and not considered as 'outcome data' in the context of this question.<br><br>Cut-off: ≥20% missing data → N/PN<br><br>Low risk: <20% + valid reasons<br>Some concerns: <20% without valid reasons<br>However, if valid imputation techniques mentioned →low RoB
**3.2** If N/PN/NI to 3.1: Is there evidence that the result was not biased by missing outcome data?	Check if<br>(a) (multiple) imputation was used<br>(b) Sensitivity analysis were conducted<br>(c) Reasons were given
**3.3** If N/PN to 3.2: Could missingness in the outcome depend on its true value?<br><br>**3.4** If Y/PY/NI to 3.3: Is it likely that missingness in the outcome depended on its true value?	High risk: > 20%<br>However, if:<br>- valid imputation techniques mentioned →low RoB<br>- no imputation techniques are used, but valid reasons are mentioned for both groups and are (nearly) equally distributed across groups, we will not assume high RoB
**Domain 4: Risk of bias in measurement of the outcome**	
**4.1** Was the method of measuring the outcome inappropriate?	For anthropometric measures, check if a standardized protocol was used<br>For blood glucose, verify that measurement was not done with a portable tool by participants.<br>For blood pressure, check if standardized protocol was used and measurement was performed by the researcher<br>For energy intake, check the used method (validated tool)
**4.2** Could measurement or ascertainment of the outcome have differed between intervention groups?	Check if outcome measurement differed between groups<br>If Y/PY → High Risk of Bias
**4.3** If N/PN/NI to 4.1 and 4.2: Were outcome assessors aware of the intervention received by study participants?	If N/PN → Low Risk
**4.4** If Y/PY/NI to 4.3: Could assessment of the outcome have been influenced by knowledge of intervention received?	See guidance<br>Self-reported outcomes (energy intake) → Y/PY
**4.5** If Y/PY/NI to 4.4: Is it likely that assessment of the outcome was influenced by knowledge of intervention received?	See guidance

Domain 5: Risk of bias in selection of the reported result	
**5.** Risk of bias in selection of the reported result	Judge “low risk” if protocol is present and there's no evidence for differences between protocol and report.<br>Rate 5.1 as “no information” if registry entry is available but o information about the analysis plan exists<br>Rate 5.2 and 5.3 as “no information” if no study protocol/registration is available and no deviations are reported in the manuscript.

Abbreviations: ITT intention-to-treat analysis, N no, NI no information, RoB risk of bias, PY partial yes, Y yes

Supplemental Table 4: Reports of the 19 included Randomized Controlled Trials

Study ID	Reports of included RCTs
Bellikci-Koyu 2019 (43)	Bellikci-Koyu E, Sarer-Yurekli BP, Akyon Y, Aydin-Kose F, Karagozlu C, Ozgen AG, et al. Effects of Regular Kefir Consumption on Gut Microbiota in Patients with Metabolic Syndrome: A Parallel-Group, Randomized, Controlled Study. Nutrients. 2019;11(9): 2089.<br><br>Bellikci-Koyu E, Sarer-Yurekli BP, Karagozlu C, Aydin-Kose F, Ozgen AG, Buyuktuncer Z. Probiotic kefir consumption improves serum apolipoprotein A1 levels in metabolic syndrome patients: a randomized controlled clinical trial. Nutr Res. 2022;102:59-70.
Bendtsen 2018 (44)	Bendtsen LQ, Blædel T, Holm JB, Lorenzen JK, Mark AB, Kiilerich P, et al. High intake of dairy during energy restriction does not affect energy balance or the intestinal microflora compared with low dairy intake in overweight individuals in a randomized controlled trial. Applied physiology, nutrition, and metabolism = Physiologie appliquee, nutrition et metabolisme. 2018;43(1):1-10.<br><br>Zheng H, Lorenzen JK, Astrup A, Larsen LH, Yde CC, Clausen MR, et al. Metabolic Effects of a 24-Week Energy-Restricted Intervention Combined with Low or High Dairy Intake in Overweight Women: An NMR-Based Metabolomics Investigation. Nutrients. 2016;8(3):108.
Campbell 1999 (45)	Campbell CG, Chew BP, Luedecke LO, Shultz TD. Yogurt consumption does not enhance immune function in healthy premenopausal women. Nutrition and Cancer-an International Journal. 2000;37(1):27-35.<br><br>Campbell CG, Luedecke LO, Shultz TD. Yogurt consumption and estrogen metabolism in healthy premenopausal women. Nutrition Research. 1999;19(4):531-43.
Chen 2019 (46)	Chen Y, Feng RN, Yang X, Dai JX, Huang M, Ji XN, et al. Yogurt improves insulin resistance and liver fat in obese women with nonalcoholic fatty liver disease and metabolic syndrome: a randomized controlled trial. American Journal of Clinical Nutrition. 2019;109(6):1611-9.
Engel 2018 (47)	Maersk M, Belza A, Stodkilde-Jorgensen H, Ringgaard S, Chabanova E, Thomsen H, et al. Sucrose-sweetened beverages increase fat storage in the liver, muscle, and visceral fat depot: a 6-mo randomized intervention study. American Journal of Clinical Nutrition. 2012;95(2):283-9.<br><br>Engel S, Tholstrup T, Bruun JM, Astrup A, Richelsen B, Raben A. Effect of high milk and sugar-sweetened and non-caloric soft drink intake on insulin sensitivity after 6 months in overweight and obese adults: a randomized controlled trial. European Journal of Clinical Nutrition. 2018;72(3):358-66.<br><br>Engel S, Tholstrup T, Bruun JM, Astrup A, Richelsen B, Raben A. Correction: Effect of high milk and sugar-sweetened and noncaloric soft drink intake on insulin sensitivity after 6 months in overweight and obese adults: a randomized controlled trial. Eur J Clin Nutr. 2020;74(1):210-3.
Gunther 2005 (48)	Gunther CW, Legowski PA, Lyle RM, McCabe GP, Eagan MS, Peacock M, et al. Dairy products do not lead to alterations in body weight or fat mass in young women in a 1-y intervention. American Journal of Clinical Nutrition. 2005;81(4):751-6.
Harvey-Berino 2005 (49)	Harvey-Berino J, Gold BC, Lauber R, Starinski A. The impact of calcium and dairy product consumption on weight loss. Obesity Research. 2005;13(10):1720-6.
Raziani 2016 (50)	Raziani F, Tholstrup T, Kristensen MD, Svanegaard ML, Ritz C, Astrup A, et al. High intake of regular-fat cheese compared with reduced-fat cheese does not affect

	LDL cholesterol or risk markers of the metabolic syndrome: a randomized controlled trial. American Journal of Clinical Nutrition. 2016;104(4):973-81<br><br>Raziani F, Ebrahimi P, Engelsen SB, Astrup A, Raben A, Tholstrup T. Consumption of regular-fat vs reduced-fat cheese reveals gender-specific changes in LDL particle size - a randomized controlled trial. Nutrition & Metabolism. 2018;15(1) (no pagination).
Rideout 2013 (51)	Rideout TC, Marinangeli CP, Martin H, Browne RW, Rempel CB. Consumption of low-fat dairy foods for 6 months improves insulin resistance without adversely affecting lipids or bodyweight in healthy adults: a randomized free-living cross-over study. Nutrition Journal. 2013;12:56.
Schmidt 2021 (52)	Schmidt KA, Cromer G, Burhans MS, Kuzma JN, Hagman DK, Fernando I, et al. The impact of diets rich in low-fat or full-fat dairy on glucose tolerance and its determinants: a randomized controlled trial. American Journal of Clinical Nutrition. 2021;113(3):534-47<br><br>Schmidt KA, Cromer G, Burhans MS, Kuzma JN, Hagman DK, Fernando I, et al. Impact of low-fat and full-fat dairy foods on fasting lipid profile and blood pressure: exploratory endpoints of a randomized controlled trial. American Journal of Clinical Nutrition. 2021;114(3):882-92.
Tanaka 2014 (53)	Tanaka S, Uenishi K, Ishida H, Takami Y, Hosoi T, Kadowaki T, et al. A Randomized Intervention Trial of 24-wk Dairy Consumption on Waist Circumference, Blood Pressure, and Fasting Blood Sugar and Lipids in Japanese Men with Metabolic Syndrome. Journal of Nutritional Science and Vitaminology. 2014;60(5):305-12
Thomas 2010 (54)	Thomas DT, Wideman L, Lovelady CA. Effects of Calcium and Resistance Exercise on Body Composition in Overweight Premenopausal Women. Journal of the American College of Nutrition. 2010;29(6):604-11.
Thompson 2005 (55)	Thompson WG, Holdman NR, Janzow DJ, Slezak JM, Morris KL, Zemel MB. Effect of energy-reduced diets high in dairy products and fiber on weight loss in obese adults. Obesity Research. 2005;13(8):1344-53.
Van Loan 2011 (56)	Van Loan MD, Keim NL, Adams SH, Souza E, Woodhouse LR, Thomas A, et al. Dairy Foods in a Moderate Energy Restricted Diet Do Not Enhance Central Fat,Weight, and Intra-Abdominal Adipose Tissue Losses nor Reduce Adipocyte Size or Inflammatory Markers in Overweight and Obese Adults: A Controlled Feeding Study. Journal of Obesity. 2011;2011:989657<br><br>Krishnan S, Adams SH, Witbracht MG, Woodhouse LR, Piccolo BD, Thomas AP, et al. Weight Loss, but Not Dairy Composition of Diet, Moderately Affects Satiety and Postprandial Gut Hormone Patterns in Adults. Journal of Nutrition. 2021;151(1):245-54.<br><br>Labouesse MA, Gertz ER, Piccolo BD, Souza EC, Schuster GU, Witbracht MG, et al. Associations among endocrine, inflammatory, and bone markers, body composition and weight loss induced bone loss. Bone. 2014;64:138-46.<br><br>Witbracht MG, Van Loan M, Adams SH, Keim NL, Laugero KD. Dairy food consumption and meal-induced cortisol response interacted to influence weight loss in overweight women undergoing a 12-week, meal-controlled, weight loss intervention. Journal of Nutrition. 2013;143(1):46-52.
Wennersberg 2009 (57)	Wennersberg MH, Smedman A, Turpeinen AM, Retterstol K, Tengblad S, Lipre E, et al. Dairy products and metabolic effects in overweight men and women: results from a 6-mo intervention study. American Journal of Clinical Nutrition. 2009;90(4):960-8.

Zemel 2004 (58)	Zemel MB, Thompson W, Milstead A, Morris K, Campbell P. Calcium and dairy acceleration of weight and fat loss during energy restriction in obese adults. Obesity Research. 2004;12(4):582-90.<br><br>Sasraku JND. Increasing dietary calcium in the face of caloric restrictions in humans. Annals of Nutrition & Metabolism. 2013;63:1400
Zemel 2005 (59)	Zemel MB, Richards J, Milstead A, Campbell P. Effects of calcium and dairy on body composition and weight loss in African-American adults. Obesity Research. 2005;13(7):1218-25.
Zemel 2009 (60)	Zemel MB, Teegarden D, Van Loan M, Schoeller DA, Matkovic V, Lyle RM, et al. Dairy-Rich Diets Augment Fat Loss on an Energy-Restricted Diet: A Multicenter Trial. Nutrients. 2009;1(1):83-100.

Supplemental Table 5: Reasons for excluding studies at full-text screening (n=48)

Reference	Reason for exclusion
(79, 80)	Wrong study design
(81)	Wrong patient population
(82-90)	Wrong intervention
(91, 92)	Wrong dose
(93-100) (101)	Wrong comparator
(102-107)	Energy Intake not available
(108-113) (114)	Energy intake differed
(115-124)	Duplicate report
(125, 126)	Co-intervention differed

Supplemental Table 6: Study and Participants' characteristics of included trials (n=19)

**Study Author (Country, Year)**	**RCT Design**	**Duration I/F** (weeks)	**Sample size Total** (IA1/IA2/IA3)	**Female** (%)	**Mean Age** (years)	**Mean BMI** (kg/m²)	**Health Status**	**Outcomes**
Bellikci-Koyu (Turkey, 2019) (43)	parallel	12/0	78 (39/39)	71	IA1: 50.5<br>IA2: 49.1	IA1: 32.9<br>IA2: 32.3	MetS	BW, BMI, WC, FM(%), LDL-C, HDL-C, TG, FG, HbA1c, SBP, Energy Intake
Bendtsen (Denmark, 2018) (44)	parallel	24/0	80 (40/40)	IA1: 85<br>IA2: 88	IA1: 45<br>IA2: 42	IA1: 30.8<br>IA2: 31.5	overweight/obesity	BW, BMI, WC, FM(%), LDL-C, HDL-C, TG, FG, SBP, Energy Intake
Campbell (Washington State/USA, 1999) (45)	parallel	12.9/0	25 (12/13)	100	24	IA1: 21.9<br>IA2: 22.0	healthy premenopausal women, no history of menstrual cycle irregularities or gynecological disorders	BW, Energy Intake
Chen (China, 2019) (46)	parallel	24/0	100 (50/50)	100	IA1: 51.2<br>IA2: 48.9	IA1: 31.8<br>IA2: 32.2	obese (cut offs for Asian population)<br>NAFLD<br>MetS	BMI, WC, FM, LDL-C, HDL-C, TG, FG, SBP, Energy Intake
Engel (Denmark, 2018) (47)	parallel	25.8/0	35 (20/15)	66.7	IA1: 39.0<br>IA2: 37.7	IA1: 31.5<br>IA2: 31.4	healthy overweight/obesity	BW, BMI, FM, LDL-C, HDL-C, TG, FG, SBP, Energy Intake
Gunther (Indiana/USA, 2005) (48)	parallel	51.6/0	155 (42/45/48)[+]	100	IA1: 20.1<br>IA2: 20.2<br>IA3: 20.1	IA1: 22.1<br>IA2: 23.3<br>IA3: 22.4	healthy normal-weight	BW, BMI, FM, Energy Intake
Harvey-Berino (Vermont/USA, 2005) (49)	parallel	51.6/0	54 (25/29)	IA1: 89.7<br>IA2: 92.0	IA1: 45.1<br>IA2: 45.2	IA1: 29.8<br>IA2: 30.2	overweight/obesity	BW, FM, Energy Intake
Raziani (Denmark, 2016) (50)	parallel	12/0	109 (56/53)	IA1: 64<br>IA2: 69	IA1: 53.8<br>IA2: 50.6	IA1: 29.3<br>IA2: 28.1	Increased WC and 1 additional MetS risk factor	BW, BMI, WC, FM, LDL-C, HDL-C, TG, FG, SBP, Energy Intake

Rideout (Canada, 2013) (51)*	cross-over	25.8/0	39 (19/20)	78.3	53	31.9	healthy	BW, WC, FM(%), LDL-C, HDL-C, TG, FG, SBP, Energy Intake
Schmidt (Washington / USA, 2021) (52)	parallel	12/0	72 (24/24/24)	IA1: 45.8<br>IA2: 41.7<br>IA3: 41.7	IA1: 56<br>IA2: 64<br>IA3: 63	IA1: 33.2<br>IA2: 30.9<br>IA3: 32.0	weight-stable MetS	BW, WC, FM, LDL-C, HDL-C, TG, FG, HbA1c, SBP, Energy Intake
Tanaka (Japan, 2014) (53)	parallel	24/0	213 (107/106)	IA1: 0<br>IA2: 0	IA1: 41.7<br>IA2: 41.7	IA1: 26.8<br>IA2: 27.2	>50% obesity low prevalence of hypertension, type 2 diabetes, and dyslipidemia	BW, WC, FM(%), LDL-C, HDL-C, TG, FG, HbA1c, SBP, Energy Intake
Thomas (North Carolina / USA, 2010) (54)	parallel	16/0	35 (17/18)	IA1: 100<br>IA2: 100	IA1: 37.1<br>IA2: 36.4	IA1: 28.9<br>IA2: 29.3	overweight sedentary	BW, WC, FM, Energy Intake
Thompson (Minesota / USA, 2005) (55)	parallel	48/0	60 (29/31)	IA1: 86.2<br>IA2: 86.7	IA1: 42.0<br>IA2: 41.2	IA1: 35.0<br>IA2: 35.0	obesity	BW, WC, FM, LDL-C, HDL-C, TG, FG, Energy Intake
Van Loan (USA, 2011) (56)	parallel	12/0	78 (40/38)	IA1: 76.5<br>IA2: 74.2	32.5<br>IA1: 31.9<br>IA2: 32.8	IA1: 33.8<br>IA2: 32.5	healthy overweight/obesity weight stable	BW, BMI, WC, FM, LDL-C, HDL-C, TG, FG
Wennersberg (Finland, Norway, Sweden, 2009) (57)	parallel	26/0	121 (60/61)	66.1	women: 56.7<br>men: 51.2	IA1: 30.0<br>IA2: 30.1	apparently healthy men postmenopausal women overweight/obesity MetS	BW, BMI, WC, FM, LDL-C, HDL-C, TG, FG, HbA1c, SBP, Energy Intake
Zemel (Tennesse / USA, 2004) (58)	parallel	24/0	28 (14/14)	82.9	46	35.0	healthy obesity	BW, WC, FM, LDL-C, HDL-C, TG, FG, SBP
Zemel (Tennesse / USA, 2005) (phase 1) (59)	parallel	24/0	34 (17/17)	IA1: 52.9<br>IA2: 82.4	IA1: 41.3<br>IA2: 42.5	IA1: 34.9<br>IA2: 34.1	healthy obesity	BW, WC, FM, LDL-C, HDL-C, TG, SBP, Energy Intake

Zemel (Tennesse / USA, 2005) (59) (phase 2)	parallel	24/0	39 (12/17)	IA1: 91.7<br>IA2: 82.4	IA1: 41.7<br>IA2: 41.7	IA1: 35.4<br>IA2: 35.6	healthy obesity	BW, WC, FM, LDL-C, HDL-C, TG, SBP, Energy Intake
Zemel (Indiana, Tennesse, California, Ohio / USA, 2009) (60)	parallel	12/0	70 (38/32)	IA1: 78.9<br>IA2: 75.0	IA1: 25.35<br>IA2: 25.55	IA1: 29.4<br>IA2: 28.8	healthy overweight/ mildly obesity	BW, WC, FM, SBP, Energy intake

Abbreviations: BMI Body Mass Index, BW Body Weight, F Follow-Up, FG Fasting Glucose, FM Fat Mass, HbA1c Glycated Hemoglobin, HDL-C High-Density Lipoprotein Cholesterol, I Intervention, IA Intervention Arm, LDL-C Low-Density Lipoprotein Cholesterol, MetS Metabolic Syndrome, NAFLD Non-Alcoholic Fatty Liver Disease, RCT Randomized Controlled Trial, SBP Systolic Blood Pressure, TG Triglycerides, WC Waist Circumference;

$^{+}$ Numbers in brackets refer to completers *Cross-over study not considered in network meta-analysis as no data for the first intervention period was available.

Supplemental Table 7: Reporting of study funding and conflict of interest statements in included trials (n=19)

**Author, Year**	**Study Funding**	**Conflict of Interest Statement**
Bellikci-Koyu, 2019 (43)	"This research was funded by the Turkish Council of Higher Education."	None
Bendtsen, 2018 (44)	"The study was supported by the Danish Council for Strategic Research and the Danish Dairy Research foundation. The sponsors had no role in the design, analysis or writing of this article."	“A.A. is currently a member of an advisory board for the Global Dairy Platform, USA and a member of the Steering Committee of the Arla Foods, University of Copenhagen, Aarhus University Dairy Health and Nutrition Excellence Center, Denmark. A.A., L.Q.B., T.B., J.K.L., A.B.M., K.K., and L.H.L. have received funding for research from Arla Foods A/S, Denmark, and the Danish Dairy Research Foundation. J.B.H. and P.K. declare no conflicts of interest.”
Campbell, 1999 (45)	"This study was supported in part by funds provided by the Washington State Dairy Products Commission."	NR
Chen, 2019 (46)	"The study is supported by the National Natural Science Foundation of China (grants 81872616 and 81573133), Natural Science Foundation of Heilongjiang Province (grant H2016018), and Heilongjiang Provincial Postdoctoral Commission Science Foundation (grant LBH-Q17089) to RF."	None
Engel, 2018 (47)	“Supported by grants from the Danish Council for Strategic Research, The Food Study Group/Danish Ministry of Food, Agriculture and Fisheries, Novo Nordic Foundation, and Clinical Institute at Aarhus University, Denmark. The semiskim milk was donated by the Danish Dairy Company, Arla Foods, but without any influence on the design, interpretation, or conclusions of the study.”	None
Gunther, 2005 (48)	"Supported by the National Dairy Council"	None
Harvey-Berino, 2005 (49)	"This study was supported by the National Dairy Council, by the Northeast Dairy Foods Research Center, by University of Vermont GCRC Grant M01-RR109, and by Cabot Cheese."	None
Raziani, 2016 (50)	“The study was 50% financed by the Danish Dairy Research Foundation, Danish Agriculture and Food Council (Denmark), and 50% by the National Dairy Council (United States), the Dairy Farmers of Canada (Canada), Centre National Interprofessionel de l’Economie Laitière (France), Dairy Australia (Australia), and Nederlandse Zuivel Organisatie (Netherlands).”	“AA has received research grants from Arla Foods AMBA, Denmark; The Danish Dairy Research Foundation, Denmark; Global Dairy Platform, USA; and the Danish Agriculture and Food Foundation, Denmark. TT has received research grants from Arla Foods AMBA, Denmark; The Danish Dairy Research Foundation;

		and the Dairy Institute, Rosemont, IL. AR has received research funding from the Dairy Research Industry, Rosemont, IL, and The Danish Agriculture and Food Council, Denmark.”
Rideout, 2013 (51)	"This study was supported in part by a Science & Technology International Collaboration (STIC) grant from the Manitoba Department of Innovation, Energy and Mines."	None
Schmidt, 2021 (52)	“Supported by contract number 2395 by National Dairy Council, Dairy Farmers of Canada, Dutch Dairy Association (Nederlandse Zuivel Organisatie), Dairy Australia, and the French Dairy Interbranch Organization (CNIEL); NIH grant P30 DK017047 (University of Washington Diabetes Research Center); NIH grant P30 CA015704 (Fred Hutchinson Cancer Research Center Cancer Center Support Grant). KAS was supported in part by grant T32 CA094880 from the NIH. MSB was supported in part by grants R25CA094880, T32DK007247, and T32HL007028 from the NIH. KMU is supported by the Department of Veterans Affairs. This study was initiated by the principal investigator (MK). The dairy-related funding organizations suggested changes to details of the study design prior to the conduct of the study, some of which were implemented. Otherwise, the funding organizations had no impact on the design or conduct of the trial or the analysis and interpretation of study data.”<br><br>“This dissertation project of KAS was funded by an international consortium of dairy organizations, including the US National Dairy Council, Dairy Farmers of Canada, the Dutch Dairy Association (Nederlandse Zuivel Organisatie), Dairy Australia, and the French Dairy Interbranch Organization (CNIEL). MK has received honoraria and reimbursements for travel as well as a research grant for this project from several dairy organizations, including the US National Dairy Council, Dairy Farmers of Canada,Nederlandse Zuivel Organisatie, Dairy Australia, and CNIEL. JK has received honoraria and reimbursements for travel as well as research grants from the Vermont Dairy Promotion Council and the National Dairy Council/Dairy Management Inc.”	“MK is a member of the AJCN Editorial Board. The other authors report no conflicts of interest.”
Tanaka, 2014 (53)	“The Japan Dairy Association provided financial support for this study. The authors received funding and honoraria for participation in meetings for	“Y.T. is an employee of Meiji Co., Ltd., and is an expert on loan to the Japan Dairy Association, where he works as the Executive Director.”

	this study from the Japan Dairy Association. S.T., T.H., and T.K. received lecture fees from the Japan Dairy Association."	
Thomas, 2010 (54)	"This study was partially supported by a grant from the Department of Women and Gender Studies at the University of North Carolina Greensboro."	NR
Thompson, 2005 (55)	"This study was funded by the National Dairy Council. Additional support was provided by Grant M01RR00585 to the Mayo General Clinical Research Center and by the Division of Preventive and Occupational Medicine. The funding organization played no role in collecting or analyzing data, preparing the manuscript, or deciding to submit for publication."	"N.R.H. is currently employed by General Mills, which makes yogurt; The National Dairy Council has supported a number of studies by M.B.Z., and he has served on speaker panels for the National Dairy Council."
Van Loan, 2011 (56)	"Major funding for this project was provided by the National Dairy Council administered by the Dairy Research Institute and the Dairy Council of California. Additional support was provided by the USDA, ARSProjects 5306-51530-006-00D and 5306-51530-016-00D, the Clinical and Translational Science Center of the University of California, Davis, and grant no. UL1 RR024146 from the National Center for Research Resources (NCRR)."	None
Wennersberg, 2009 (57)	"Supported by the Finnish Ministry of Agriculture and Forestry; the National Research Council of Norway; the Information Office for Milk Products, Norway; the Swedish Farmer's Foundation for Agricultural Research; and the Swedish Dairy Association."	"None of the authors reported a conflict of interest. None of the authors had any financial or personal relationships with the companies or organizations supporting the study at the time the research was done."
Zemel, 2004 (58)	"This research was supported by the National Dairy Council."	NR
Zemel, 2005 (phase 1) (59)	"This research was supported by The National Dairy Council."	NR
Zemel, 2005 (phase 2) (59)	"This research was supported by The National Dairy Council."	NR
Zemel, 2009 (60)	"This research was supported by a grant from the National Dairy Council (USA)."	NR

Abbreviations: NR Not Reported

Supplemental Table 8: Intervention characteristics of included trials (n=19)

**Author, year**	**Interventions** Arm 1	Arm 2	Arm 3	**Type of diet**	**Co-Interventions in all groups**	**Adherence to intervention (Assessment, Degree)**
Bellikci-Koyu, 2019 (43)	**Unfermented Full-fat Milk**<br>(180ml/d, 3.5% fat)<br>Node(s):<br>1. Milk	**Kefir**<br>(180ml/d, based on 3.5% fat milk)<br>Node(s):<br>2. Kefir	-	Eucaloric	Maintain habitual diet and physical activity	Assessment: Interviewing participants; Reviewing records of consumption in each visit; Non-compliance: <80% of the scheduled serving during the study period<br>Degree: No change in energy intake during intervention, and no difference between groups (P=0.75); No information about intake on dairy products
Bendtsen, 2018 (44)	**Low Dairy**<br>(Ca <600 mg/d and 0–1 dairy products/d); Lists of dairy products with instruction on amount of products allowed eating per day based on the calcium content of products.<br>Node(s):	**High Dairy**<br>(Ca 1500 mg/d with 1200 mg from dairy products, 4-5 dairy products/d);<br>Lists of dairy products with instruction on amount of products allowed eating per day based on the calcium content of products;<br>Instruction to distribute dairy intake throughout the day<br>Node(s):	-	Hypocaloric	Caloric restriction (-500 kcal (2100 kJ) compared with estimated energy requirement; 30E% F, 52E% CHO, 18E% P);<br>Dietary counselling at least every 4 wks	Assessment: 7-day dietary records at wk12 and 24; Participation at counselling visits (7x individual, 1x group)<br>Degree: Significantly reduced energy intake at wk24 without group differences (P=0.95); Ca intake increased in IA2 and significantly decreased in IA1 at wk 24, compared to baseline

	1. Control/ Low Dairy<br>2. Control	1. High Dairy<br>2. Mixed Dairy Products				
Campbell, 1999 (45)	**Control**<br>refrain from all yogurt products<br><br>Node(s):<br>1. Control/ Low Dairy<br>2. Control	**Yogurt**<br>(two cups (454g) of low-fat, vanilla (2,092kJ, 90g CHO, 8g F, 18g P) or plain (1,422kJ, 36g CHO, 9g F, 26g P), non-pasteurized yogurt per day; containing commercially produced yogurt culture strain)<br>Node(s):<br>1. Low Fat, High Dairy<br>2. Yogurt	**-**	Eucaloric	**-**	Assessment: 3-day dietary record<br>Degree:<br>Mean energy intake (12wk, P>0.05):<br>IA1: 2040±126 kcal/d<br>IA2: 2084±144 kcal/d<br>Non-significant increase in Ca intake in both groups, without group differences
Chen, 2019 (46)	**Milk**<br>(220 g/d, whole-fat); instructed to drink before breakfast<br><br>Node(s):<br>1. Milk	**Yogurt**<br>(220 g/d, whole-fat liquid);<br>Instructed to drink before breakfast<br>Node(s):<br>1. Yogurt	**-**	Eucaloric	**-**	Assessment: Scheduled interviews; Counting empty bottles returned every 4wk, FFQ<br>Degree:<br>Mean energy intake (24wk, P<0.05, but all analyses were ANCOVA adjusted):<br>IA1: 2668±883 kcal/d<br>IA2: 2475±926 kcal/d
Engel, 2018 (47)	**Still Mineral Water**<br>(Aqua d'or) 1L/d<br><br>Node(s):	**Semi-skim Milk**<br>(1L/d; CHO 4,7g, P 3,4g F 1,5g per 100ml, E 1900 kJ/d)<br>Node(s):	**-**	Eucaloric	-	Assessment: Drinks handed out 2–3/mo; Counting empty bottles or cartons every 3–4wk; 7-day weighed records

	1. Control/ Low Dairy<br>2. Control	1. Low Fat, High Dairy<br>2. Milk				<u>Degree:</u><br>Mean energy intake:<br>(26wk, P=0.14)<br>IA1: 2542±193 kcal/d<br>IA2: 2855±212 kcal/d
Gunther, 2005 (48)	**Control**<br>Instruction to maintain current dietary consumption<br><u>Node(s):</u><br>1. Control/ Low Dairy<br>2. Control	**Medium Dairy**<br>(1000–1100 mg Ca/d from dairy);<br>Dietary counselling, and maintain isocaloric intake;<br>Emphasis on non-fat and low-fat milk; Lists of substitutions<br><u>Node(s):</u><br>1. Low Fat, High Dairy<br>2. Mixed Dairy Products	**High Dairy**<br>(1300–1400 mg Ca/d from dairy)<br>Dietary counselling and maintain isocaloric intake;<br>Emphasis on non-fat and low-fat milk; Lists of substitutions<br><u>Node(s):</u><br>1. Low Fat, High Dairy<br>2. Mixed Dairy Products	Eucaloric	-	<u>Assessment:</u><br>IA1: 3-mo food records<br>IA2+IA3: Daily records of type and number of servings of dairy foods added and the corresponding foods subtracted; Checking of logs by a nutritionist; Retraining of participants in case of discrepancies;<br>All: Compliance guidelines: 1) maintain a mean daily energy intake ≤2200 kcal/d, 2) daily calcium intake (IA1 no increase >200 mg/d from baseline; IA2+IA3 increase >200 mg/d)<br><u>Degree:</u><br>Mean energy intake (52wk, P>0.05):<br>IA1: 1558±383 kcal/d<br>IA2: 1671±345 kcal/d<br>IA3: 1606±317 kcal/d<br>Discussion: "Slight difference in energy intake between the control and intervention groups represented a lack

						of appropriate substitution by the intervention groups”
Harvey-Berino, 2005 (49)	**Low Dairy**<br>approximately 1 serving of dairy/d (calcium intake goal of 400 to 500 mg/d); Receiving food in the form of fruits, vegetables, or high-fiber grains; Prescribed menus, grocery lists, recipes<br><br>Node(s):<br>1. Control/ Low Dairy<br>2. Control	**High Dairy**<br>3-4 servings of dairy products/d (milk, yogurt, and cheese; dairy calcium intake goal of 1200 to 1400 mg/d); Provision of 2 servings of dairy products/d in the form of yogurt and cheese; Prescribed menus, grocery lists, recipes<br>Node(s):<br>1. High Dairy<br>2. Mixed Dairy Products	-	Hypocaloric	Behavioral weight loss program (-500 kcal/d, 10-15% P, 55-65% CHO, 30% F, 25 g fiber), weekly group session for first 24 weeks, afterwards biweekly.<br>"Graded goals for programmed activity (i.e., walking) were used throughout the program, and participants were encouraged to expend at least 1000 calories/wk in physical activity"	Assessment: Recording of any dietary deviations from prescribed menus; Recording of dietary intake and amount of energy expended in prescribed physical activity daily; Weekly review of self-monitoring Logs; Advice on strategies for adhering to the dietary and exercise plan.<br>Degree: High Dairy group reported consuming between 2 (at 12mo) and 3 (at 3 and 6 mo) more dairy servings/d than participants in the Low Dairy group; No significant differences in distribution of macronutrients by condition over time Mean energy intake (52wk, P>0.05):<br>IA1: 1432±133 kcal/d<br>IA2: 1646±473 kcal/d
Raziani, 2016 (50)	**Regular Cheese**<br>Regular-fat Danbo (40 g 25% fat) and	**Reduced-Fat Cheese**<br>Reduced-fat Danbo (40g, 13% fat) and	-	Eucaloric	Provision of 250 mL skimmed milk/d (0.1%	Assessment: Percentage of test food consumed compared with test food

	cheddar (40g, 32% fat);<br>Guidance how to substitute the cheese for food items from their habitual diets<br>Node(s):<br>1. High Dairy	cheddar (40g, 16% fat);<br>Guidance how to substitute the cheese for food items from their habitual diets<br>Node(s):<br>2. Low Fat, High Dairy			fat) throughout the intervention;<br>No consumption of any other dairy products	handed out, 3-d weighted dietary record<br>Degree:<br>IA1: 98.9%±0.3%<br>IA2: 98.8%± 0.4%<br>Mean energy intake (during 12wk, P=0.81):<br>IA1: 2174±87 kcal/day<br>IA2: 2168±103 kcal/day
Rideout, 2013 (51)	**Low Dairy**<br>no more than 2 servings low fat dairy per day	**High Dairy**<br>4 servings of low fat dairy per day (yogurt, milk);<br>to incorporate dairy products (regularly provided (every 2 wks)) by substitution so as not to increase their normal energy intake	**-**	Eucaloric	Instruction to maintain normal diet and level of physical activity for the duration of the study	Assessment: Provision of a logbook; Record of the number of dairy servings consumed each day, 3-day food record<br>Degree:<br>IA2: Reason for dropping out: inability to consume the required daily amount of dairy (n=2);<br>Mean energy intake (during intervention) P>0.05):<br>IA1: 2396±430 kcal/d<br>IA2: 2268±502 kcal/d<br>Discussion: "… although volunteers were given log books to record their daily dairy intake, these records were not reviewed by the study staff until the end of the study, at which point it was determined that they were incomplete. Therefore, the lack of a

						compliance evaluation and actual dairy intakes are a major limitation of the current study."
Schmidt, 2021 (52)	**Low Dairy**<br>Limiting dairy intake to ≤3 servings/wk of non-fat milk<br><br>Node(s):<br>1. Control/ Low Dairy<br>2. Control	**Low-fat Dairy**<br>3.3 servings of dairy/d in form of non-fat milk, yogurt and low-fat cheese;<br>Instruction not to consume any dairy foods, defined as any food item or mixed dish containing a significant amount of dairy, other than those provided<br><br>Node(s):<br>1. Low Fat, High Dairy<br>2. Mixed Dairy Products	**Full-fat Dairy**<br>3.3 servings of dairy/d in the form of whole milk (3.25% fat), full-fat yogurt (3.1% fat), and full-fat cheese;<br>Instruction not to consume any dairy foods, defined as any food item or mixed dish containing a significant amount of dairy, other than those provided<br>Node(s):<br>1.Full Fat, High Dairy<br>2. Mixed Dairy Products	Ad libitum	-	Assessment<br>IA1: Record of non-study dairy consumption (diary logs);<br>IA2+ IA3: Record of dairy consumption (diary logs), weighting of any returned (leftover) dairy foods<br>Degree<br>Consumption of non-study dairy foods over 12 wks:<br>IA1: 0.6±1.0 servings<br>IA2: 0.6±0.9 servings<br>IA3: 1.3±2.3 servings<br>Consumption of provided dairy foods:<br>IA2: 98.2%±1.8%<br>IA3: 97.9%±2.8%<br>Mean change energy intake (during intervention, full-fat diet differed from other groups):<br>IA1: 81±544 kcal/d<br>IA2: 224±375 kcal/d<br>IA3: 554±467 kcal/d
Tanaka, 2014 (53)	**Control**	**Dairy consumption**	-	Hypocaloric	Dietary counselling focused on weight control from registered	Assessment: 2-d dietary record weekly for the first 2 wk and then

	Node(s):<br>1. Control/ Low Dairy<br>2. Control	400g/d of milk, or a combination of milk plus yogurt;<br>Delivery of milk and dairy products free of charge for 24 weeks<br>Node(s):<br>1. High Dairy<br>2. Mixed Dairy Products			dieticians (90min session before intervention + advice throughout study);<br>Preferable energy intake calculated by the reference body weight multiplied by 25 to 30 kcal/kg/d	biweekly for the 3rd to the 24th week;<br>Dietitian help to ensure compliance<br>IA2: In addition, percent of days that subjects consumed the requisite amount of milk and dairy products in the first and second 12wk<br>Degree:<br>IA1: NR<br>IA2: first 12wk: 94.2%<br>second 12wk: 92.7%<br>Mean energy intake (24wk, P=0.99)<br>IA1: 1844±447 kcal/d<br>IA2: 1855±386 kcal/d
Thomas, 2010 (54)	**Low-calcium Diet**<br>(Ca ≥500 mg/d);<br>Maintain typical low calcium intake;<br>No consumption any dairy products or calcium supplements;<br>Avoidance any food with greater than 15% of daily calcium value per serving, and naturally occurring non-dairy calcium sources<br>Node(s):<br>1. Control/ Low Dairy<br>2. Control	**High Dairy-based Calcium Diet**<br>(Ca ≥1200 mg/d);<br>Instruction to increase dietary calcium by increasing dairy intake (3 servings of low-fat dairy foods per day);<br>Use of high-calcium food list and daily exchange plan to maintain their daily dietary intake goal of ≥1200mg<br>Node(s):<br>1. Low Fat, High Dairy	-	Hypocaloric	Modest energy reduction (250 kcal) from baseline energy needs;<br>Individualized counselling from a registered dietician;<br>Prescriptions for energy and daily calcium intake;<br>Prescribed diet was based on the American Diabetes Association exchange system (~15E% P, 55-60E% CHO, 25-30E% F);<br>Whole-body resistance training (3x/week for 16 weeks);	Assessment: Participant weight was documented weekly and used as a tool to assess diet adherence. In addition to monitoring body weight changes, participants met with the study dietitian 3x/wk prior to the exercise sessions to discuss dietary adherence and address diet-related questions<br>Degree:<br>Mean Ca intake:<br>IA1: 454±143 mg<br>IA2: 1312±183 mg

		2. Mixed Dairy Products			No additional exercise;<br>No use supplements;<br>Vitamin D- supplement (400 IU; daily) to prevent insufficient dietary intake	Mean protein intake:<br>IA1: 0.9 g/kg<br>IA2: 1.0 g/kg<br>Mean energy intake (16wk, P>0.05):<br>IA1: 1541±273 kcal/d<br>IA2: 1565±304 kcal/d
Thompson, 2005 (55)	**Standard Diet**<br>2 servings of dairy<br><br>Node(s):<br>1. Control/ Low Dairy<br>2. Control	**High Dairy**<br>4 servings of dairy (at least two of which were fluid milk)<br>Node(s):<br>1. High Dairy<br>2. Mixed Dairy Products	-	Hypocaloric	Caloric restriction (-500 calories with 30% F, 20% P, and 50% CHO); diet was designed to provide an average level of calcium and fiber (Ca intake was 932 mg, and the average fiber intake was 16.2 grams for the whole cohort);<br>Exercise (e.g., brisk walking, treadmill, or exercise bicycle; at least 30 minutes 4x/wk)	Assessment: Daily food diary which with weekly review of the dietician; (Participants who adhered to the plan were seen biweekly in the second one-half of the study; others were seen weekly); Discussion of problems by dieticians to enhance adherence; provision of education materials designed to enhance weight loss.<br>Degree:<br>Drop outs because inability to comply with weekly dietitian visits and food records:<br>IA1: n=3<br>IA2: n=4<br>Compliant with diet and exercise >75% of weeks;<br>IA1: n=18<br>IA2: n=18<br>Mean dairy servings/week:<br>IA1: 9.68±2.84

						IA2: 21.93± 3.91<br>Mean energy intake (during intervention, P>0.05):<br>IA1: 1427±268 kcal/d<br>IA2: 1490±234 kcal/d<br>Mean days recorded in diary (max.=336):<br>IA1: 299.55±50.2<br>IA2: 311.4± 29.0
Van Loan, 2011 (56)	**Low Dairy Diet**<br>≤1 serving/d e.g., milk, yogurt, or cheese<br>Node(s):<br>1. Control/ Low Dairy<br>2. Control	**Adequate Dairy Diet**<br>3-4 servings/d e.g., milk, yogurt, or cheese;<br>Dairy food servings consisted of 8 oz. (240 mL) low-fat (1%) or reduced-fat (2%) fluid milk, 8 oz. (240 mL) low-fat yogurt, 2 oz. (56 g) processed cheese or 1.5 oz. (42 g) natural cheese delivering 250–350 mg Ca, and at least 6 grams of dairy protein per serving<br>Node(s):<br>1. High Dairy<br>2. Mixed Dairy Products	**-**	Hypocaloric	Caloric restriction (−500 kcal/d; fat ~35% of total energy, carbohydrates ~49%, protein ~16%, and fiber 8–10 g/1000 kcal);<br>All foods were provided	Assessment:<br>Diet records and check of lists during in-house eating;<br>Compliance criteria: 1) ≤1 or 3-4 daily servings of dairy products, 2) energy intake within 200 kcal of energy prescription, 3) IA2 only: 95% consumption of the 294 total dairy servings during the 12wk intervention; Meeting all criteria = compliant for given wk; Total study compliance: 10/12wk compliant<br>Degree<br>IA1: all completers were compliant 36/40<br>IA2: all completers were compliant 35/38
Wennersberg, 2009 (57)	**Habitual Diet**	**Milk/Dairy Products**	**-**	Eucaloric	-	Assessment: 3-d diary records at 2 and 4 months

	Continuation of habitual diet without changing the intake of dairy products<br><br><u>Node(s):</u><br>1. Control/ Low Dairy<br>2. Control	3-5 portions of dairy products daily: milk 0.5–3% fat (one portion 200g), yogurt or sour milk (1.0–5.4% fat, 200–250g), cream or creme fraiche (12–40% fat, 75g), cheese (15–30%, 15–40g), butter/ butter containing spreads (40–80% fat, 3–10g), cottage cheese (2–8%, 0.5 dL), and ice cream occasionally<br><u>Node(s):</u><br>1. High Dairy<br>2. Mixed Dairy Products				and at the end of the study; Intermediate records were used to reinforce the dietary advice and strengthen adherence; at 2 and 4 months and at the end of the study;<br>Diary to record daily the number and types of dairy products consumed and any diversions from the intervention design<br><u>Degree</u><br>IA1: Dietary intake (baseline - after 26wk):<br>Total milk: -3g±106g<br>Cheese: -1g±25g<br>Butter: -0.6g±10g<br>IA2: Dietary intake (baseline - after 26wk):<br>Total milk: +249g±265g<br>Cheese: +20g±35g<br>Butter: +3g±20g<br>Mean energy intake (26wk, P>0.05):<br>IA1: 1875±518 kcal/d<br>IA2: 1986±646 kcal/d
Zemel, 2004 (58)	**Control**<br>0-1 servings of dairy products/d, 400-500 mg Ca/d<br><br><u>Node(s):</u><br>1. Control/ Low Dairy	**High Dairy**<br>3 daily servings of dairy products; Ca intake to 1200 to 1300mg/d<br><u>Node(s):</u><br>1. High Dairy	-	Hypocaloric	Caloric restriction (-500 kcal/d, balanced diet); Daily Placebo Supplement	<u>Assessment:</u> Daily diet diaries throughout the study; Weekly interviews; Review of the diet diary<br><u>Degree:</u><br>Ca intake (mg/d):

	2. Control	2. Mixed Dairy Products				IA1: 430±94;<br>IA2: 1137±164<br>Energy intake (during intervention):<br>IA1: 1309±253 kcal/d<br>IA2: 1370±216 kcal/d
Zemel, 2005 (phase 1) (59)	**Low Dairy**<br>≤1 serving/d and low Ca (500mg/d)<br><br>Node(s):<br>1. Control/ Low Dairy<br>2. Control	**High Dairy**<br>3 servings of dairy (1200 mg Ca/d);<br>at least one serving in the form of fluid milk<br>Node(s):<br>1. High Dairy<br>2. Mixed Dairy Products	-	Eucaloric	Isocaloric diet with macronutrient and fiber levels equivalent at levels approximating the current U.S. average	Assessment: Individual instruction, counselling, and assessment from the study dietitian regarding dietary adherence and the development and reinforcement of strategies for continued success; Weekly monitoring of diets<br>Degree:<br>Ca intake (mg/d):<br>IA1: 458±96<br>IA2: 1124±53<br>Energy intake (during intervention, P>0.05):<br>IA1: 1843±98 kcal/d<br>IA2: 1982±124 kcal/d
Zemel, 2005 (phase 2) (59)	**Low Dairy**<br>≤1 serving/d and low Ca (500 mg/d)<br><br>Node(s):<br>1. Control/ Low Dairy<br>2. Control	**High Dairy**<br>3 servings of dairy (1200 mg Ca/d);<br>at least one serving in the form of fluid milk<br>Node(s):<br>1. High Dairy<br>2. Mixed Dairy Products	-	Hypocaloric	Caloric restriction (-500 kcal/d based on initial estimate of caloric needs)	Assessment: Individual instruction, counselling, and assessment from the study dietitian regarding dietary adherence and the development and reinforcement of strategies for continued success; Weekly monitoring of diets<br>Degree:

						Ca intake (mg/d):<br>IA1: 468 ± 23<br>IA2: 1037 ± 27<br>Energy intake (during intervention):<br>IA1: 1278±84 kcal/d<br>IA2: 1491±62 kcal/d
Zemel, 2009 (60)	**Control**<br>0-1 servings of dairy products/day;<br>(Ca 500mg/d)<br><br>Node(s):<br>1. Control/ Low Dairy<br>2. Control	**High Dairy**<br>3 daily servings dairy products (milk, cheese and/or yogurt);<br>Substituted for other protein sources in the diet without altering macronutrient intake;<br>(Ca 1400mg/d)<br>Node(s):<br>1. High Dairy<br>2. Mixed Dairy Products	-	Hypocaloric	Caloric restriction (2,093 kJ/day deficit)<br>Daily placebo (methyl-cellulose) supplement (3x/d with meals)	Assessment: Compliance defined as <600 mg (IA1) or >900 mg (IA2) Ca/day, <1 (IA1) or ≥3 (IA2) daily serving of dairy, energy intake within 837 kJ of energy prescription, and return pill counts reflecting utilization of 80-100% of the placebo or calcium supplements provided each wk;<br>Total study compliance: Meeting weekly compliance for 75% of the wk<br>Degree:<br>IA1: 68.4% (26 of 38 subjects adherent)<br>IA2: 71.9% (23 of 32 subjects adherent)<br>Mean energy intake (during intervention):<br>IA1:1340±242 kcal/d<br>IA2:1519±267 kcal/d

Definition of nodes in NMA: 1. total dairy intake, 2. dairy product intake; *Cross-over study not considered in network meta-analysis as no data for the first intervention period was available; Abbreviations: Ca Calcium, CHO Carbohydrates, D Day, E Energy, F Fat, FFQ Food Frequency Questionnaire, IA Intervention Arm, Mo Month, NR Not Reported, P Protein, Wk Week

Control/low dairy: usual diet or a diet with 0-2 dairy servings/day or an equal amount in grams/day; high dairy: ≥3 dairy servings/day or an equal amount in grams/day; full fat dairy: dairy products with its natural fat content; low fat dairy: skimmed or semi-skimmed dairy products

Supplemental Table 9: GRADE evaluation of body weight (kg) for all comparisons (network total dairy intake)

		**Direct evidence**		**Indirect evidence**		**Network Meta-Analysis**	
Comparison	N studies	MD (95% CI)	Certainty of evidence	MD (95% CI)	Certainty of evidence	MD (95% CI)	Certainty of evidence
High dairy vs control/low dairy	10	−0.21 [−0.97; 0.56]	⊕⊕⊕○[1]	1.81 [−3.71; 7.32]	⊕⊕⊕○	−0.17 [−0.92; 0.59]	⊕⊕○○[2]
Full fat, high dairy vs control/low dairy	1	−4.20 [−14.22; 5.82]	⊕⊕⊕○[1]	3.18 [−21.43; 27.79]	⊕⊕⊕○	−3.15 [−12.43; 6.13]	⊕⊕○○[2]
Low fat, high dairy vs control/low dairy	5	0.31 [−0.77; 1.38]	⊕⊕⊕○[1]	−1.71 [−7.17; 3.75]	⊕⊕⊕○	0.23 [−0.82; 1.28]	⊕⊕○○[2]
High dairy vs full fat, high dairy	0	-	-	2.98 [−6.33; 12.29]	⊕⊕⊕○	2.98 [−6.33; 12.29]	⊕⊕○○[2]
High dairy vs low fat, high dairy	1	1.50 [−3.91; 6.91]	⊕⊕⊕○[1]	−0.51 [−1.83; 0.80]	⊕⊕⊕○	−0.40 [−1.68; 0.88]	⊕⊕○○[2]
Full fat, high dairy vs low fat, high dairy	1	−1.10 [−13.51; 11.31]	⊕⊕⊕○[1]	−6.31 [−20.37; 7.75]	⊕⊕⊕○	−3.38 [−12.68; 5.92]	⊕⊕○○[2]

⊕⊕⊕⊕ High; ⊕⊕⊕○ Moderate; ⊕⊕○○ Low; ⊕○○○ Very low

[1] downgraded by one level due to risk of bias (more than 2/3 of studies rated with some concerns)

[2] downgraded by one level due to imprecision (95% CI overlaps null effect)

Control/low dairy: usual diet or a diet with 0-2 dairy servings/day or an equal amount in grams/day; high dairy: ≥3 dairy servings/day or an equal amount in grams/day; full fat dairy: dairy products with its natural fat content; low fat dairy: skimmed or semi-skimmed dairy products

Supplemental Table 10: GRADE evaluation of body mass index (kg/m²) for all comparisons (network total dairy intake)

		**Direct evidence**		**Indirect evidence**		**Network Meta-Analysis**	
Comparison	N studies	MD (95% CI)	Certainty of evidence	MD (95% CI)	Certainty of evidence	MD (95% CI)	Certainty of evidence
High dairy vs control/low dairy	3	−0.02 [−0.49; 0.44]	⊕⊕⊕○[1]	1.31 [−0.15; 2.78]	⊕⊕⊕○	0.10 [−0.34; 0.54]	⊕⊕○○[2]
Low fat, high dairy vs control/low dairy	2	0.11 [−0.36; 0.58]	⊕⊕⊕○[1]	−1.22 [−2.69; 0.24]	⊕⊕⊕○	−0.01 [−0.46; 0.43]	⊕⊕○○[2]
High dairy vs low fat, high dairy	1	1.20 [−0.19; 2.59]	⊕⊕⊕○[1]	−0.14 [−0.80; 0.52]	⊕⊕⊕○	0.11 [−0.49; 0.71]	⊕⊕○○[2]

⊕⊕⊕⊕ High; ⊕⊕⊕○ Moderate; ⊕⊕○○ Low; ⊕○○○ Very low

[1] downgraded by one level due to risk of bias (more than 2/3 of studies rated with some concerns)

[2] downgraded by one level due to imprecision (95% CI overlaps null effect and sample size less than n=800)

Control/low dairy: usual diet or a diet with 0-2 dairy servings/day or an equal amount in grams/day; high dairy: ≥3 dairy servings/day or an equal amount in grams/day; full fat dairy: dairy products with its natural fat content; low fat dairy: skimmed or semi-skimmed dairy products

Supplemental Table 11: GRADE evaluation of fat mass (kg) for all comparisons (network total dairy intake)

		**Direct evidence**		**Indirect evidence**		**Network Meta-Analysis**	
Comparison	N studies	MD (95% CI)	Certainty of evidence	MD (95% CI)	Certainty of evidence	MD (95% CI)	Certainty of evidence
High dairy vs control/low dairy	9	−0.67 [−1.86; 0.51]	⨁⨁◯◯[1,2]	0.84 [−4.08; 5.77]	⨁⨁⨁◯	−0.59 [−1.74; 0.56]	⨁⨁◯◯[3]
Full fat, high dairy vs control/low dairy	1	−0.37 [−9.39; 8.65]	⨁⨁⨁◯[1]	7.38 [−21.68; 36.44]	⨁⨁⨁◯	0.31 [−8.30; 8.93]	⨁⨁◯◯[3]
Low fat, high dairy vs control/low dairy	4	1.34 [−0.98; 3.67]	⨁⨁⨁◯[1]	−0.17 [−4.67; 4.33]	⨁⨁◯◯	1.02 [−1.04; 3.09]	⨁⨁◯◯[3]
High dairy vs full fat, high dairy	0	-	-	−0.90 [−9.58, 7.78]	⨁⨁◯◯	−0.90 [−9.58, 7.78]	⨁◯◯◯[3]
High dairy vs low fat, high dairy	1	−0.50 [−4.84; 3.84]	⨁⨁⨁◯[1]	−2.02 [−4.63; 0.60]	⨁⨁◯◯	−1.61 [−3.85; 0.62]	⨁⨁◯◯[3]
Full fat, high dairy vs low fat, high dairy	1	1.17 [−10.26; 12.59]	⨁⨁⨁◯[1]	−3.34 [−16.87; 10.19]	⨁⨁⨁◯	−0.71 [−9.44; 8.02]	⨁⨁◯◯[3]

⨁⨁⨁⨁ High; ⨁⨁⨁◯ Moderate; ⨁⨁◯◯ Low; ⨁◯◯◯ Very low

[1] downgraded by one level due to risk of bias (more than 2/3 of studies rated with some concerns)

[2] downgraded by one level due to unexplained inconsistency (I^2=74%, p=0.0002, 95% CI do not always overlap)

[3] downgraded by one level due to imprecision (95% CI overlaps null effect)

Control/low dairy: usual diet or a diet with 0-2 dairy servings/day or an equal amount in grams/day; high dairy: ≥3 dairy servings/day or an equal amount in grams/day; full fat dairy: dairy products with its natural fat content; low fat dairy: skimmed or semi-skimmed dairy products

Supplemental Table 12: GRADE evaluation of waist circumference (cm) for all comparisons (network total dairy intake)

		**Direct evidence**		**Indirect evidence**		**Network Meta-Analysis**	
Comparison	N studies	MD (95% CI)	Certainty of evidence	MD (95% CI)	Certainty of evidence	MD (95% CI)	Certainty of evidence
High dairy vs control/low dairy	9	−1.45 [−3.51; 0.61]	⊕⊕○○[1,2]	0.84 [−6.83; 8.50]	⊕⊕⊕○	−1.29 [−3.29; 0.70]	⊕⊕○○[3]
Full fat, high dairy vs control/low dairy	1	3.80 [−5.39; 12.99]	⊕⊕⊕○[1]	−8.01 [−37.58; 21.55]	⊕⊕⊕○	2.76 [−6.01; 11.53]	⊕⊕○○[3]
Low fat, high dairy vs control/low dairy	2	−0.86 [−6.43; 4.71]	⊕⊕⊕○[1]	−3.15 [−8.80; 2.50]	⊕⊕⊕○	−1.99 [−5.96; 1.98]	⊕⊕○○[3]
High dairy vs full fat, high dairy	0	-	-	−4.05 [−12.98; 4.87]	⊕⊕○○	−4.05 [−12.98; 4.87]	⊕○○○[3]
High dairy vs low fat, high dairy	1	1.70 [−3.56; 6.96]	⊕⊕⊕○[1]	−0.59 [−6.53; 5.36]	⊕⊕⊕○	0.70 [−3.24; 4.63]	⊕⊕○○[3]
Full fat, high dairy vs low fat, high dairy	1	2.50 [−8.34; 13.34]	⊕⊕⊕○[1]	10.11 [−6.62; 26.84]	⊕⊕⊕○	4.75 [−4.35; 13.85]	⊕⊕○○[3]

⊕⊕⊕⊕ High; ⊕⊕⊕○ Moderate; ⊕⊕○○ Low; ⊕○○○ Very low

[1] downgraded by one level due to risk of bias (more than 2/3 of studies rated with some concerns)

[2] downgraded by one level due to unexplained inconsistency (I^2=83%, P<0.001, 95% CI do not always overlap)

[3] downgraded by one level due to imprecision (95% CI overlaps null effect)

Control/low dairy: usual diet or a diet with 0-2 dairy servings/day or an equal amount in grams/day; high dairy: ≥3 dairy servings/day or an equal amount in grams/day; full fat dairy: dairy products with its natural fat content; low fat dairy: skimmed or semi-skimmed dairy products

Supplemental Table 13: GRADE evaluation of body weight (kg) for all comparisons (network dairy product intake)

		**Direct evidence**		**Indirect evidence**		**Network Meta-Analysis**	
Comparison	N studies	MD (95% CI)	Certainty of evidence	MD (95% CI)	Certainty of evidence	MD (95% CI)	Certainty of evidence
Milk vs Control	1	−3.60 [−17.78; 10.58]	⊕⊕⊕◯[1]	-	-	−3.60 [−17.78; 10.58]	⊕⊕◯◯[2]
Yogurt vs Control	1	−0.50 [−7.31; 6.31]	⊕⊕⊕◯[1]	-	-	−0.50 [−7.31; 6.31]	⊕⊕◯◯[2]
Kefir vs Control	0	-	-	−3.60 [−19.46; 12.26]	⊕⊕⊕◯	−3.60 [−19.46; 12.26]	⊕⊕◯◯[2]
Mixed Dairy vs Control	13	−0.09 [−0.82; 0.65]	⊕⊕⊕◯[1]	-	-	−0.09 [−0.82; 0.65]	⊕⊕◯◯[2]
Milk vs Yogurt	0	-	-	−3.10 [−18.83; 12.63]	⊕⊕⊕◯	−3.10 [−18.83; 12.63]	⊕⊕◯◯[2]
Milk vs Mixed Dairy	0	-	-	−3.51 [−17.71; 10.68]		−3.51 [−17.71; 10.68]	⊕⊕◯◯[2]
Kefir vs Milk	1	0.00 [−7.11; 7.11]	⊕⊕⊕◯[1]	-	-	0.00 [−7.11; 7.11]	⊕⊕◯◯[2]
Kefir vs Yogurt	0	-	-	−3.10 [−20.36; 14.16]	⊕⊕⊕◯	−3.10 [−20.36; 14.16]	⊕⊕◯◯[2]
Kefir vs Mixed Dairy	0	-	-	−3.51 [−19.39; 12.36]	⊕⊕⊕◯	−3.51 [−19.39; 12.36]	⊕⊕◯◯[2]
Mixed Dairy vs Yogurt	0	-	-	0.41 [−6.44; 7.27]	⊕⊕⊕◯	0.41[−6.44; 7.27]	⊕⊕◯◯[2]

⊕⊕⊕⊕ High; ⊕⊕⊕◯ Moderate; ⊕⊕◯◯ Low; ⊕◯◯◯ Very low

[1] downgraded by one level due to risk of bias (more than 2/3 of studies rated with some concerns)

[2] downgraded by one level due to imprecision (95% CI overlaps null effect)

Supplemental Table 14: GRADE evaluation of BMI (kg/m²) for all comparisons (network dairy product intake)

		Direct evidence		Indirect evidence		Network Meta-Analysis	
Comparison	N studies	MD (95% CI)	Certainty of evidence	MD (95% CI)	Certainty of evidence	MD (95% CI)	Certainty of evidence
Milk vs Control	1	0.30 [−2.37; 2.97]	⊕⊕⊕○[1]	-	-	0.30 [−2.37; 2.97]	⊕⊕○○[2]
Yogurt vs Control	0	-	-	0.10 [−2.92; 3.12]	⊕⊕⊕○	0.10 [−2.92; 3.12]	⊕⊕○○[2]
Kefir vs Control	0	-	-	0.10 [−3.78; 3.98]	⊕⊕⊕○	0.10 [−3.78; 3.98]	⊕⊕○○[2]
Mixed Dairy vs Control	4	0.04 [−0.29; 0.37]	⊕⊕⊕○[1]	-	-	0.04 [−0.29; 0.37]	⊕⊕○○[2]
Milk vs Yogurt	1	0.20 [−1.21; 1.61]	⊕⊕⊕○[1]	-	-	0.20 [−1.21; 1.61]	⊕⊕○○[2]
Milk vs Mixed Dairy	0	-	-	0.26 [−2.43; 2.95]	⊕⊕⊕○	0.26 [−2.43; 2.95]	⊕⊕○○[2]
Kefir vs Milk	1	−0.20 [−3.02; 2.62]	⊕⊕⊕○[1]	-	-	−0.20 [−3.02; 2.62]	⊕⊕○○[2]
Kefir vs Yogurt	0	-	-	−0.00 [−3.15; 3.15]	⊕⊕⊕○	−0.00 [−3.15; 3.15]	⊕⊕○○[2]
Kefir vs Mixed Dairy	0	-	-	0.06 [−3.83; 3.95]	⊕⊕⊕○	0.06 [−3.83; 3.95]	⊕⊕○○[2]
Mixed Dairy vs Yogurt	0	-	-	−0.06 [−3.10; 2.97]	⊕⊕⊕○	−0.06 [−3.10; 2.97]	⊕⊕○○[2]

⊕⊕⊕⊕ High; ⊕⊕⊕○ Moderate; ⊕⊕○○ Low; ⊕○○○ Very low

[1] downgraded by one level due to risk of bias (more than 2/3 of studies rated with some concerns)

[2] downgraded by one level due to imprecision (95% CI overlaps null effect and sample size less than n=800)

Supplemental Table 15: GRADE evaluation of fat mass (kg) for all comparisons (network dairy product intake)

		Direct evidence		Indirect evidence		Network Meta-Analysis	
Comparison	N studies	MD (95% CI)	Certainty of evidence	MD (95% CI)	Certainty of evidence	MD (95% CI)	Certainty of evidence
Milk vs Control	1	−0.00 [−8.48; 8.48]	⊕⊕⊕○[1]	-	-	−0.00 [−8.48; 8.48]	⊕⊕○○[3]
Yogurt vs Control	0	-	-	−2.56 [−11.86; 6.74]	⊕⊕⊕○	−2.56 [−11.86; 6.74]	⊕⊕○○[3]
Kefir vs Control	0	-	-	0.40 [−9.99; 10.79]	⊕⊕⊕○	0.40 [−9.99; 10.79]	⊕⊕○○[3]
Mixed Dairy vs Control	12	−0.26 [−1.33; 0.81]	⊕⊕○○[1,2]	-	-	−0.26 [−1.33; 0.81]	⊕○○○[3]
Milk vs Yogurt	1	2.56 [−1.25; 6.37]	⊕⊕⊕○[1]	-	-	2.56 [−1.25; 6.37]	⊕⊕○○[3]
Milk vs Mixed Dairy	0	-	-	0.26 [−8.29; 8.81]	⊕⊕○○	0.26 [−8.29; 8.81]	⊕○○○[3]
Kefir vs Milk	1	0.40 [−5.61; 6.41]	⊕⊕⊕○[1]	-	-	0.40 [−5.61; 6.41]	⊕⊕○○[3]
Kefir vs Yogurt	0	-	-	2.96 [−4.15; 10.07]	⊕⊕⊕○	2.96 [−4.15; 10.07]	⊕⊕○○[3]
Kefir vs Mixed Dairy	0	-	-	0.66 [−9.79; 11.11]	⊕⊕○○	0.66 [−9.79; 11.11]	⊕○○○[3]
Mixed Dairy vs Yogurt	0	-	-	2.30 [−7.06; 11.66]	⊕⊕○○	2.30 [−7.06; 11.66]	⊕○○○[3]

⊕⊕⊕⊕ High; ⊕⊕⊕○ Moderate; ⊕⊕○○ Low; ⊕○○○ Very low

[1] downgraded by one level due to risk of bias (more than 2/3 of studies rated with some concerns)

[2] downgraded by one level due to unexplained inconsistency (I^2=71%, p<0.0001, 95% CI do not always overlap)

[3] downgraded by one level due to imprecision (95% CI overlaps null effect)

Supplemental Table 16: GRADE evaluation of waist circumference (cm) for all comparisons (disconnected; network dairy product intake)

		**Direct evidence**		**Indirect evidence**		**Network Meta-Analysis**	
Comparison	N studies	MD (95% CI)	Certainty of evidence	MD (95% CI)	Certainty of evidence	MD (95% CI)	Certainty of evidence
Mixed Dairy vs Control	11	−1.27[−3.20; 0.67]	⊕⊕○○[1,2]				-
Milk vs Yogurt	1	3.47 [0.02; 6.92]	⊕⊕⊕○[1]			3.47 [0.02; 6.92]	⊕⊕○○[3]
Kefir vs Milk	1	−0.2 [−4.73; 4.32]	⊕⊕⊕○[1]			−0.2 [−4.73; 4.32]	⊕⊕○○[3]
Kefir vs Yogurt	0	-		3.27[−2.42;8.96]	⊕⊕⊕○	3.27 [−2.42; 8.96]	⊕⊕○○[3]

⊕⊕⊕⊕ High; ⊕⊕⊕○ Moderate; ⊕⊕○○ Low; ⊕○○○ Very low

[1] downgraded by one level due to risk of bias (more than 2/3 of studies rated with some concerns)

[2] downgraded by one level due to unexplained inconsistency (I^2=80%, p<0.0001, 95% CI do not always overlap)

[3] downgraded by one level due to imprecision (95% CI overlaps null effect and sample size less than n=800)

Supplemental Table 17: GRADE evaluation of LDL-C (mmol/L) for all comparisons (network total dairy intake)

		**Direct evidence**		**Indirect evidence**		**Network Meta-Analysis**	
Comparison	N studies	MD (95% CI)	Certainty of evidence	MD (95% CI)	Certainty of evidence	MD (95% CI)	Certainty of evidence
High dairy vs control/low dairy	6	0.09 [−0.07; 0.24]	⊕⊕⊕○[1]	0.05 [−0.46; 0.55]	⊕⊕⊕○	0.08 [−0.06; 0.23]	⊕⊕○○[2]
Full fat, high dairy vs control/low dairy	1	0.32 [−0.16; 0.80]	⊕⊕⊕○[1]	−0.20 [−1.30; 0.90]	⊕⊕⊕○	0.24 [−0.21; 0.68]	⊕⊕○○[2]
Low fat, high dairy vs control/low dairy	2	−0.01 [−0.36; 0.33]	⊕⊕⊕○[1]	0.03 [−0.38; 0.43]	⊕⊕⊕○	0.00 [−0.26; 0.27]	⊕⊕○○[2]
High dairy vs full fat, high dairy		-	-	−0.15 [−0.61; 0.30]	⊕⊕⊕○	−0.15 [-0.61; 0.30]	⊕⊕○○[2]
High dairy vs low fat, high dairy	1	0.06 [−0.32; 0.44]	⊕⊕⊕○[1]	0.10 [−0.28; 0.48]	⊕⊕⊕○	0.08 [−0.19; 0.35]	⊕⊕○○[2]
Full fat, high dairy vs low fat, high dairy	1	0.16 [−0.31; 0.63]	⊕⊕⊕○[1]	0.72 [−0.49; 1.93]	⊕⊕⊕○	0.23 [−0.20; 0.67]	⊕⊕○○[2]

⊕⊕⊕⊕ High; ⊕⊕⊕○ Moderate; ⊕⊕○○ Low; ⊕○○○ Very low

[1] downgraded by one level due to risk of bias (more than 2/3 of studies rated with some concerns)

[2] downgraded by one level due to imprecision (95% CI overlaps null effect and sample size less than n=800)

Control/low dairy: usual diet or a diet with 0-2 dairy servings/day or an equal amount in grams/day; high dairy: ≥3 dairy servings/day or an equal amount in grams/day; full fat dairy: dairy products with its natural fat content; low fat dairy: skimmed or semi-skimmed dairy products

Supplemental Table 18: GRADE evaluation of triglycerides (mmol/L) for all comparisons (network total dairy intake)

		**Direct evidence**		**Indirect evidence**		**Network Meta-Analysis**	
Comparison	N studies	MD (95% CI)	Certainty of evidence	MD (95% CI)	Certainty of evidence	MD (95% CI)	Certainty of evidence
High dairy vs control/low dairy	6	−0.05 [−0.15; 0.05]	⊕⊕⊕○[1]	0.42 [−0.03; 0.86]	⊕⊕⊕○	−0.03 [−0.12; 0.07]	⊕⊕○○[2]
Full fat, high dairy vs control/low dairy	1	0.05 [−0.46; 0.55]	⊕⊕⊕○[1]	−0.53 [−1.48; 0.43]	⊕⊕⊕○	−0.08 [−0.52; 0.37]	⊕⊕○○[2]
Low fat, high dairy vs control/low dairy	2	0.23 [−0.13; 0.59]	⊕⊕⊕○[1]	−0.24 [−0.51; 0.04]	⊕⊕⊕○	−0.07 [−0.29; 0.15]	⊕⊕○○[2]
High dairy vs full fat, high dairy	0	-	-	0.05 [−0.40; 0.50]	⊕⊕⊕○	0.05 [−0.40; 0.50]	⊕⊕○○[2]
High dairy vs low fat, high dairy	1	0.19 [−0.07; 0.45]	⊕⊕⊕○[1]	−0.28 [−0.65; 0.10]	⊕⊕⊕○	0.04 [−0.17; 0.25]	⊕⊕○○[2]
Full fat, high dairy vs low fat, high dairy	1	−0.15 [−0.68; 0.37]	⊕⊕⊕○[1]	0.39 [−0.48; 1.26]	⊕⊕⊕○	−0.01 [−0.46; 0.44]	⊕⊕○○[2]

⊕⊕⊕⊕ High; ⊕⊕⊕○ Moderate; ⊕⊕○○ Low; ⊕○○○ Very low

[1] downgraded by one level due to risk of bias (more than 2/3 of studies rated with some concerns)

[2] downgraded by one level due to imprecision (95% CI overlaps null effect and sample size less than n=800)

Control/low dairy: usual diet or a diet with 0-2 dairy servings/day or an equal amount in grams/day; high dairy: ≥3 dairy servings/day or an equal amount in grams/day; full fat dairy: dairy products with its natural fat content; low fat dairy: skimmed or semi-skimmed dairy products

Supplemental Table 19: League tables presenting results (mean differences with 95% confidence intervals) of the network meta-analysis on total dairy intake with a combined control and low dairy group (white fields) as well as results of pairwise meta-analyses (light grey fields) for (a) HDL-C, (b) fasting glucose, (c) Glycated hemoglobin (d) energy intake (outcomes without GRADE)

A) HDL-C (mmol/L)

**Control/LowDairy**	-0.03 [-0.09; 0.02]	**-0.27 [-0.50; -0.03]**	-0.06 [-0.20; 0.09]
-0.03 [-0.09; 0.02]	**High Dairy**	.	-0.01 [-0.16; 0.14]
**-0.26 [-0.49; -0.03]**	-0.22 [-0.46; 0.01]	**Full Fat, High Dairy**	0.18 [-0.08; 0.44]
-0.05 [-0.16; 0.06]	-0.02 [-0.12; 0.09]	0.21 [-0.03; 0.44]	**Low Fat, High Dairy**

B) Fasting glucose (mmol/L)

**Control/LowDairy**	0.01 [-0.17; 0.19]	**-0.59 [-1.06; -0.12]**	**-0.51 [-0.88; -0.14]**
-0.03 [-0.20; 0.14]	**High Dairy**	.	-0.03 [-0.45; 0.39]
**-0.43 [-0.86; -0.01]**	-0.40 [-0.85; 0.04]	**Full Fat, High Dairy**	0.02 [-0.41; 0.45]
**-0.31 [-0.60; -0.03]**	-0.28 [-0.58; 0.01]	0.12 [-0.29; 0.53]	**Low Fat, High Dairy**

C) Glycated hemoglobin (%)

**Control/LowDairy**	-0.07 [-0.21; 0.08]	**-0.37 [-0.61; -0.13]**	**-0.47 [-0.78; -0.15]**
-0.07 [-0.22; 0.08]	**High Dairy**	.	.
**-0.37 [-0.61; -0.13]**	**-0.30 [-0.58; -0.02]**	**Full Fat, High Dairy**	-0.10 [-0.40; 0.20]
**-0.47 [-0.78; -0.15]**	**-0.40 [-0.75; -0.05]**	-0.10 [-0.40; 0.20]	**Low Fat, High Dairy**

D) Energy intake (kcal/d)

**Control/LowDairy**	**-123.52 [-190.43; -56.60]**	**-495.00 [-813.12; -176.88]**	-9.05 [-83.63; 65.51]
**-117.72 [-182.85; -52.58]**	**High Dairy**	.	3.82 [-270.82; 278.47]
**-391.85 [-643.64; -140.07]**	-274.13 [-533.43; -14.84]	**Full Fat, High Dairy**	**330.00 [67.75; 592.25**]
-16.25 [-88.36; 55.85]	**101.47 [7.34; 195.59]**	**375.60 [127.84; 623.36]**	**Low Fat, High Dairy**

Control/low dairy: usual diet or a diet with 0-2 dairy servings/day or an equal amount in grams/day; high dairy: ≥3 dairy servings/day or an equal amount in grams/day; full fat dairy: dairy products with its natural fat content; low fat dairy: skimmed or semi-skimmed dairy products

Supplemental Table 20: GRADE evaluation of triglycerides (mmol/L) for all comparisons (network dairy product intake)

		**Direct evidence**		**Indirect evidence**		**Network Meta-Analysis**	
Comparison	N studies	MD (95% CI)	Certainty of evidence	MD (95% CI)	Certainty of evidence	MD (95% CI)	Certainty of evidence
Milk vs Control	1	0.25 [−0.23; 0.73]	⊕⊕⊕○[1]	-	-	0.25 [−0.23; 0.73]	⊕⊕○○[2]
Yogurt vs Control	0	-	-	−0.13 [−0.73; 0.47]	⊕⊕⊕○	−0.13 [−0.73; 0.47]	⊕⊕○○[2]
Kefir vs Control	0	-	-	0.52 [−0.21; 1.25]	⊕⊕⊕○	0.52 [−0.21; 1.25]	⊕⊕○○[2]
Mixed Dairy vs Control	7	−0.04 [−0.14; 0.05]	⊕⊕⊕○[1]	-	-	−0.04 [−0.14; 0.05]	⊕⊕○○[2]
Milk vs Yogurt	1	**0.38 [0.03; 0.73]**	⊕⊕⊕○[1]	-	-	**0.38 [0.03; 0.73]**	⊕⊕○○[3]
Milk vs Mixed Dairy	0	-	-	0.29 [−0.20; 0.78]	⊕⊕⊕○	0.29 [−0.20; 0.78]	⊕⊕○○[2]
Kefir vs Milk	1	0.27 [−0.28; 0.82]	⊕⊕⊕○[1]	-	-	−0.21[−0.92; 0.51]	⊕⊕○○[2]
Kefir vs Yogurt	0	-	-	0.52 [−0.21; 1.25]	⊕⊕⊕○	0.52 [−0.21; 1.25]	⊕⊕○○[2]
Kefir vs Mixed Dairy	0	-	-	0.56 [−0.18; 1.30]	⊕⊕⊕○	0.56 [−0.18; 1.30]	⊕⊕○○[2]
Mixed Dairy vs Yogurt	0	-	-	0.09 [−0.51; 0.69]	⊕⊕⊕○	0.09 [−0.51; 0.69]	⊕⊕○○[2]

⊕⊕⊕⊕ High; ⊕⊕⊕○ Moderate; ⊕⊕○○ Low; ⊕○○○ Very low

[1] downgraded by one level due to risk of bias (more than 2/3 of studies rated with some concerns)

[2] downgraded by one level due to imprecision (95% CI overlaps null effect and sample size less than n=800)

[3] downgraded by one level due to imprecision (sample size less than n=800)

Supplemental Table 21: League tables presenting results (mean differences with 95% confidence intervals) of the network meta-analysis on dairy product intake as well as results of pairwise meta-analyses (light grey fields) for (a) HDL-C, (b) fasting glucose, (c) energy intake (outcomes without GRADE).

A) HDL-C (mmol/L)

**Control**	.	-0.02 [-0.26; 0.22]	-0.05 [-0.12; 0.02]	.
0.00[-0.29; 0.29]	**Kefir**	-0.02 [-0.19; 0.15]	.	.
-0.02 [-0.26; 0.22]	-0.02 [-0.19; 0.15]	**Milk**	.	**-0.19 [-0.38; -0.00]**
-0.05 [-0.12; 0.02]	-0.05 [-0.35; 0.25]	-0.03 [-0.28; 0.21]	**Mixed Dairy Products**	**.**
-0.21 [-0.51; 0.09]	-0.21 [-0.46; 0.04]	**-0.19 [-0.38; -0.00]**	-0.16 [-0.47; 0.15]	**Yogurt**

B) Fasting glucose (mmol/L)

**Control**	.	-0.34 [-1.09; 0.41]	-0.07 [-0.28; 0.13]	.
-0.61 [-1.56; 0.34]	**Kefir**	0.27 [-0.32; 0.86]	.	.
-0.34 [-1.09; 0.45]	0.27 [-0.32; 0.86]	**Milk**	.	0.07 [-0.38; 0.52]
-0.07 [-0.28; 0.13]	0.54 [-0.44; 1.51]	0.27 [-0.51; 1.04]	**Mixed Dairy Products**	**.**
-0.27 [-1.15; 0.61]	0.34 [-0.40; 1.08]	0.07 [-0.38; 0.52]	-0.20 [-1.20; 0.70]	**Yogurt**

C) Energy intake (kcal/d)

**Control**	.	-45.86 [-640.22; 548.50]	-109.98 [-189.34; -30.62]	-288.77 [-683.77; 106.24]
-316.15 [-850.18; 217.87]	**Kefir**	45.00 [-293.32; 383.32]	.	.
-271.15 [-684.34; 142.04]	45.00 [-293.32; 383.32]	**Milk**	.	193.10 [-224.48; 610.68]
-109.98 [-189.34; -30.62]	206.17 [-333.72; 746.06]	161.17 [-259.57; 581.91]	**Mixed Dairy Products**	**.**
-189.26 [-536.27; 157.76]	126.90 [-367.43; 621.22]	81.90 [-278.52; 442.31]	-79.28 [-435.25; 276.70]	**Yogurt**

Supplemental Table 22: GRADE evaluation of LDL-C (mmol/L) for all comparisons (network dairy product intake)

		**Direct evidence**		**Indirect evidence**		**Network Meta-Analysis**	
Comparison	N studies	MD (95% CI)	Certainty of evidence	MD (95% CI)	Certainty of evidence	MD (95% CI)	Certainty of evidence
Milk vs Control	1	−0.27 [−0.81; 0.27]	⊕⊕⊕○[1]	-	-	−0.27 [−0.81; 0.27]	⊕⊕○○[2]
Yogurt vs Control	0	-	-	−0.37 [−1.01; 0.27]	⊕⊕⊕○	−0.37 [−1.01; 0.27]	⊕⊕○○[2]
Kefir vs Control	0	-	-	−0.35 [−1.06; 0.36]	⊕⊕⊕○	−0.35 [−1.06; 0.36]	⊕⊕○○[2]
Mixed Dairy vs Control	7	0.11 [−0.03; 0.25]	⊕⊕⊕○[1]	-	-	0.11 [−0.03; 0.25]	⊕⊕○○[2]
Milk vs Yogurt	1	0.10 [−0.24; 0.44]	⊕⊕⊕○[1]			0.10 [−0.24; 0.44]	⊕⊕○○[2]
Milk vs Mixed Dairy	0	-	-	−0.38 [−0.93; 0.18]	⊕⊕⊕○	−0.38 [−0.93; 0.18]	⊕⊕○○[2]
Kefir vs Milk	1	−0.08 [−0.55; 0.39]	⊕⊕⊕○[1]	-	-	−0.08 [−0.55; 0.39]	⊕⊕○○[2]
Kefir vs Yogurt	0	-	-	0.02 [−0.56; 0.60]	⊕⊕⊕○	0.02 [−0.56; 0.60]	⊕⊕○○[2]
Kefir vs Mixed Dairy	0	-	-	−0.46 [−1.18; 0.27]	⊕⊕⊕○	−0.46 [−1.18; 0.27]	⊕⊕○○[2]
Mixed Dairy vs Yogurt	0	-	-	0.48 [−0.17; 1.13]	⊕⊕⊕○	0.48 [−0.17; 1.13]	⊕⊕○○[2]

⊕⊕⊕⊕ High; ⊕⊕⊕○ Moderate; ⊕⊕○○ Low; ⊕○○○ Very low

[1] downgraded by one level due to risk of bias (more than 2/3 of studies rated with some concerns)

[2] downgraded by one level due to imprecision (95% CI overlaps null effect and sample size less than n=800)

Supplemental Table 23: Results (mean differences with 95% confidence intervals) of pairwise meta-analyses using random effects model on mixed dairy products vs. control and kefir vs. milk for glycated hemoglobin (%)

Comparison	N studies	MD (95% CI)	I^2	Tau^2	Test of heterogeneity
Mixed Dairy vs Control	3	0.16 [−0.12; 0.43]	79.9 [36.2; 93.6]%	0.0475 [0.0036; 2.7209]	P=0.007
Kefir vs Milk	1	0.20 [−0.05; 0.45]	-	-	-

Supplemental Table 24: GRADE evaluation of systolic blood pressure (mmHg) for all comparisons network total dairy intake)

		**Direct evidence**		**Indirect evidence**		**Network Meta-Analysis**	
Comparison	N studies	MD (95% CI)	Certainty of evidence	MD (95% CI)	Certainty of evidence	MD (95% CI)	Certainty of evidence
High dairy vs control/low dairy	5	−1.64 [−4.73; 1.45]	⊕⊕○○[1,2]	1.09 [−8.35; 10.52]	⊕⊕⊕○	−1.37 [−4.31; 1.56]	⊕⊕○○[3]
Full fat, high dairy vs control/low dairy	1	−7.67 [−17.95; 2.61]	⊕⊕⊕○[1]	−7.19 [−33.57; 19.19]	⊕⊕⊕○	−7.60 [−17.18; 1.97]	⊕⊕○○[3]
Low fat, high dairy vs control/low dairy	2	−4.21 [−10.25; 1.83]	⊕⊕⊕○[1]	−6.94 [−14.82; 0.94]	⊕⊕○○	**−5.22 [−10.01; −0.43]**	⊕⊕○○[4]
High dairy vs full fat, high dairy	0	-	-	6.23 [-3.59; 16.05]	⊕⊕○○		⊕○○○[3]
High dairy vs low fat, high dairy	1	5.30 [−1.95; 12.55]	⊕⊕⊕○[1]	2.57 [−4.21; 9.36]	⊕⊕○○	3.85 [−1.11; 8.80]	⊕⊕○○[3]
Full fat, high dairy vs low fat, high dairy	1	−2.33 [−12.22; 7.55]	⊕⊕⊕○[1]	−2.89 [−34.52; 28.75]	⊕⊕○○	−2.38 [−11.82; 7.05]	⊕⊕○○[3]

⊕⊕⊕⊕ High; ⊕⊕⊕○ Moderate; ⊕⊕○○ Low; ⊕○○○ Very low

[1] downgraded by one level due to risk of bias (more than 2/3 of studies rated with some concerns)

[2] downgraded by one level due to unexplained inconsistency (I^2=57%, P=0.0518, (non) significant effects in both directions, only slight overlap of 95% CI)

[3] downgraded by one level due to imprecision (95% CI overlaps minimal effect (2 mmHg) and sample size less than n=800)

[4] downgraded by one level due to imprecision (sample size less than n=800)

Control/low dairy: usual diet or a diet with 0-2 dairy servings/day or an equal amount in grams/day; high dairy: ≥3 dairy servings/day or an equal amount in grams/day; full fat dairy: dairy products with its natural fat content; low fat dairy: skimmed or semi-skimmed dairy products

Supplemental Table 25: GRADE evaluation of systolic blood pressure (mmHG) for all comparisons (network dairy product intake)

		**Direct evidence**		**Indirect evidence**		**Network Meta-Analysis**	
Comparison	N studies	MD (95% CI)	Certainty of evidence	MD (95% CI)	Certainty of evidence	MD (95% CI)	Certainty of evidence
Milk vs Control	1	−2.90 [−12.60; 6.80]	⊕⊕⊕○[1]	-	-	−2.90 [−12.60; 6.80]	⊕⊕○○[3]
Yogurt vs Control	0	-	-	0.86 [−14.13; 15.85]	⊕⊕⊕○	0.86 [−14.13; 15.85]	⊕⊕○○[3]
Kefir vs Control	0	-	-	−5.90 [−19.64; 7.84]	⊕⊕⊕○	−5.90 [−19.64; 7.84]	⊕⊕○○[3]
Mixed Dairy vs Control	6	−2.28 [−5.60; 1.03]	⊕⊕○○[1,2]	-	-	−2.28 [−5.60; 1.03]	⊕○○○[3]
Milk vs Yogurt	1	−3.76 [−15.19; 7.67]	⊕⊕⊕○[1]	-	-	−3.76 [−15.19; 7.67]	⊕⊕○○[3]
Milk vs Mixed Dairy	0	-	-	−0.62 [−10.87; 9.63]	⊕⊕○○	−0.62 [−10.87; 9.63]	⊕○○○[3]
Kefir vs Milk	1	−3.00 [−12.73; 6.73]	⊕⊕⊕○[1]	-	-	−3.00 [−12.73; 6.73]	⊕⊕○○[3]
Kefir vs Yogurt	0	-	-	−6.76 [−21.77; 8.25]	⊕⊕⊕○	−6.76 [−21.77; 8.25]	⊕⊕○○[3]
Kefir vs Mixed Dairy	0	-	-	−3.62 [−17.75; 10.51]	⊕⊕○○	−3.62 [−17.75; 10.51]	⊕○○○[3]
Mixed Dairy vs Yogurt	0	-	-	−3.14 [−18.49; 12.21]	⊕⊕○○	−3.14 [−18.49; 12.21]	⊕○○○[3]

⊕⊕⊕⊕ High; ⊕⊕⊕○ Moderate; ⊕⊕○○ Low; ⊕○○○ Very low

[1] downgraded by one level due to risk of bias (more than 2/3 of studies rated with some concerns)

[2] downgraded by one level due to unexplained inconsistency (I^2=55%, P=0.047, (non) significant effects in both directions, 95% CI do not always overlap)

[3] downgraded by one level due to imprecision (95% CI overlaps minimal effect (2 mmHg) and sample size less than n=800)

Supplemental Table 26: Relative ranking of interventions on total dairy intake (P-scores*)

Intervention	BW (↓)	BMI (↓)	FM (↓)	WC (↓)	LDL-C (↓)	HDL-C (↑)	TG (↓)	FG (↓)	HbA1c (↓)	SBP (↓)
Full fat, high dairy	**0.75**	-	0.49	0.20	0.18	**0.97**	0.58	0.12	0.25	**0.84**
Low fat, high dairy	0.28	0.58	0.23	**0.77**	0.69	0.49	**0.62**	0.25	0.09	0.74
High dairy	0.56	0.35	**0.78**	0.69	0.38	0.44	0.49	0.76	0.72	0.33
Control/low dairy	0.42	0.57	0.51	0.33	**0.74**	0.10	0.31	**0.87**	**0.94**	0.09

*P-scores were calculated and presented to obtain relative ranking of nutrition interventions. Higher P-score value indicates greater benefit (larger decrease or increase in outcome of interest) with a certain intervention.

Abbreviations: BMI Body Mass Index, BW Body Weight, FG Fasting Glucose, FM Fat Mass, HbA1c Glycated Hemoglobin, HDL-C High-Density Lipoprotein Cholesterol, LDL-C Low-Density Lipoprotein Cholesterol, TG Triacylglycerol, SBP Systolic Blood Pressure, WC Waist Circumference. Bolded are interventions on total dairy intake identified as the best for the given outcome.

(↓) = decrease is the effect of interest; (↑) = increase is the effect of interest; (↔) stable intake is of interest

Control/low dairy: usual diet or a diet with 0-2 dairy servings/day or an equal amount in grams/day; high dairy: ≥3 dairy servings/day or an equal amount in grams/day; full fat dairy: dairy products with its natural fat content; low fat dairy: skimmed or semi-skimmed dairy products

Supplemental Table 27: Relative ranking of interventions on intake of dairy products (P-scores*)

Intervention	BW (↓)	BMI (↓)	FM (↓)	WC (↓)	LDL-C (↓)	HDL-C (↑)	TG (↓)	FG (↓)	HbA1c (↓)	SBP (↓)
Mixed dairy products	0.63	0.50	0.52	NA	0.09	0.58	0.75	0.63	NA	0.58
Kefir	0.62	0.51	0.39	NA	0.71	0.33	0.09	0.15	NA	**0.76**
Yogurt	0.45	0.52	**0.77**	**NA**	**0.80**	**0.92**	0.81	0.51	**NA**	0.31
Milk	0.63	0.42	0.41	NA	0.60	0.40	0.28	0.41	NA	0.57
Control	0.37	0.55	0.41	NA	0.35	0.27	0.57	**0.80**	NA	0.28

*P-scores were calculated and presented to obtain relative ranking of nutrition interventions. Higher P-score value indicates greater benefit (larger decrease or increase in outcome of interest) with a certain intervention.
Abbreviations: BMI Body Mass Index, BW Body Weight, FG Fasting Glucose, FM Fat Mass, HbA1c Glycated Hemoglobin, HDL-C High-Density Lipoprotein Cholesterol, LDL-C Low-Density Lipoprotein Cholesterol, NA Not Applicable due to disconnected network, TG Triacylglycerol, SBP Systolic Blood Pressure, WC Waist Circumference.
Bolded are interventions on intake of dairy products identified as the best for the given outcome.
(↓) = decrease is the effect of interest; (↑) = increase is the effect of interest; (↔) stable intake is of interest

	D1	D2	D3	D4	D5	Overall
Bellikci-Koyu 2022 (Anthopometry: BW, BMI, FM,WC)	+	-	-	+	-	-
Bellikci-Koyu 2022 (Blood markers: LDL-C, HDL-C, TG, FG, HbA1c)	+	-	-	+	-	-
Bellikci-Koyu 2022 (Systolic Blood Pressure)	+	-	-	+	-	-
Bellikci-Koyu 2022 (Energy Intake)	+	-	-	-	-	-
Bendtsen 2018 (Anthopometry: BW, BMI, WC)	+	-	-	+	-	-
Bendtsen 2018 (Blood markers: LDL-C, HDL-C, TG, FG)	+	-	-	+	-	-
Bendtsen 2018 (Systolic Blood Pressure)	+	-	-	+	-	-
Bendtsen 2018 (Energy intake)	+	-	-	-	-	-
Campbell 1999 (Anthopometry: BW)	-	-	+	+	-	-
Campbell 1999 (Energy Intake)	-	-	+	-	-	-
Chen 2019 (Anthopometry: BMI, FM,WC)	+	-	+	+	-	-
Chen 2019 (Blood markers: LDL-C, HDL-C, TG, FG)	+	-	+	+	-	-
Chen 2019 (Systolic Blood Pressure)	+	-	+	+	-	-
Chen 2019 (Energy Intake)	+	-	+	-	-	-
Engel 2018 (Anthopometry: BW, BMI, FM)	-	-	+	+	-	-
Engel 2018 (Blood markers: LDL-C, HDL-C, TG, FG)	-	-	+	+	-	-
Engel 2018 (Systolic Blood Pressure)	-	-	+	+	-	-
Engel 2018 (Energy Intake)	-	-	+	-	-	-
Gunther 2005 (Anthopometry: BW, BMI, FM)	-	-	+	+	-	-
Gunther 2005 (Energy Intake)	-	-	+	-	-	-
Harvey-Berino 2005 (Anthopometry: BW, FM)	+	-	+	+	-	-
Harvey-Berino 2005 (Energy Intake)	+	-	+	-	-	-
Raziani 2016 (Anthopometry: BW, BMI, WC)	-	+	+	+	+	-
Raziani 2016 (Anthopometry: FM)	-	+	+	+	-	-
Raziani 2016 (Blood markers: LDL-C, HDL-C, TG, FG)	-	+	+	+	+	-
Raziani 2016 (Systolic Blood Pressure)	-	+	+	+	+	-
Raziani 2016 (Energy Intake)	-	-	-	-	-	-
Schmidt 2021 (Anthopometry: BW, WC, FM)	-	+	+	+	-	-
Schmidt 2021 (Blood markers: LDL-C, HDL-C, TG, FG, HbA1c)	-	+	+	+	-	-
Schmidt 2021 (Systolic Blood Pressure)	-	+	+	+	-	-
Schmidt 2021 (Energy Intake)	-	-	+	-	-	-

Continues on next page

Tanaka 2014 (Anthopometry: BW, WC, FM)	-	-	+	+	-	-
Tanaka 2014 (Blood markers: LDL-C, HDL-C, TG, FG, HbA1c)	-	-	+	+	-	-
Tanaka 2014 (Systolic Blood Pressure)	-	-	+	+	-	-
Tanaka 2014 (Energy Intake)	-	-	+	-	-	-
Thomas 2010 (Anthopometry: BW, WC, FM)	+	-	+	+	-	-
Thomas 2010 (Energy Intake)	+	-	+	-	-	-
Thompson 2005 (Anthopometry: BW, WC, FM)	+	-	-	+	-	-
Thompson 2005 (Blood markers: LDL-C, HDL-C, TG, FG)	+	-	-	+	-	-
Thompson 2005 (Energy Intake)	+	-	-	-	-	-
van Loan 2011 (Anthopometry: BW, FM, WC)	-	-	-	+	-	-
van Loan 2011 Anthopometry: BMI)	-	-	+	+	-	-
van Loan 2011 (Blood markers: LDL-C, HDL-C, FG)	-	-	-	+	-	-
van Loan 2011 (Blood markers: TG)	-	-	+	+	-	-
Wennersberg 2009 (Anthopometry: BW, BMI, FM, WC)	-	-	+	+	-	-
Wennersberg 2009 (Blood markers: LDL-C, HDL-C, TG, FG)	-	-	+	+	-	-
Wennersberg 2009 (Blood markers: Hba1c)	-	-	X	+	-	X
Wennersberg 2009 (Systolic Blood Pressure)	-	-	+	+	-	-
Wennersberg 2009 (Energy Intake)	-	-	-	-	-	-
Zemel 2004 (Anthopometry: BW, WC, FM)	-	-	X	+	-	X
Zemel 2004 (Blood markers: LDL-C, HDL-C, TG)	-	-	X	+	-	X
Zemel 2004 (Systolic Blood Pressure)	-	-	X	+	-	X
Zemel 2005 (P1) (Anthopometry: BW, WC, FM)	X	-	+	+	-	X
Zemel 2005 (P1) (Blood markers: LDL-C, HDL-C, TG)	X	-	+	+	-	X
Zemel 2005 (P1) (Systolic Blood Pressure)	X	-	+	+	-	X
Zemel 2005 (P1) (Energy Intake)	X	-	+	-	-	X
Zemel 2005 (P2) (Anthopometry: BW, WC, FM)	-	-	+	+	-	-
Zemel 2005 (P2) (Systolic Blood Pressure)	-	-	+	+	-	-
Zemel 2005 (P2) (Energy Intake)	-	-	+	-	-	-
Zemel 2009 (Anthopometry: BW, WC, FM)	-	-	-	+	-	-
Zemel 2009 (Systolic Blood Pressure)	-	-	-	+	-	-
Zemel 2009 (Energy Intake)	-	-	-	-	-	-

D1: Bias arising from the randomization process
D2: Bias due to deviations from intended interventions
D3: Bias due to missing outcome data
D4: Bias in measurement of the outcome
D5: Bias in selection of the reported result

Judgement
X High
- Some concerns
+ Low

Supplemental Figure 1: Summary of the risk of bias assessment with RoB2 for the included RCTs with parallel design (n=18); Abbreviations: BMI Body Mass Index, BW Body Weight, FG Fasting Glucose, FM Fat Mass, HbA1c Glycated Hemoglobin, HDL-C High-Density Lipoprotein Cholesterol, LDL-C Low-Density Lipoprotein Cholesterol, TG Triacylglycerol, SBP Systolic Blood Pressure, WC Waist Circumference.

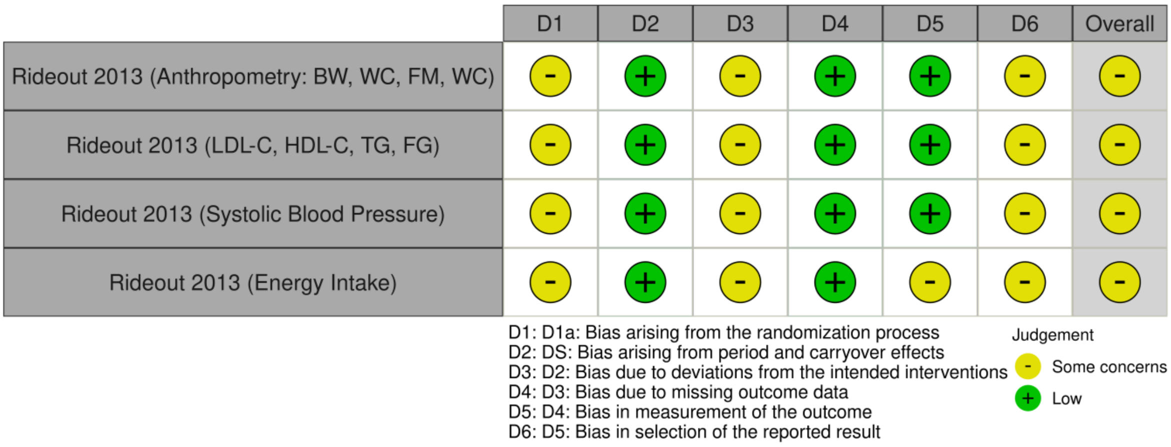

Supplemental Figure 2: Summary of the risk of bias assessment with RoB2 for the included RCT with cross-over design; Abbreviations: BMI Body Mass Index, BW Body Weight, FG Fasting Glucose, FM Fat Mass, HDL-C High-Density Lipoprotein Cholesterol, LDL-C Low-Density Lipoprotein Cholesterol, TG Triacylglycerol, WC Waist Circumference.

A) Body weight (kg; n studies: 16; n participants: 1042)

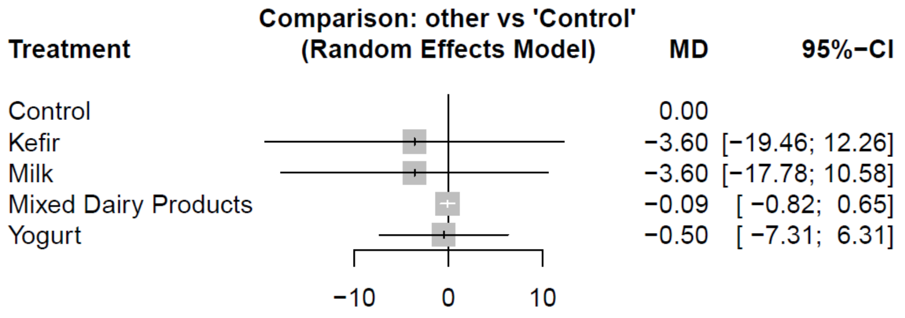

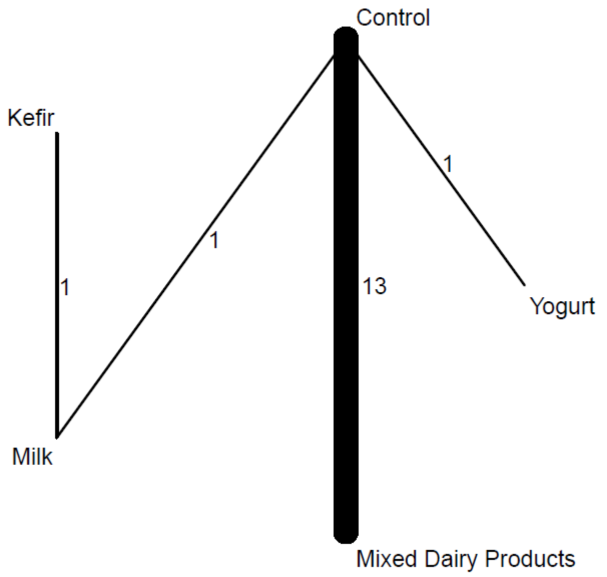

Assessment of inconsistency: design-by-treatment interaction random effects model: NA

B) Body mass index (kg/m²; n studies: 7; n participants: 558)

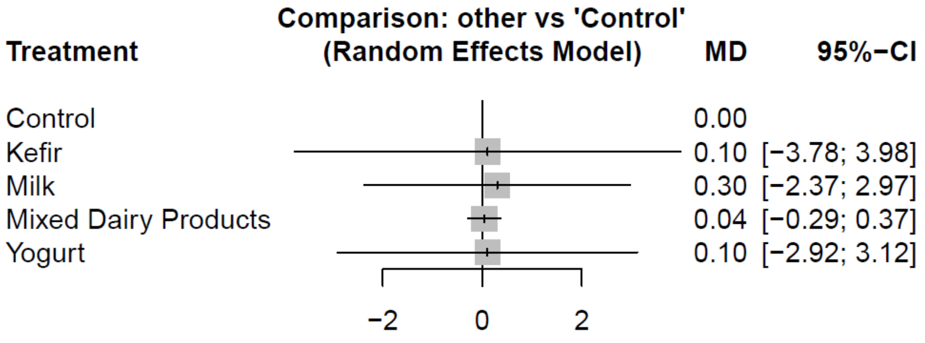

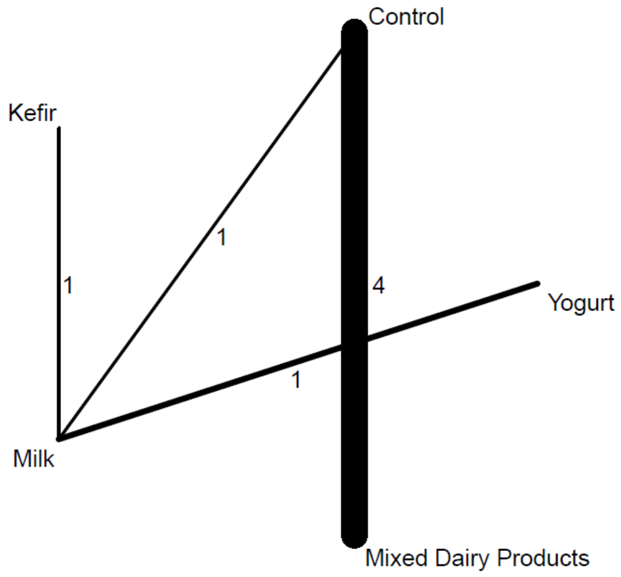

Assessment of inconsistency: design-by-treatment interaction random effects model: NA

C) Fat mass (kg; n studies: 15; n participants: 1063)

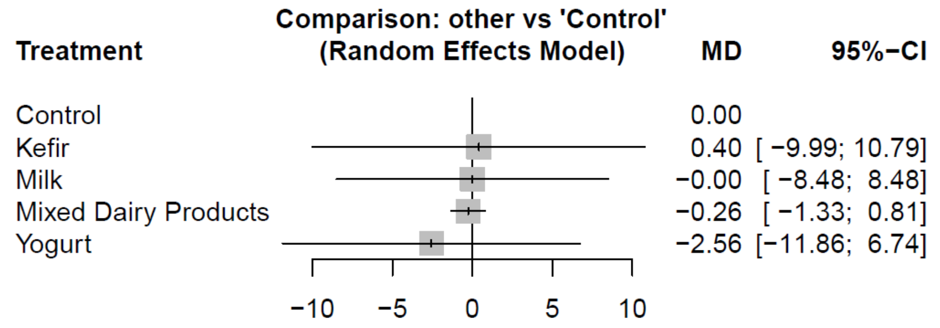

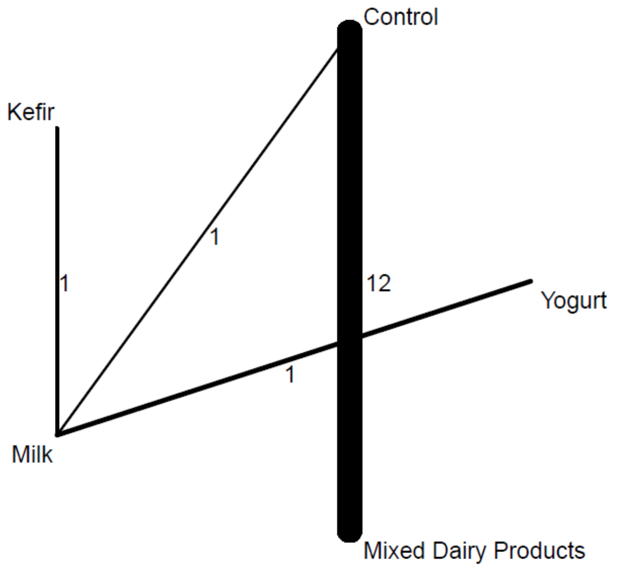

Assessment of inconsistency: design-by-treatment interaction random effects model: NA

D) LDL-C (mmol/l; n studies: 10; n participants: 754)

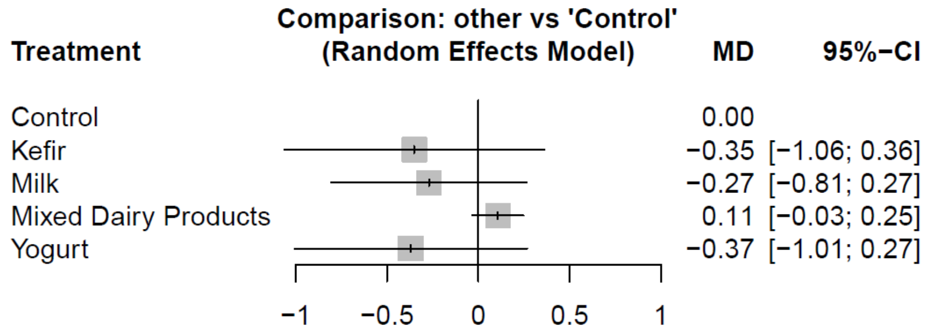

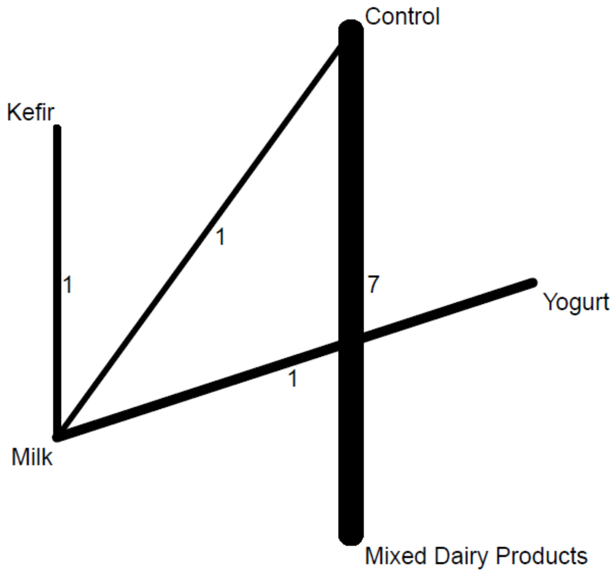

Assessment of inconsistency: design-by-treatment interaction random effects model: NA

E) HDL-C (mmol/l; n studies: 10; n participants: 754)

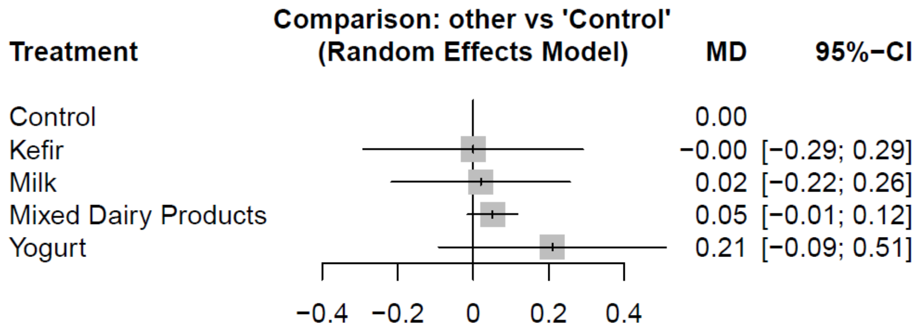

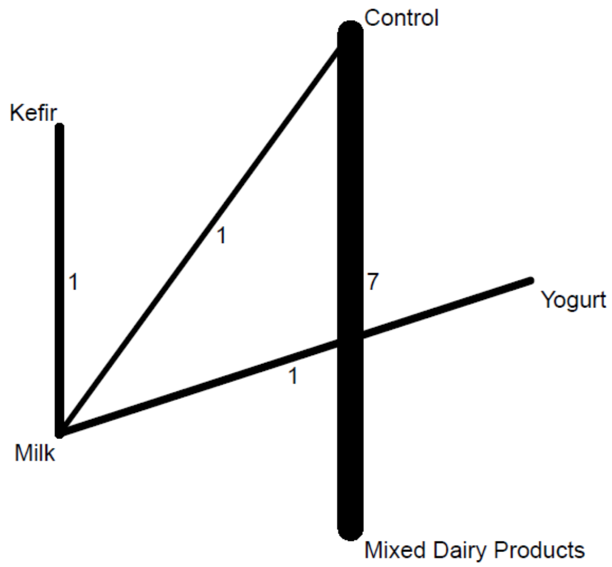

Assessment of inconsistency: design-by-treatment interaction random effects model: NA

F) Triglycerides (mmol/l; n studies: 10; n participants: 748)

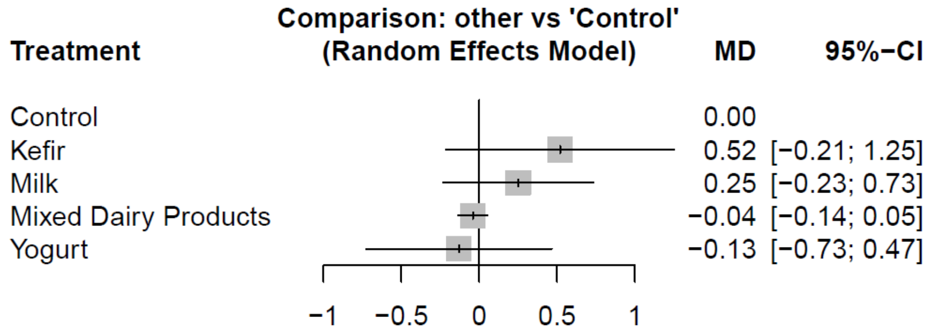

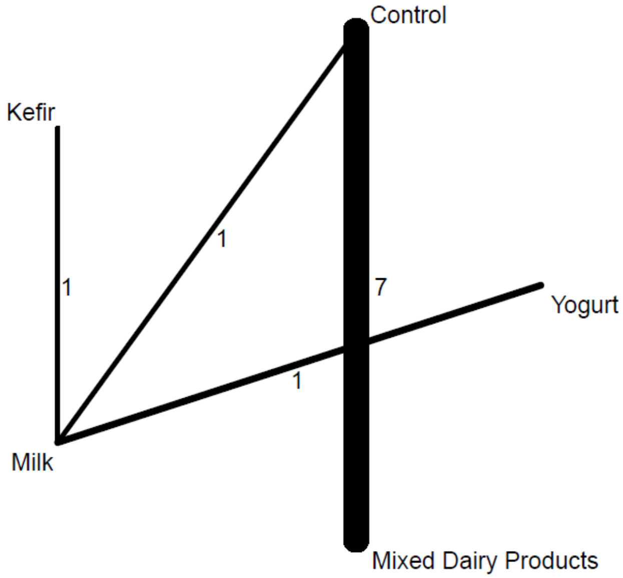

G)

Assessment of inconsistency: design-by-treatment interaction random effects model: NA

G) Fasting glucose (mmol/l; n studies: 9; n participants: 743)

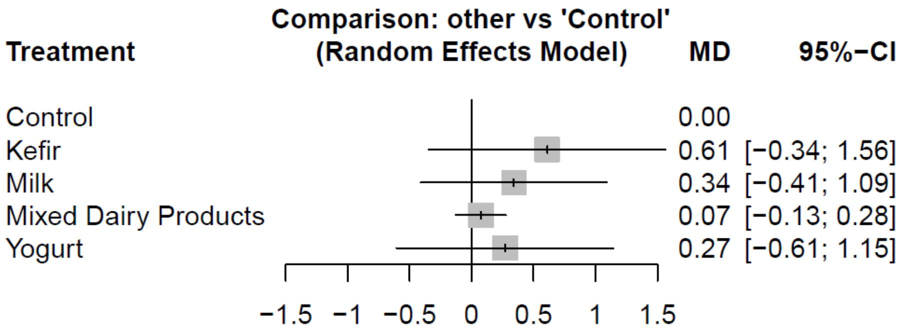

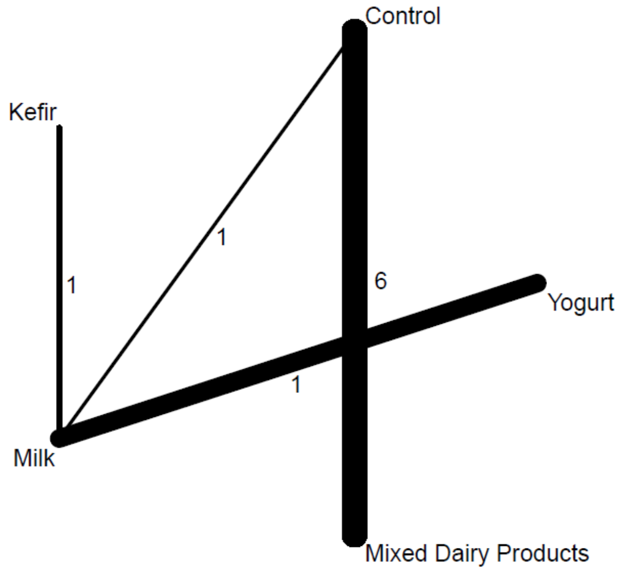

Assessment of inconsistency: design-by-treatment interaction random effects model: NA

H) Systolic blood pressure (mmHg; n studies: 9; n participants: 726)

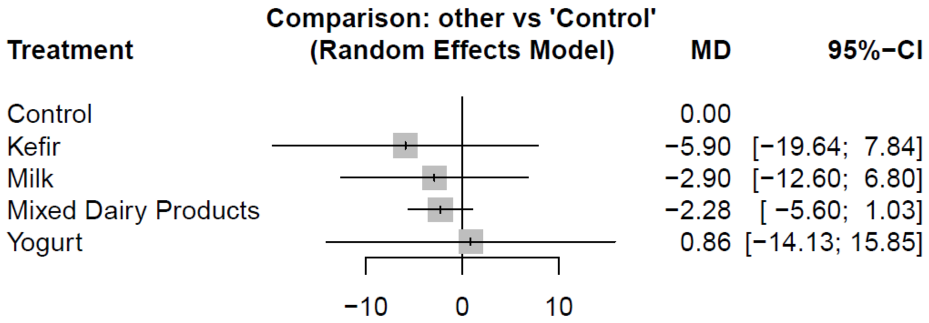

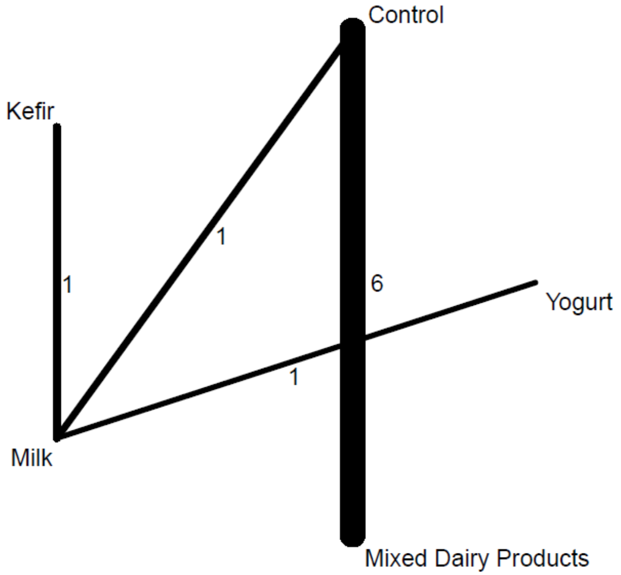

Assessment of inconsistency: design-by-treatment interaction random effects model: NA

I) Energy intake (kcal/d; n studies: 15; n participants: 1014)

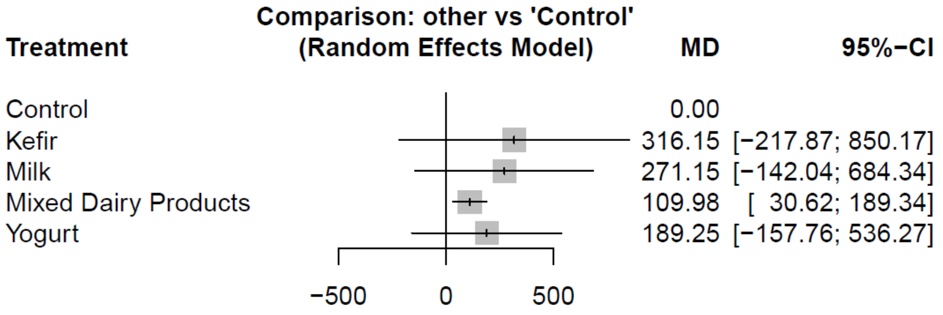

Assessment of inconsistency: design-by-treatment interaction random effects model: p=0.30

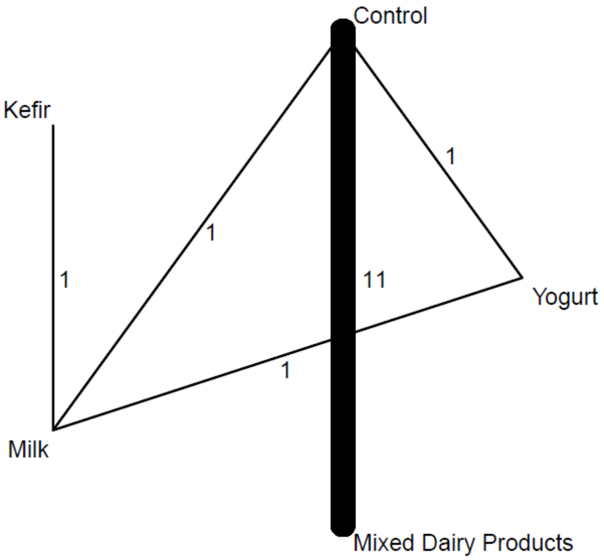

Supplemental Figure 3: Forest plots summarizing mean differences with 95% confidence intervals and network plots for (A) body weight (B), body mass index (C) fat mass (D) LDL-C, (E) HDL-C, (F) triglycerides, (G) fasting glucose, (H) systolic blood pressure and (I) energy intake as estimated from the network meta-analysis on dairy product intake.

MD: CI: Confidence Interval; Mean Difference; NA Not Applicable

Network plots: line width: weight from random effects model comparing two treatments; numbers: number of studies directly comparing treatments

A) Waist circumference (cm; n studies: 13; n participants: 870)

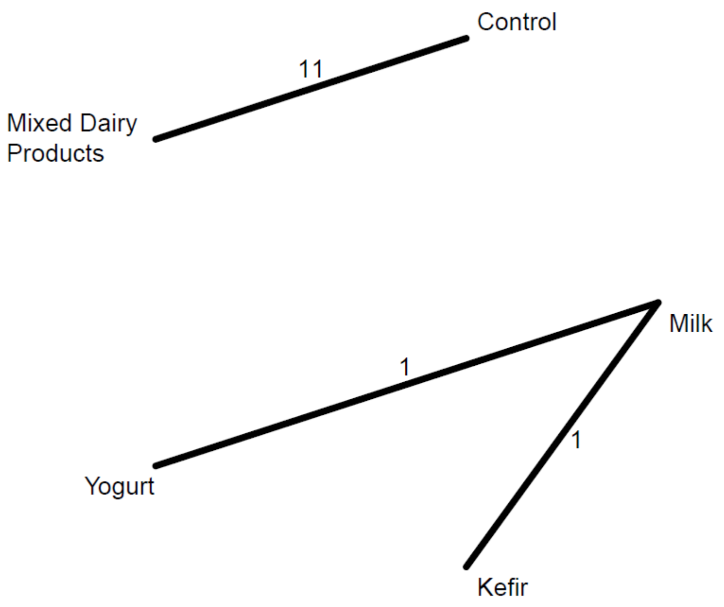

B) Glycated hemoglobin (%; n studies: 4; n participants: 405)

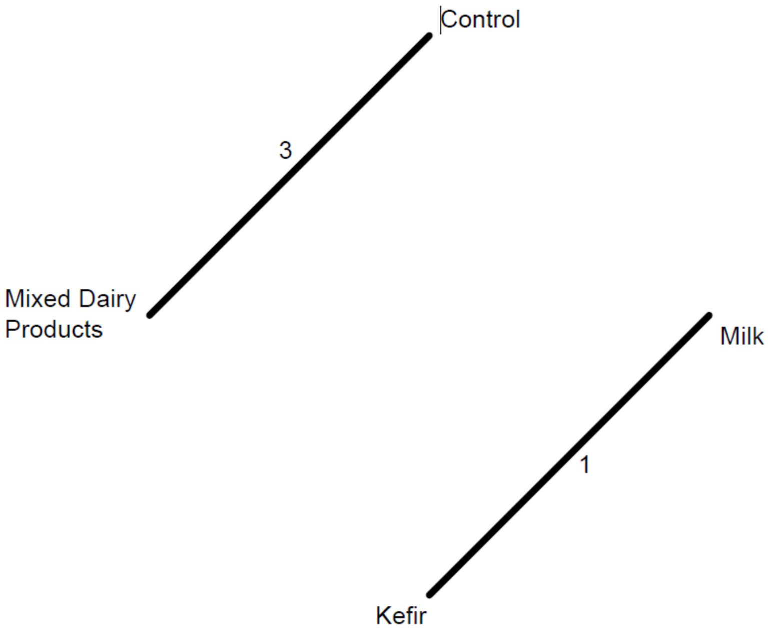

Supplemental Figure 4: Disconnected network plots for outcomes (A) waist circumference and (B) glycated hemoglobin and interventions on dairy product intake
Numbers: number of studies directly comparing treatments

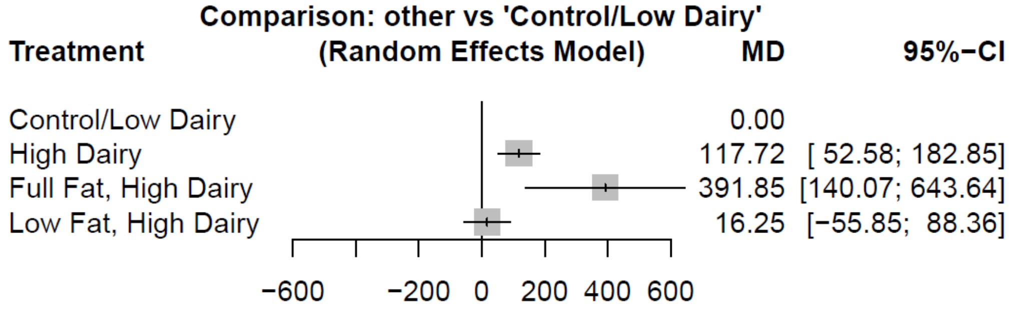

Assessment of inconsistency: design-by-treatment interaction random effects model: p=0.40

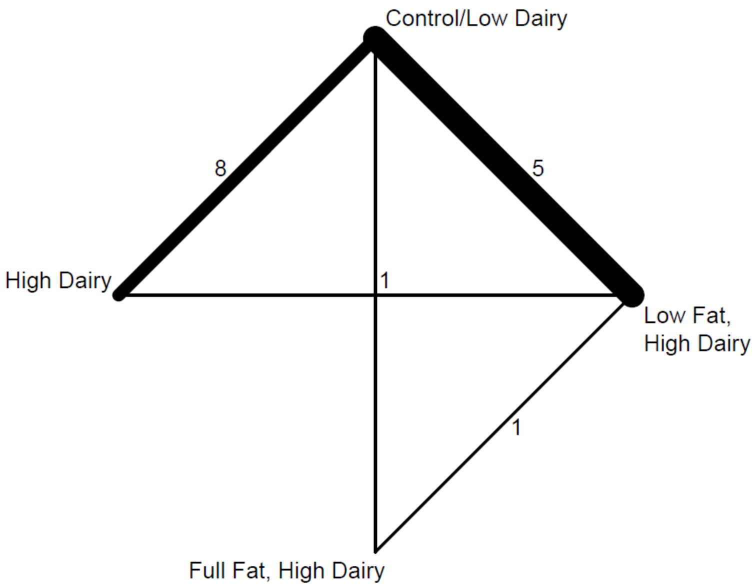

Supplemental Figure 5: Forest plot summarizing mean differences with 95% confidence interval and network plot for energy intake (kcal/d; n studies:14; n participants: 945) as estimated from the network meta-analysis on total dairy intake with a combined control/low dairy group

MD: Mean Difference; CI: Confidence Interval

Control/low dairy: usual diet or a diet with 0-2 dairy servings/day or an equal amount in grams/day; high dairy: ≥3 dairy servings/day or an equal amount in grams/day; full fat dairy: dairy products with its natural fat content; low fat dairy: skimmed or semi-skimmed dairy products

Network plots: line width: weight from random effects model comparing two treatments; numbers: number of studies directly comparing treatments

A) Body weight

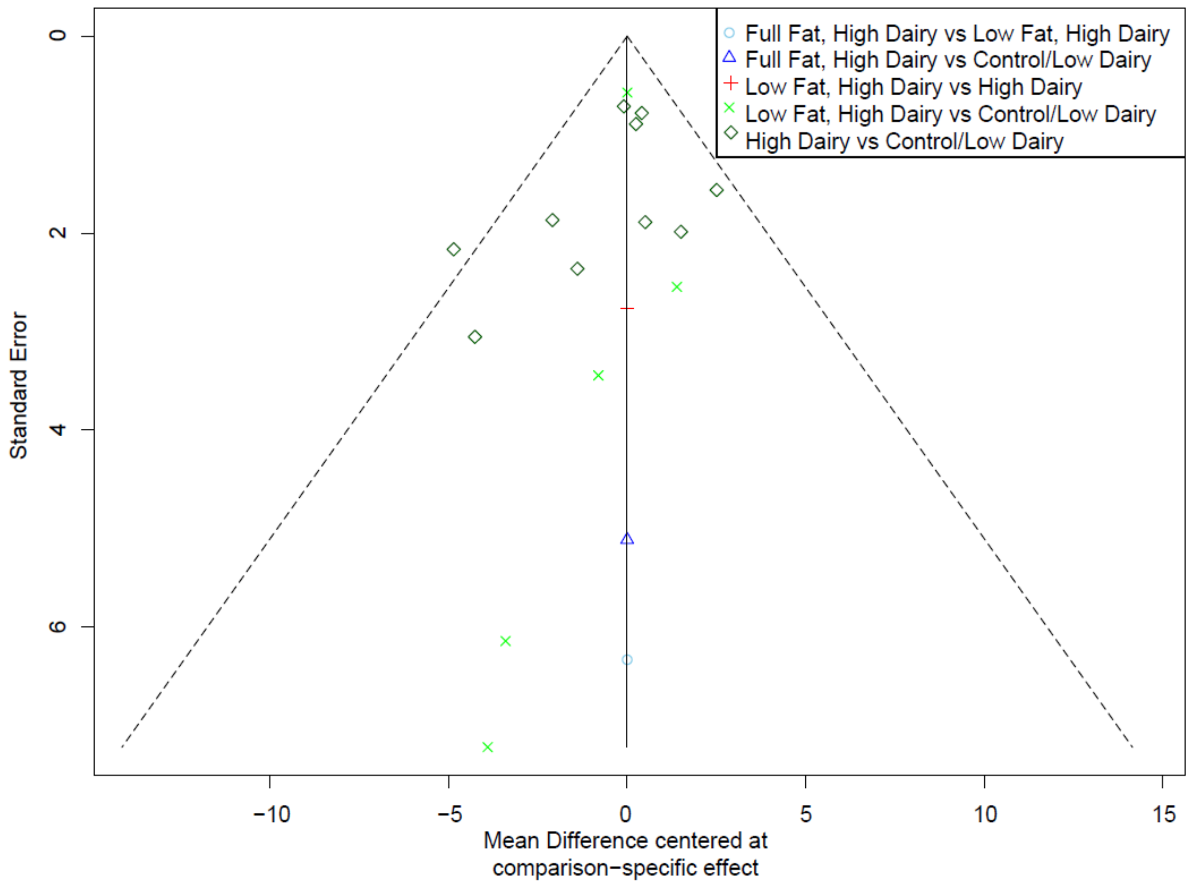

B) Fat mass

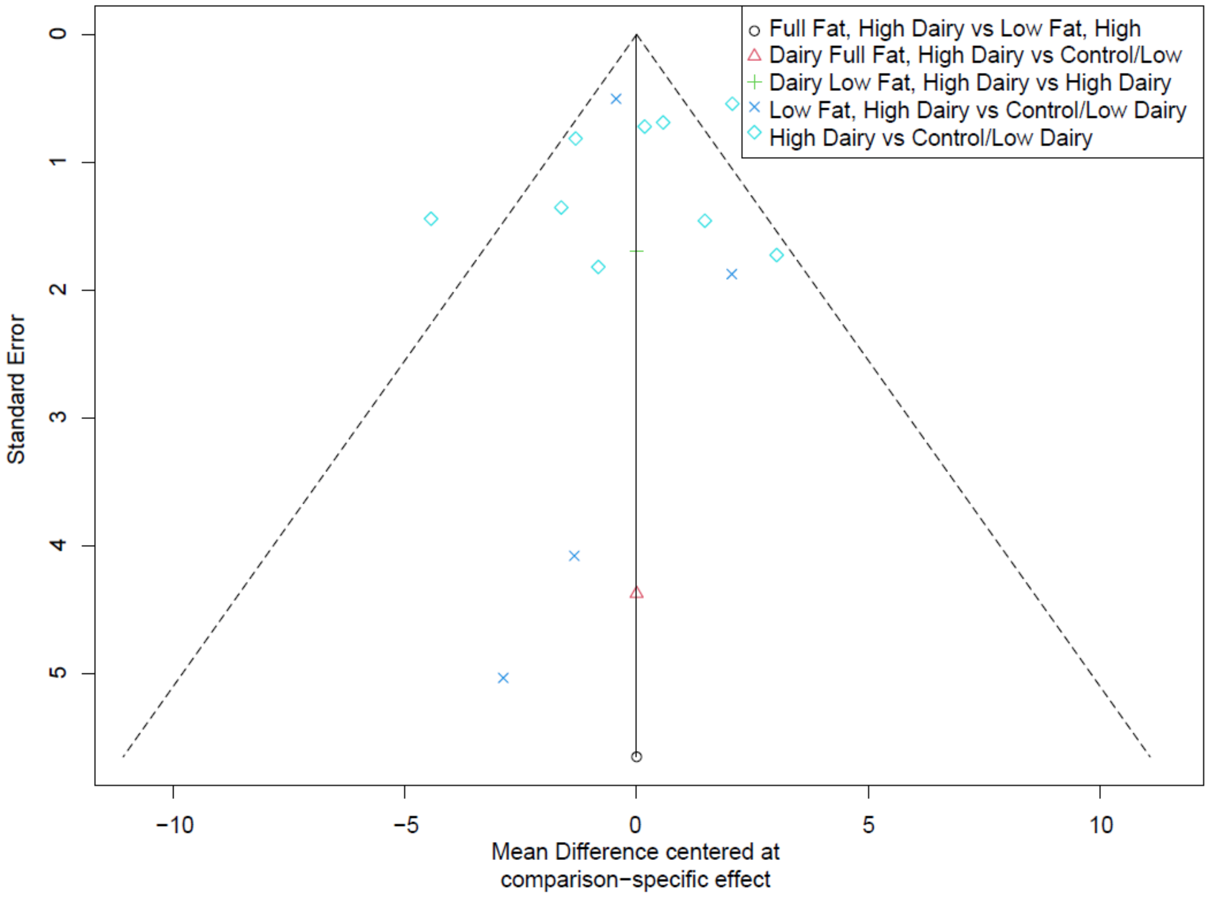

C) Waist circumference

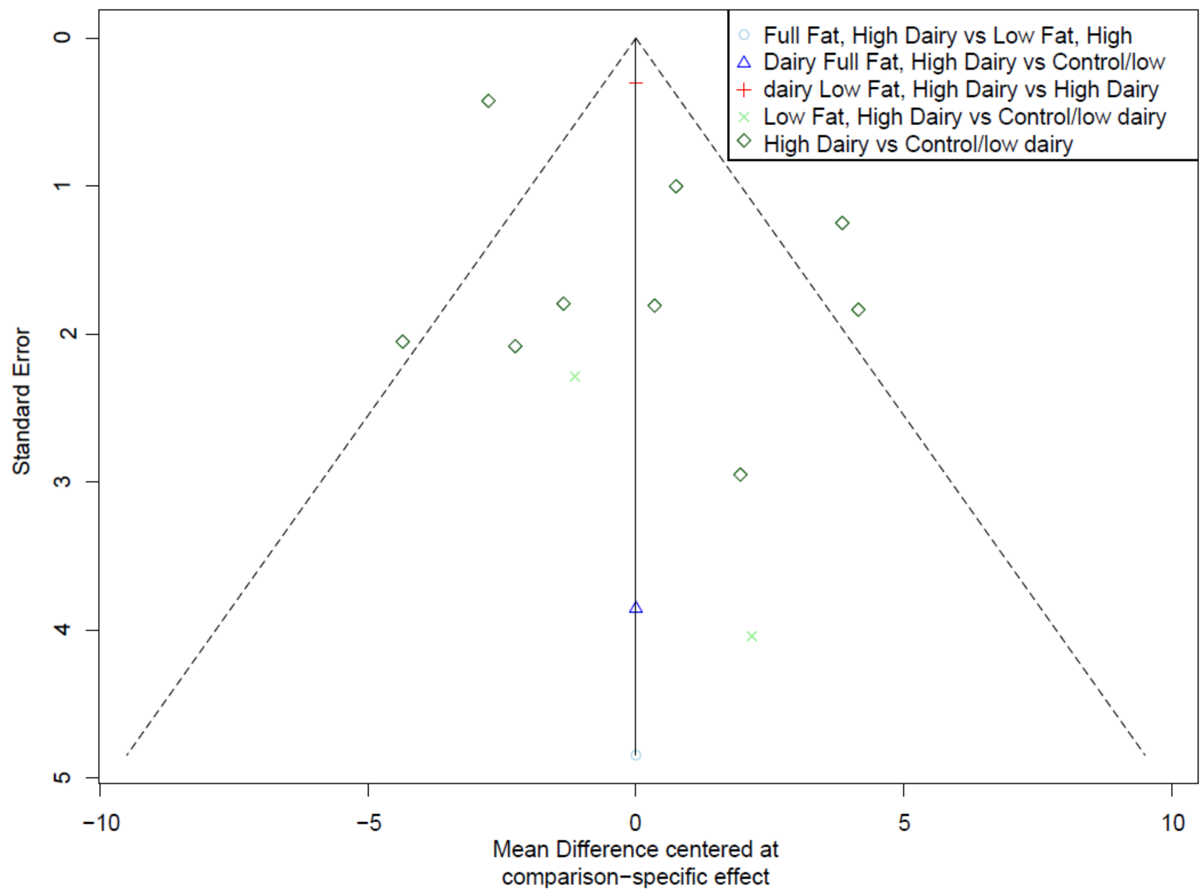

D) Energy intake

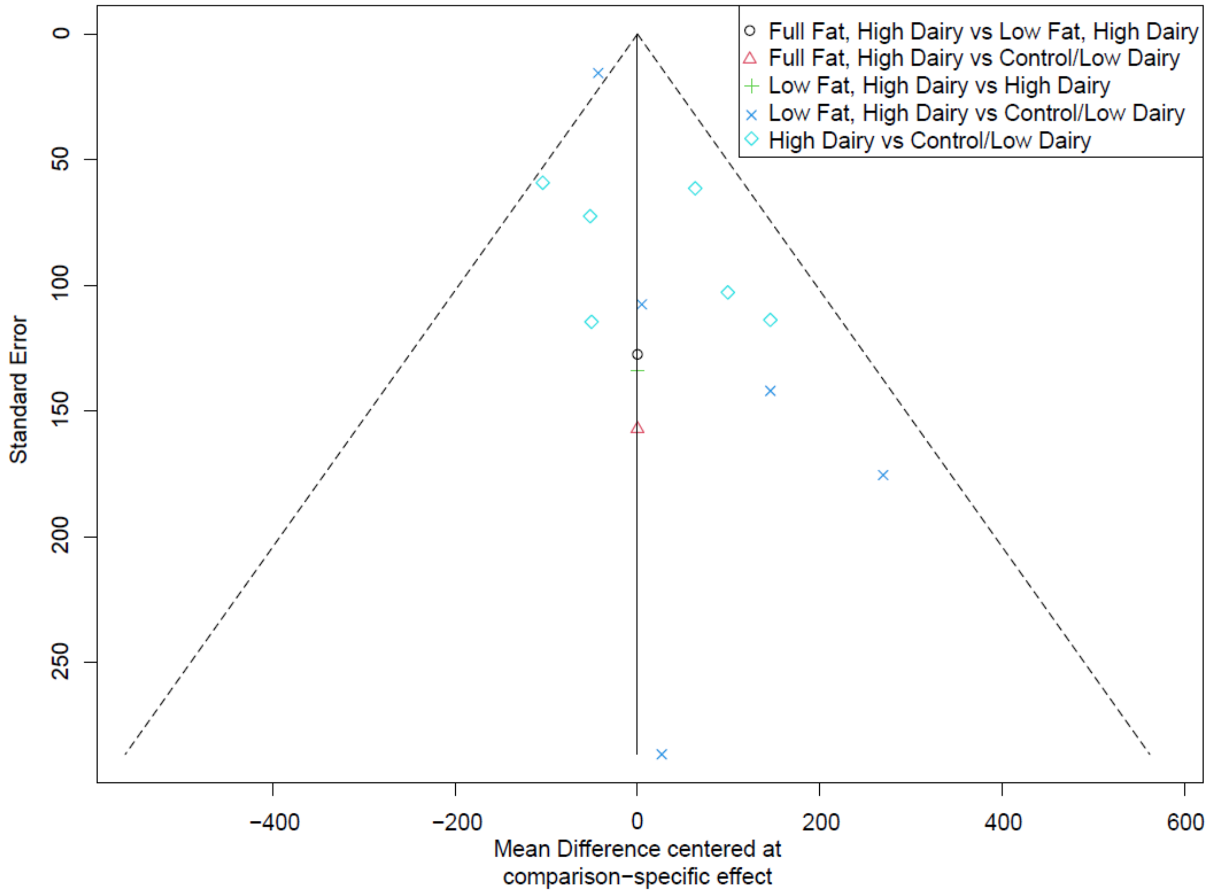

Supplementary Figure 6: Comparison adjusted funnel plot for (A) body weight, (B) fat mass, (C) waist circumference and (D) energy intake for the network meta-analysis on total dairy intake with a combined control/low dairy group

Control/low dairy: usual diet or a diet with 0-2 dairy servings/day or an equal amount in grams/day; high dairy: ≥3 dairy servings/day or an equal amount in grams/day; full fat dairy: dairy products with its natural fat content; low fat dairy: skimmed or semi-skimmed dairy products

A) Body weight

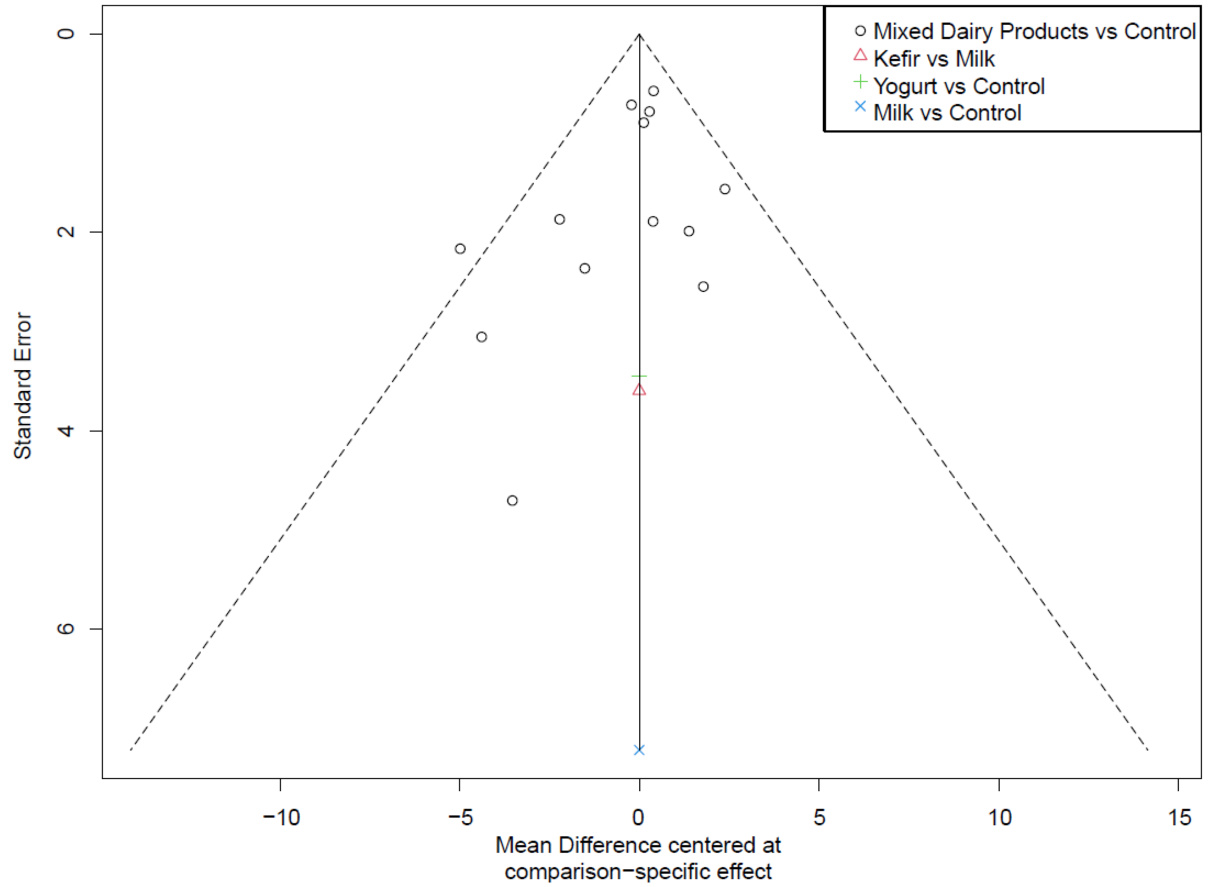

B) Fat mass

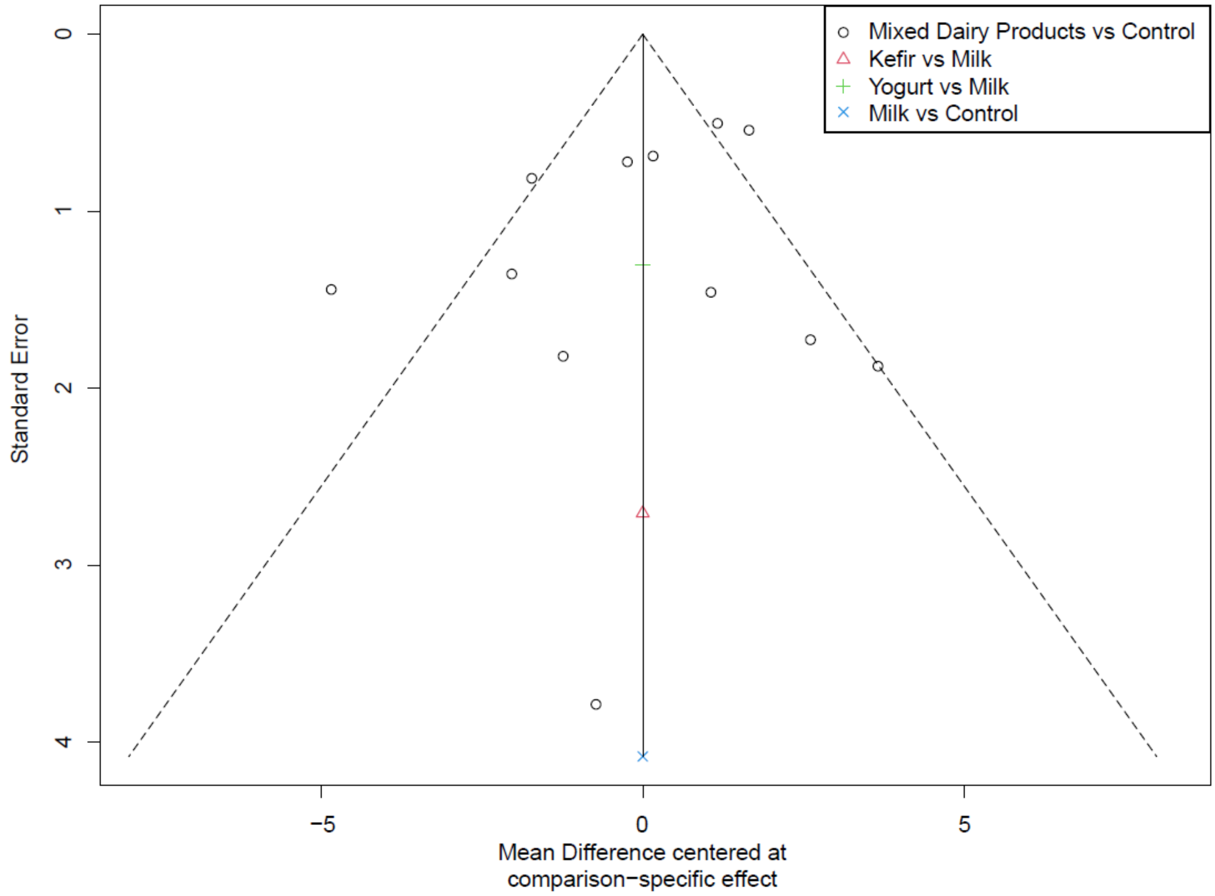

C) LDL-C

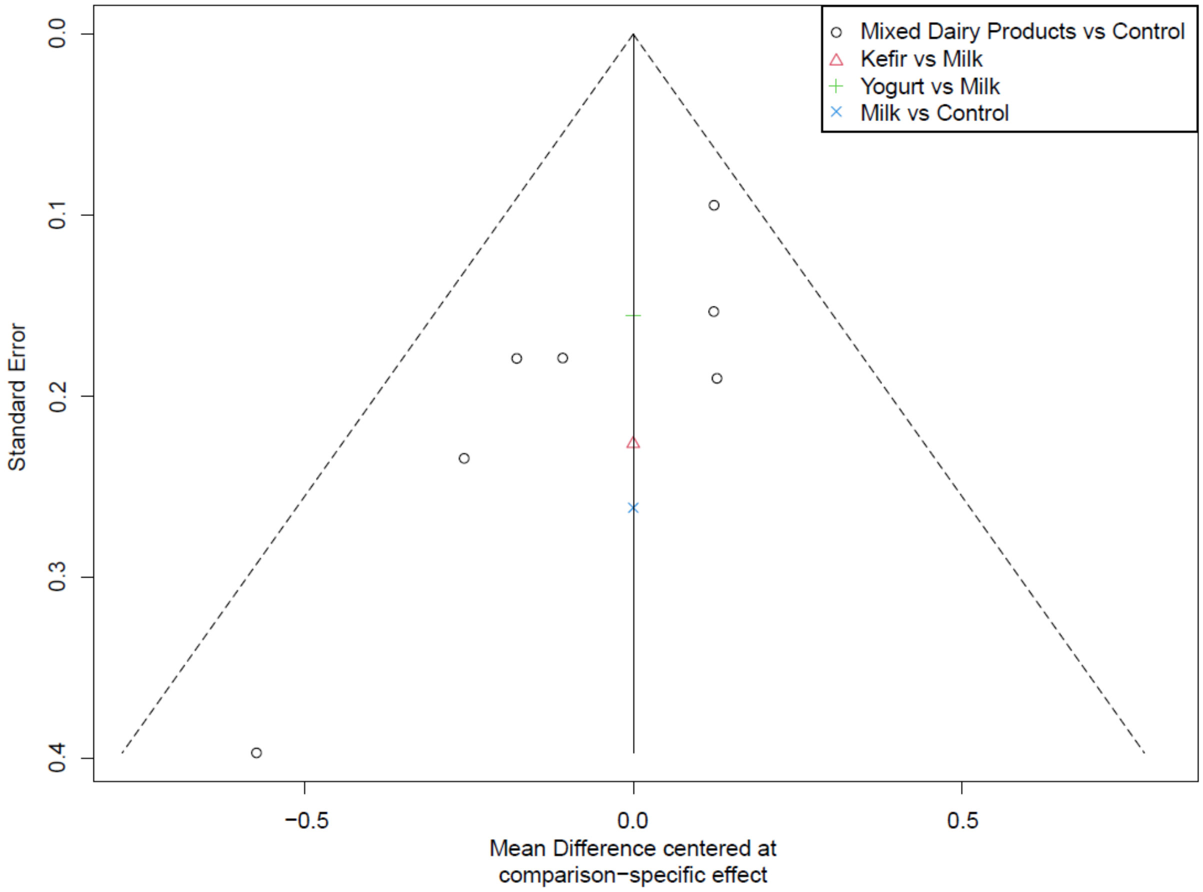
Mixed Dairy Products vs Control
Kefir vs Milk
Yogurt vs Milk
Milk vs Control
Standard Error
0.0
0.1
0.2
0.3
0.4
−0.5
0.0
0.5
Mean Difference centered at
comparison−specific effect

D) HDL-C

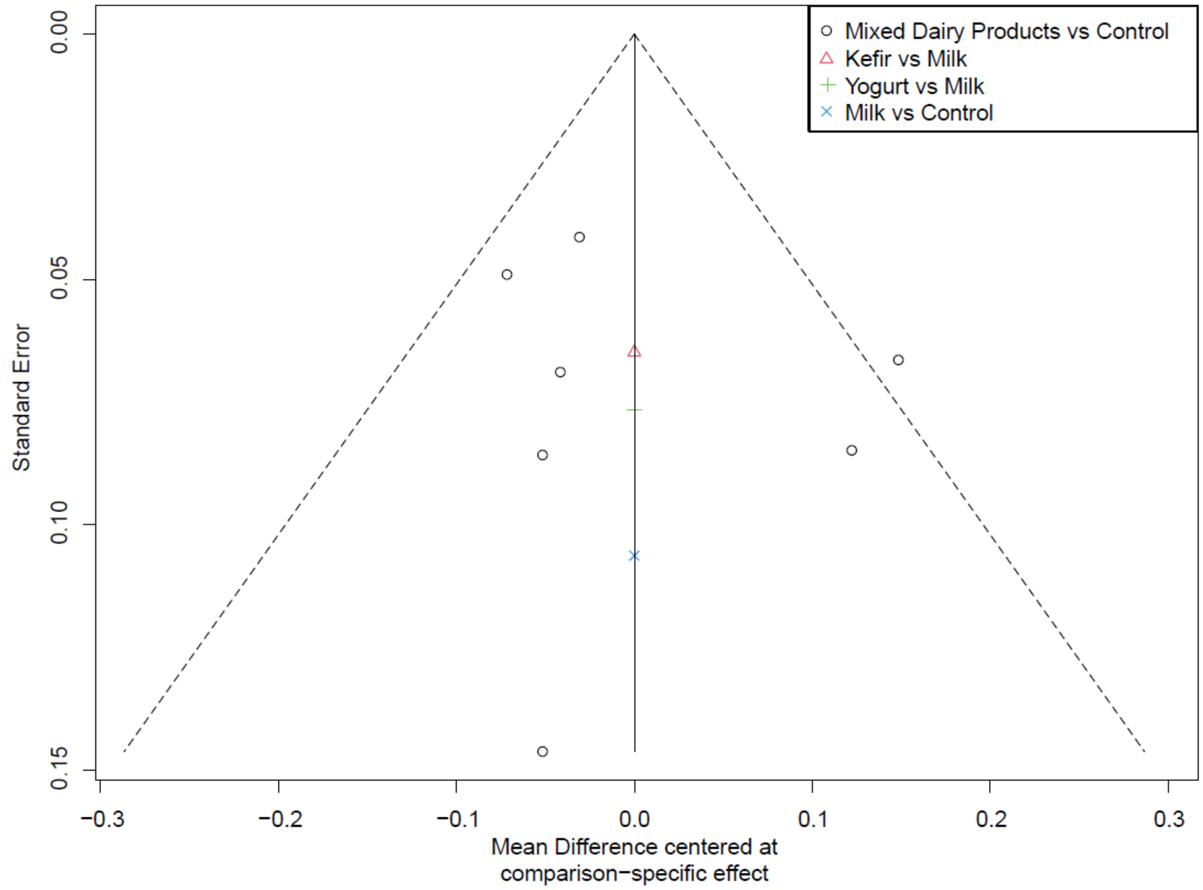
Mixed Dairy Products vs Control
Kefir vs Milk
Yogurt vs Milk
Milk vs Control
Standard Error
0.00
0.05
0.10
0.15
−0.3
−0.2
−0.1
0.0
0.1
0.2
0.3
Mean Difference centered at
comparison−specific effect

E) Triglycerides

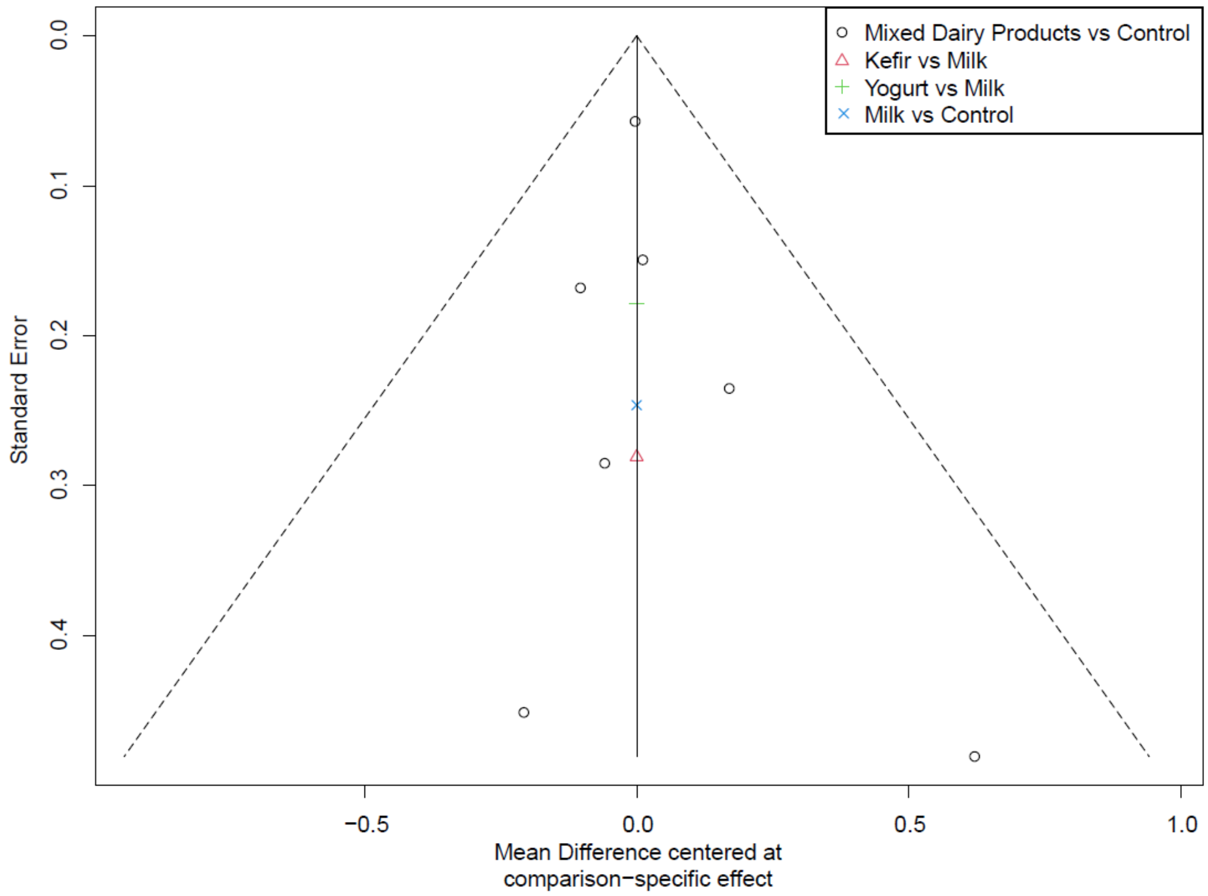

F) Energy intake

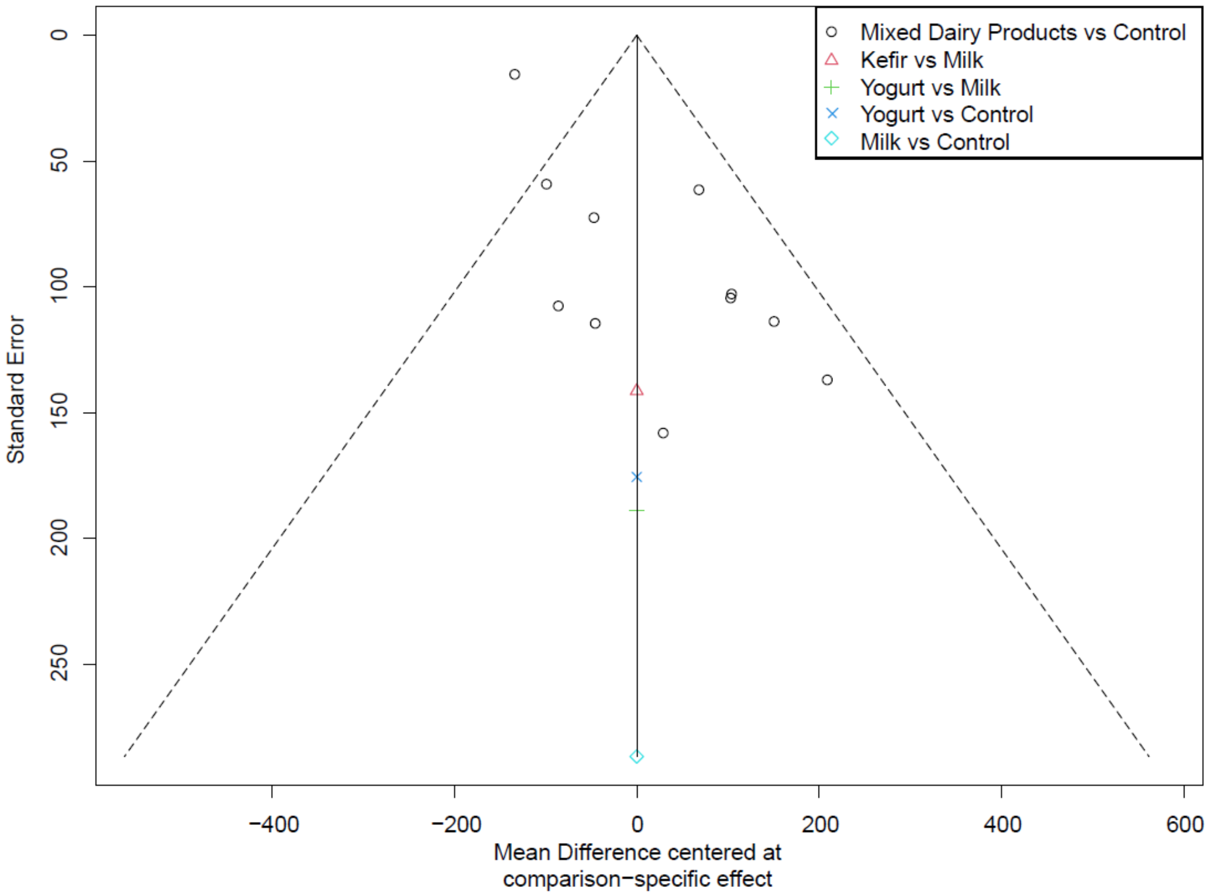

Supplemental Figure 7: Comparison adjusted funnel plot for (A) body weight, (B) fat mass, (C) LDL-C, (D) HDL-C, (E) triglycerides and (F) energy intake for the network meta-analysis on dairy product intake

A) Body weight (kg; n studies: 16; n participants: 1081)

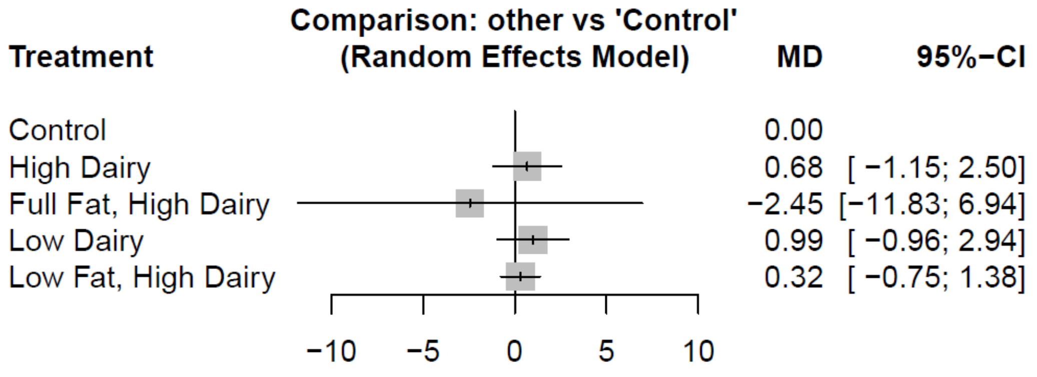

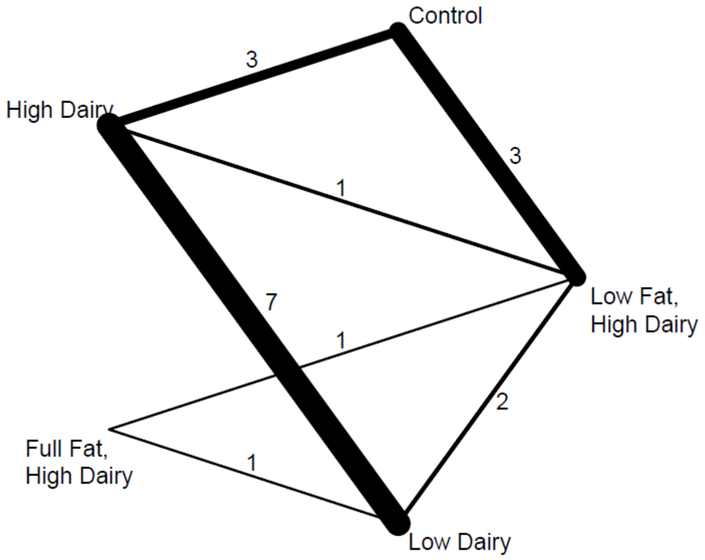

Assessment of inconsistency: design-by-treatment interaction random effects model: p=0.74

B) Body mass index (kg/m²; n studies: 6; n participants: 497)

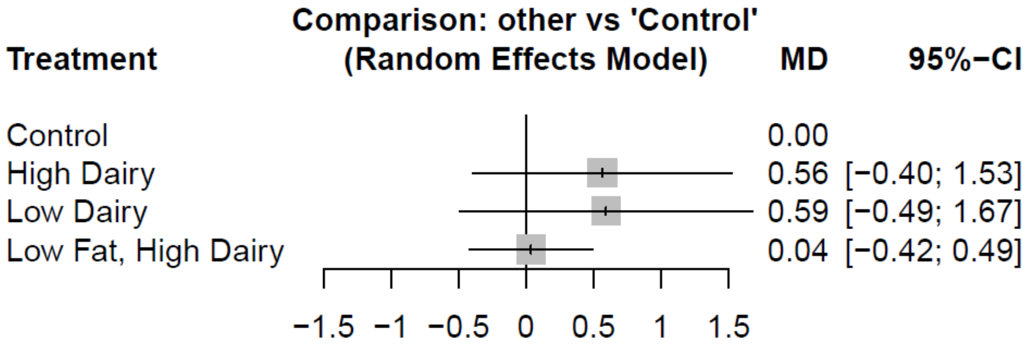

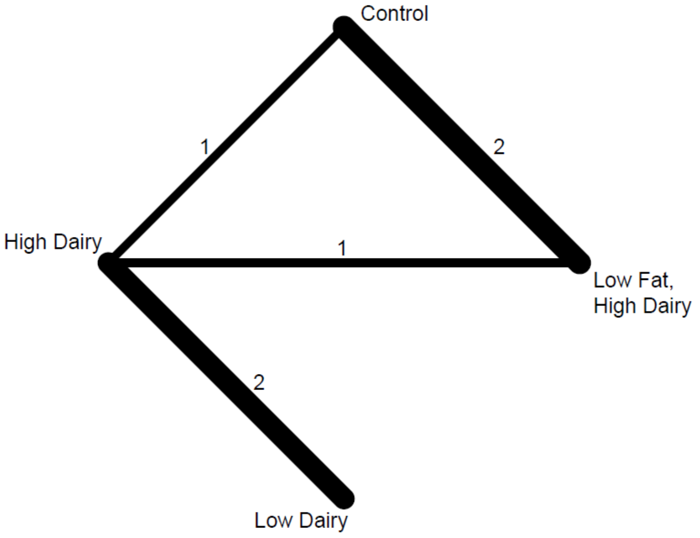

Assessment of inconsistency: design-by-treatment interaction random effects model: p=0.18

C) Fat mas (kg¸ n studies: 14; n participants: 1002)

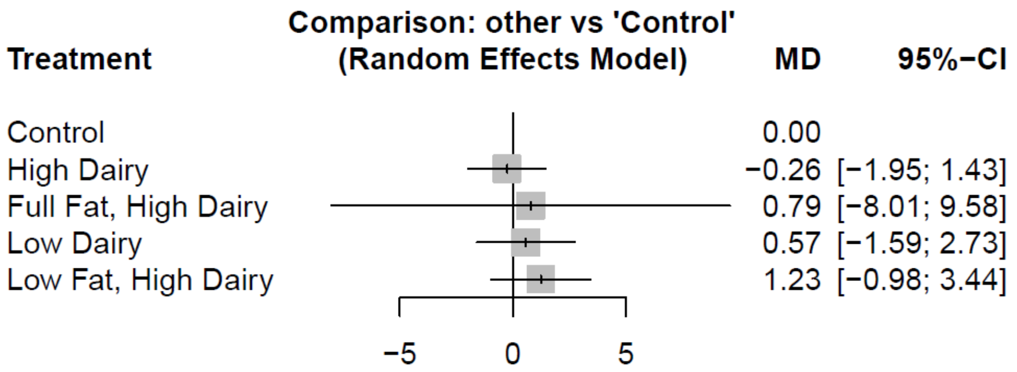

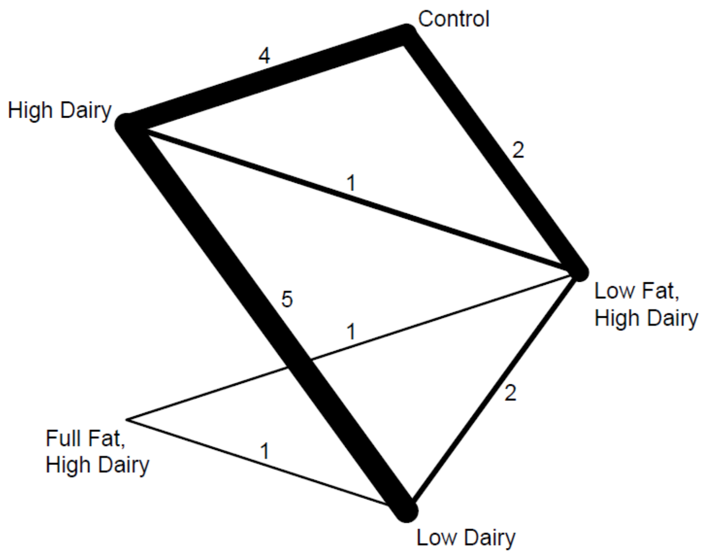

Assessment of inconsistency: design-by-treatment interaction random effects model: p=0.60

D) Waist circumference (cm; n studies: 12; n participants: 809)

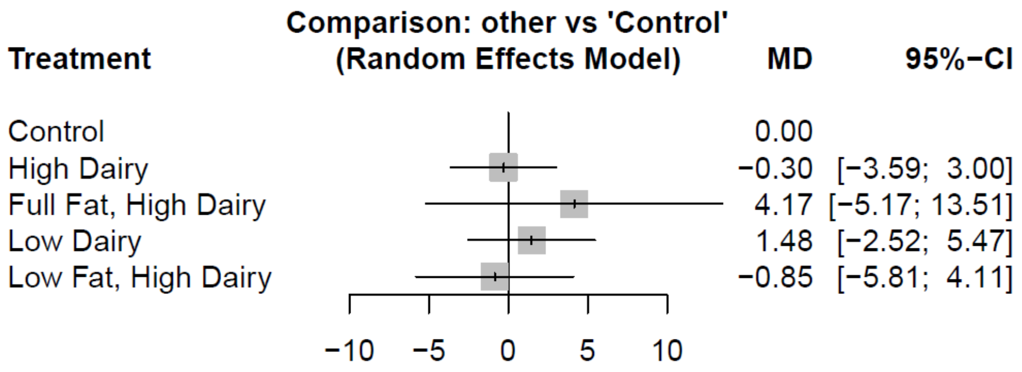

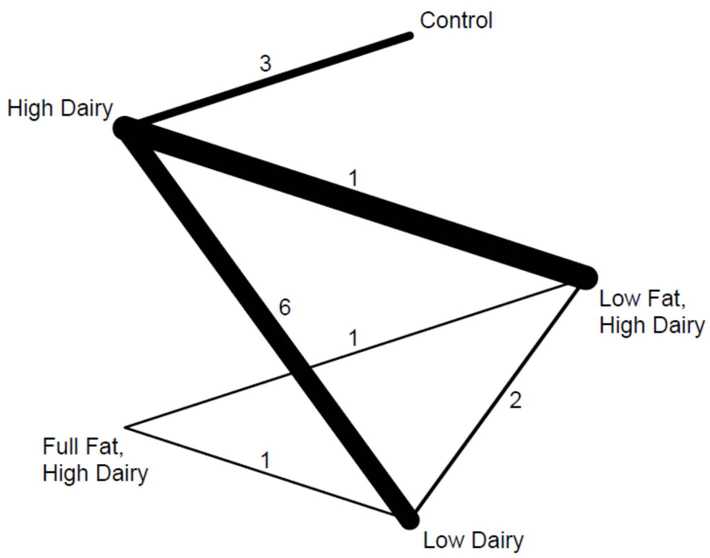

Assessment of inconsistency: design-by-treatment interaction random effects model: p=0.65

E) LDL-C (mmol/L; n studies: 9; n participants: 693)

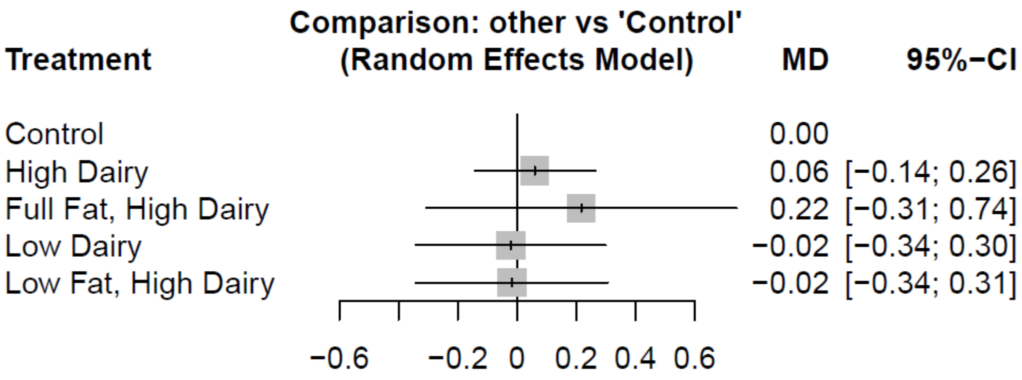

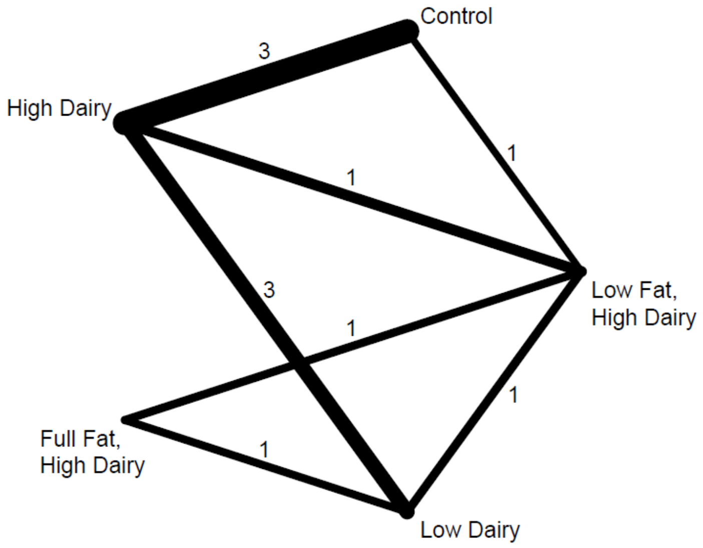

Assessment of inconsistency: design-by-treatment interaction random effects model: p=0.52

F) HDL-C (mmol/L; n studies: 9; n participants: 693)

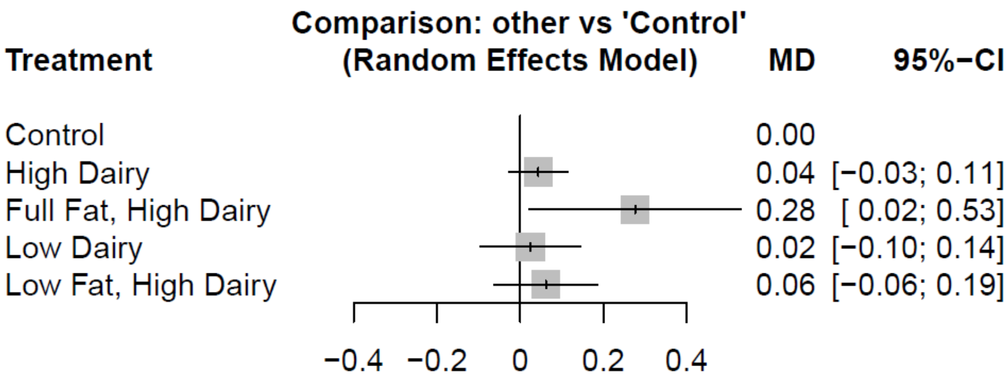

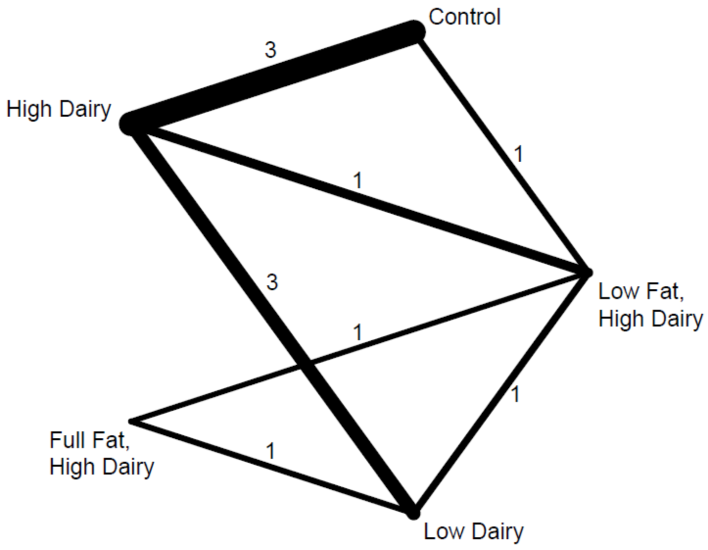

Assessment of inconsistency: design-by-treatment interaction random effects model: p=0.82

G) Triglycerides (mmol/L; n studies: 9; n participants: 687)

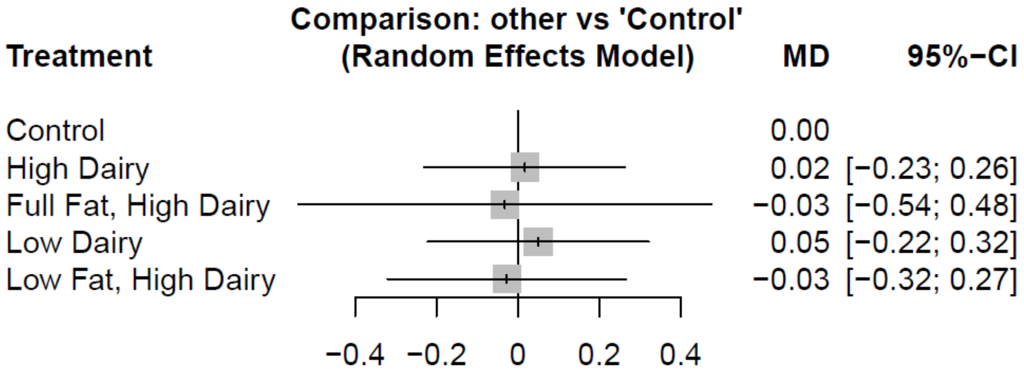

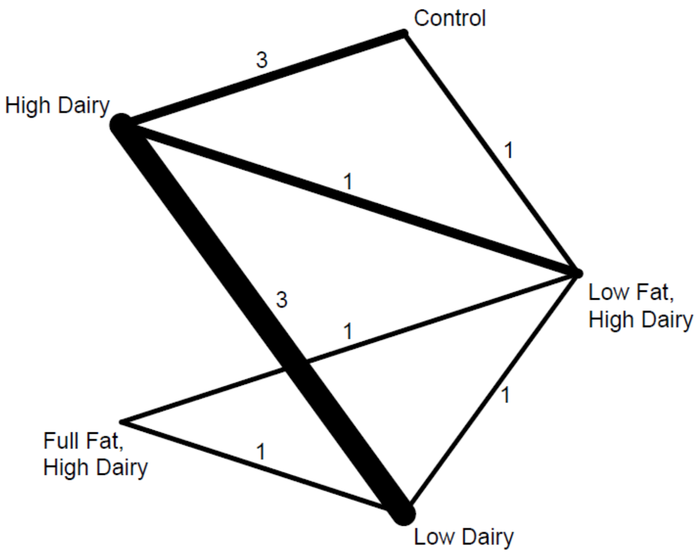

Assessment of inconsistency: design-by-treatment interaction random effects model: p=0.14

H) Fasting glucose (mmol/L; n studies: 8; n participants: 682)

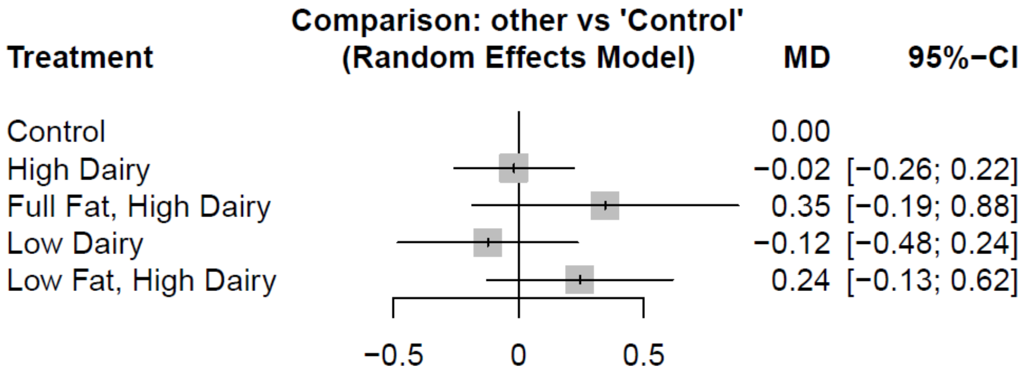

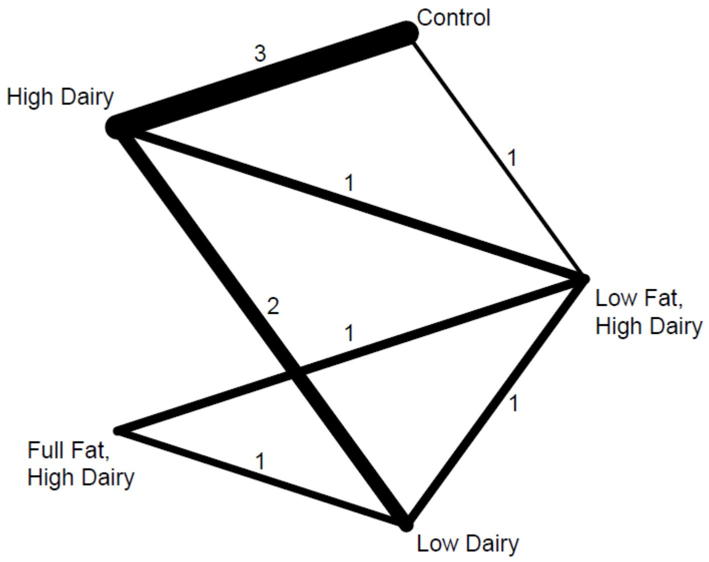

Assessment of inconsistency: design-by-treatment interaction random effects model: p=0.31

I) Systolic blood pressure (mmHg; n studies: 8; n participants: 665)

Comparison: other vs 'Control' (Random Effects Model)

Treatment	MD	95%−CI
Control	0.00	
High Dairy	1.24	[−1.83; 4.31]
Full Fat, High Dairy	−4.15	[−13.30; 4.99]
Low Dairy	4.76	[0.66; 8.86]
Low Fat, High Dairy	−2.71	[−7.23; 1.81]

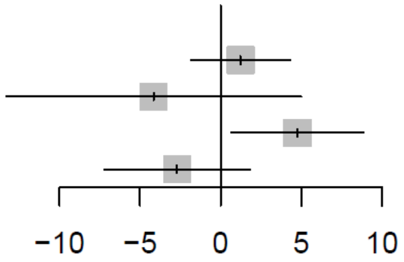

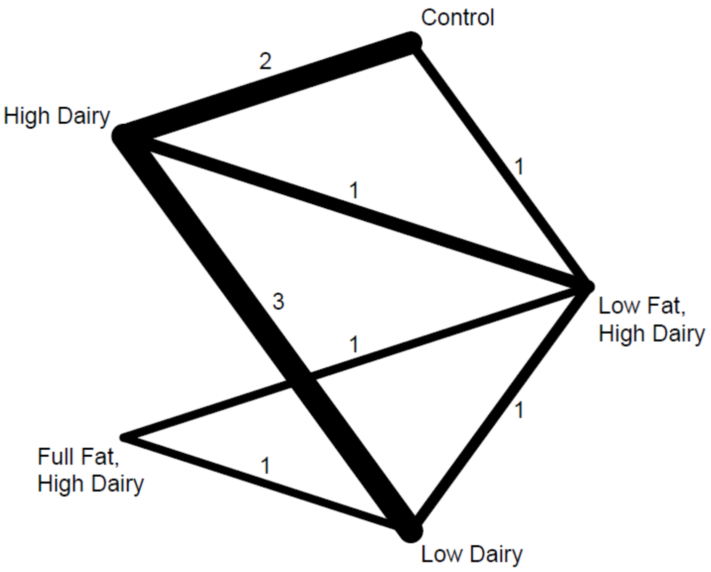

Assessment of inconsistency: design-by-treatment interaction random effects model: p=0.79

J) Energy intake (kcal/d; n studies: 14; n participants: 945)

Comparison: other vs 'Control' (Random Effects Model)

Treatment	MD	95%−CI
Control	0.00	
High Dairy	59.33	[−14.10; 132.75]
Full Fat, High Dairy	331.55	[92.36; 570.74]
Low Dairy	−109.26	[−204.45; −14.06]
Low Fat, High Dairy	−20.66	[−50.46; 9.14]

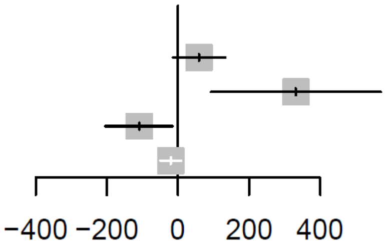

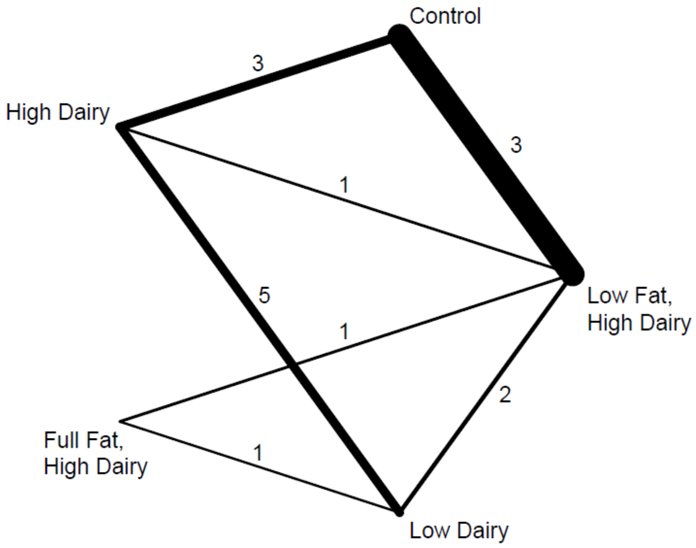

Assessment of inconsistency: design-by-treatment interaction random effects model: p=0.81

Supplemental Figure 8: Forest plots of sensitivity analyses summarizing mean differences with 95% confidence intervals and network plots for (A) body weight (B), body mass index (C) fat mass (D) waist circumference, (E) LDL-C, (F) HDL-C, (G) triglycerides, (H) fasting glucose, (I) systolic blood pressure and (J) energy intake as estimated from the network meta-analysis on total dairy intake with separate control and low dairy groups.
MD: Mean difference; CI: Confidence interval
Control: usual diet; low dairy: a diet with 0-2 dairy servings/day or an equal amount in grams/day; high dairy: ≥3 dairy servings/day or an equal amount in grams/day; full fat dairy: dairy products with its natural fat content; low fat dairy: skimmed or semi-skimmed dairy products
Network plots: line width: weight from random effects model comparing two treatments; numbers: number of studies directly comparing treatments

A) Body weight (kg; n studies: 14; n participants: 1026)

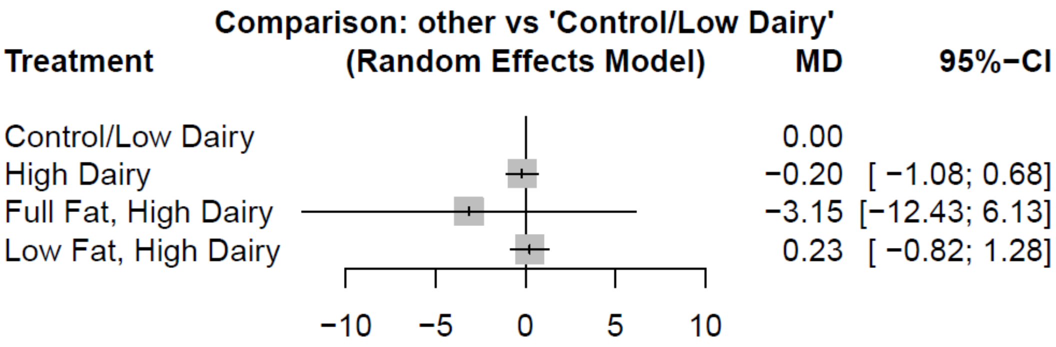

B) Fat mass (kg; n studies: 12; n participants: 947)

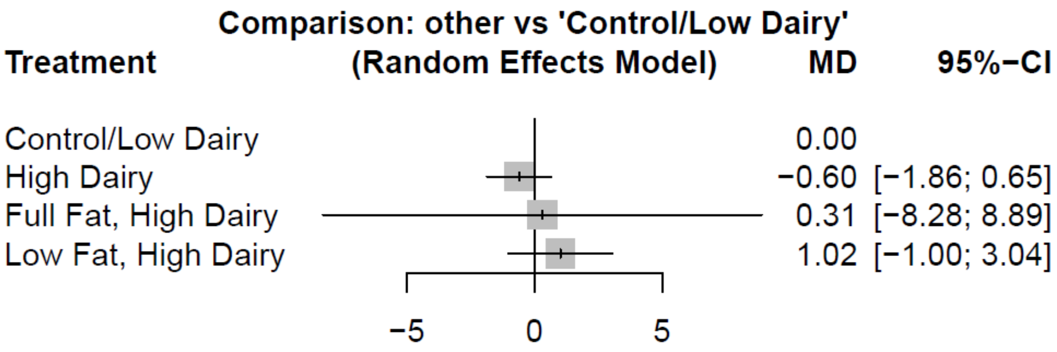

C) Waist Circumference (cm; n studies: 10; n participants: 754)

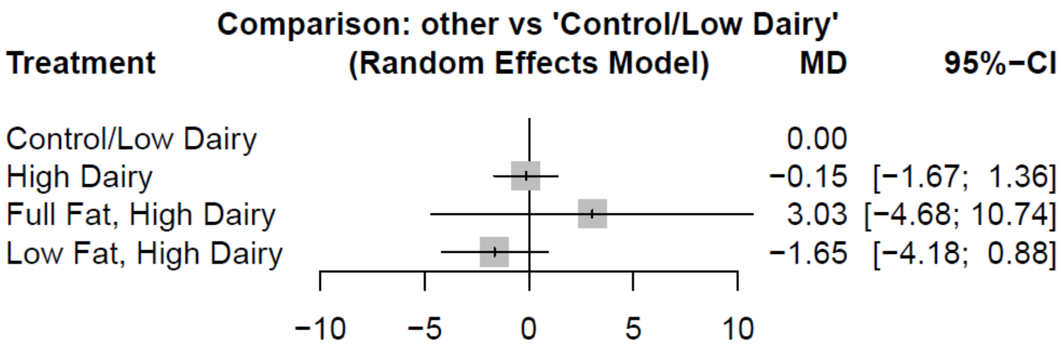

D) LDL-C (mmol/l¸ n studies: 8; n participants: 672)

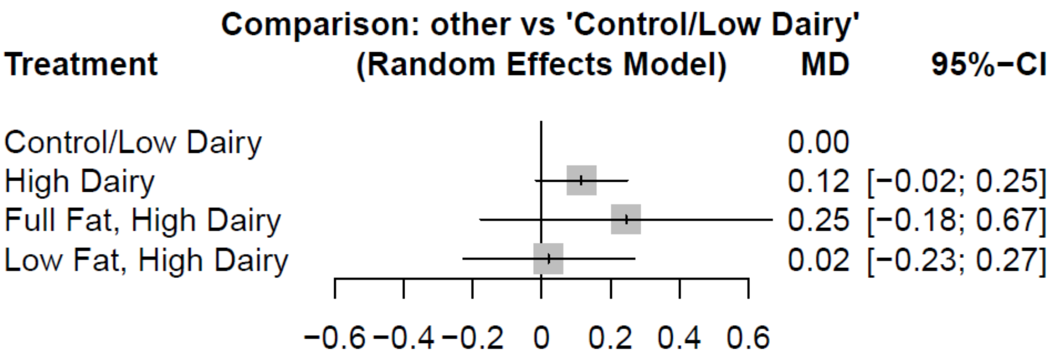

E) HDL-C (mmol/l¸ n studies: 8; n participants: 672)

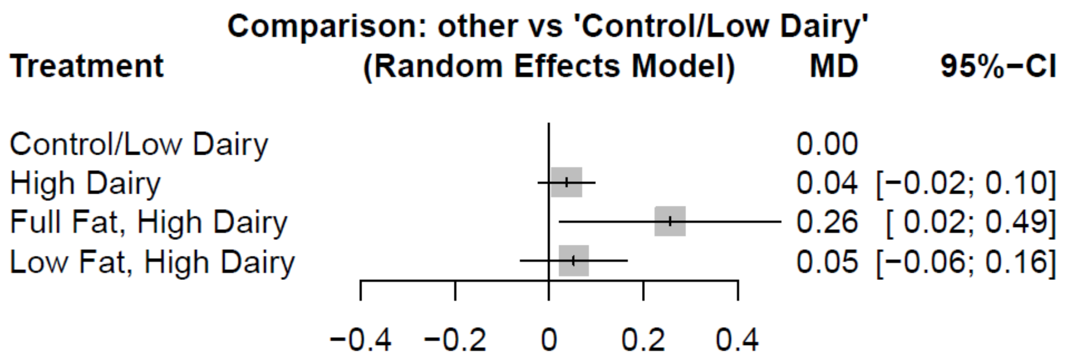

F) Triglycerides (mmol/l¸ n studies: 8; n participants: 666)

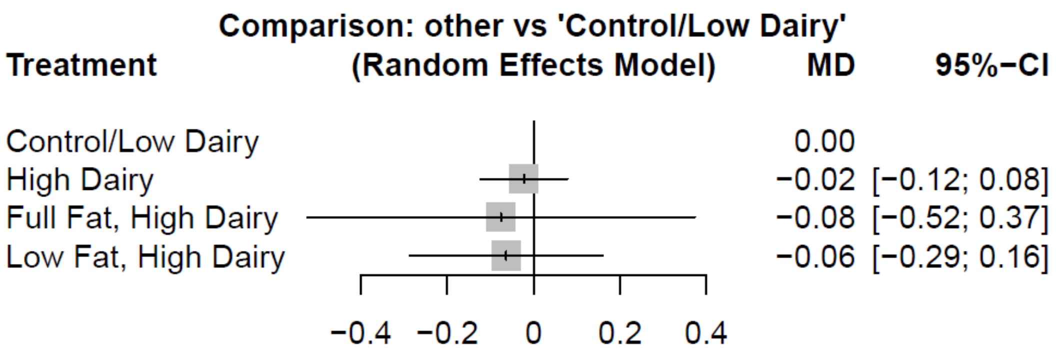

G) Systolic blood pressure (mmHg; n studies: 7; n participants: 631)

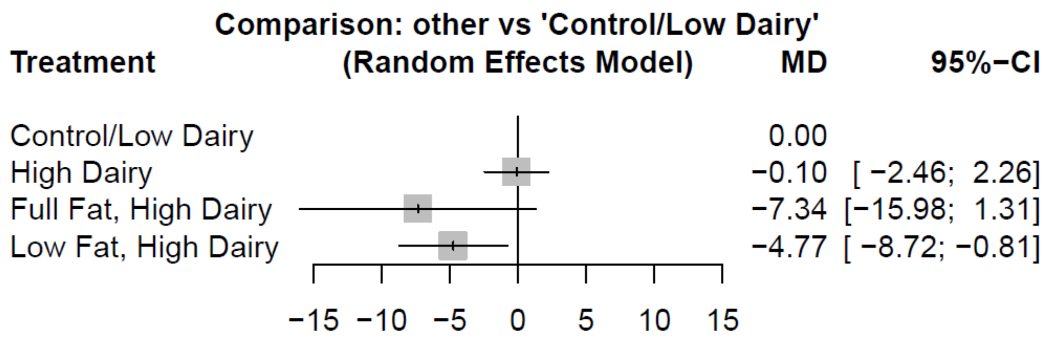

H) Energy intake (kcal/d; n studies: 13; n participants: 911)

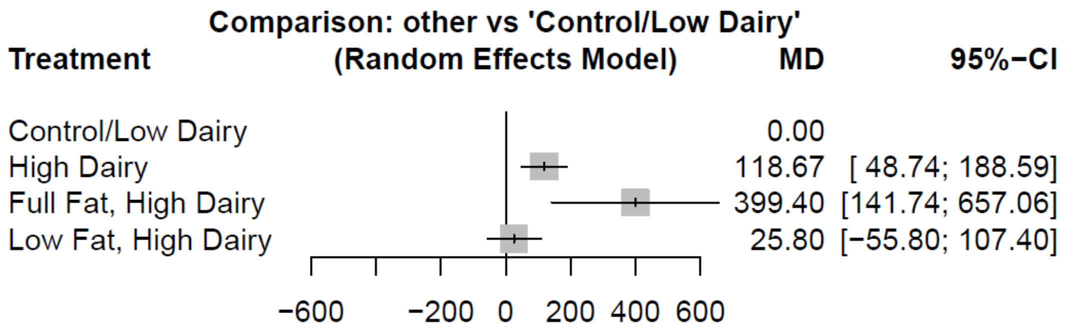

Supplemental Figure 9: Forest plots of sensitivity analyses summarizing mean differences with 95% confidence intervals for (A) body weight, (B) fat mass, (C) waist circumference, (D) LDL-C, (E) HDL-C, (F) triglycerides, (G) systolic blood pressure and (H) energy intake as estimated from the network meta-analysis on total dairy intake with a combined control/low dairy group after excluding studies with high risk of bias
Control/low dairy: usual diet or a diet with 0-2 dairy servings/day or an equal amount in grams/day; high dairy: ≥3 dairy servings/day or an equal amount in grams/day; full fat dairy: dairy products with its natural fat content; low fat dairy: skimmed or semi-skimmed dairy products

Due to a disconnected network for glycated hemoglobin not data are shown

MD: Mean Difference; CI: Confidence Interval

A) Body weight (kg; n studies: 16; n participants: 1081)

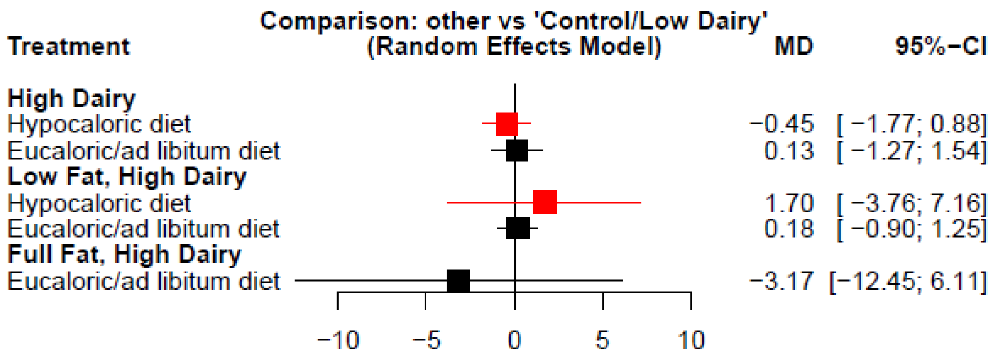

B) Fat mass (kg¸ n studies: 14; n participants: 1002)

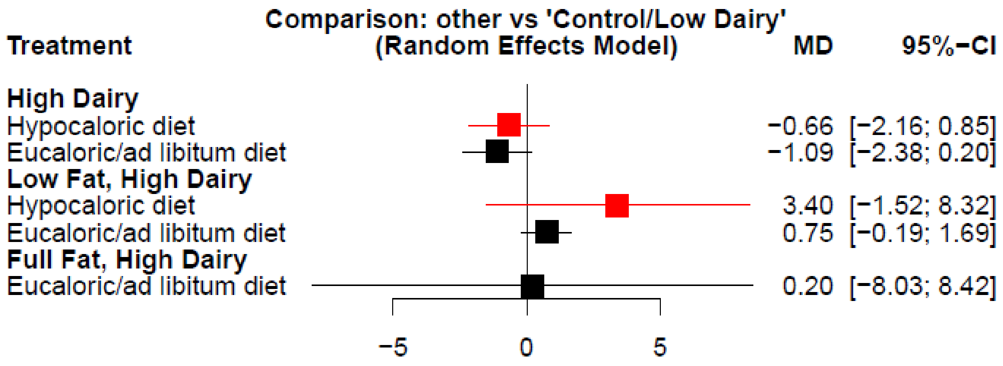

C) Waist Circumference (cm; n studies: 12; n participants: 809)

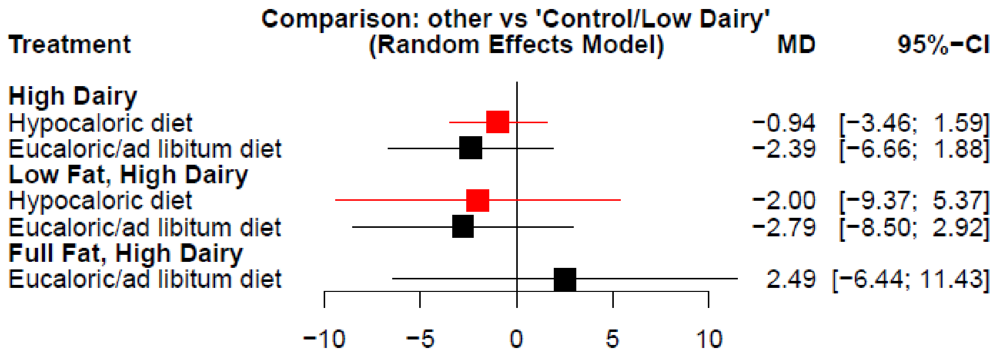

D) LDL-C (mmol/L; n studies: 9; n participants: 693)

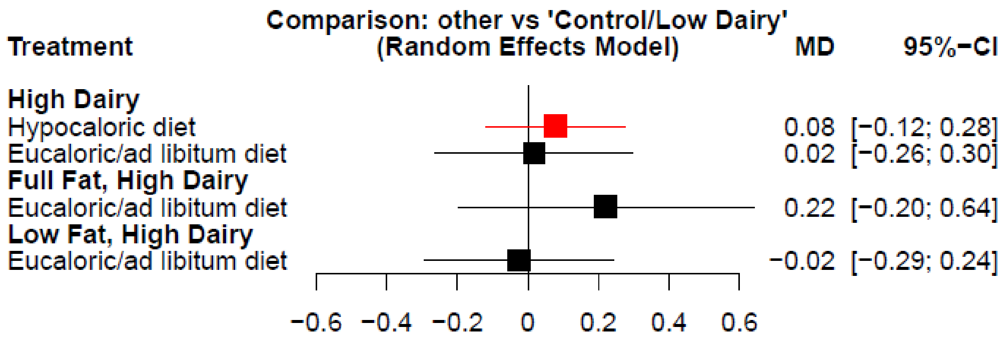

E) HDL-C (mmol/L; n studies: 9; n participants: 693)

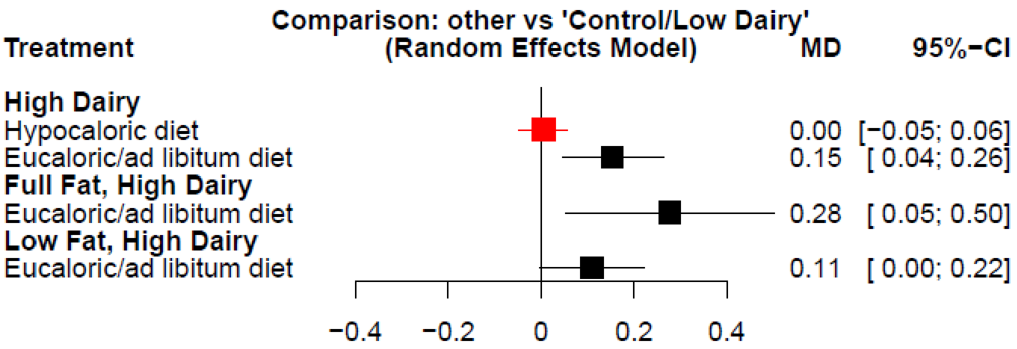

F) Triglycerides (mmol/L; n studies: 9; n participants: 687)

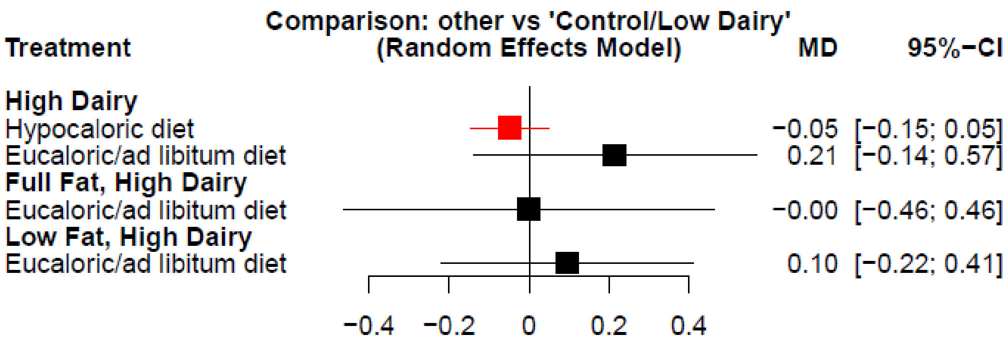

G) Fasting glucose (mmol/L; n studies: 8; n participants: 682)

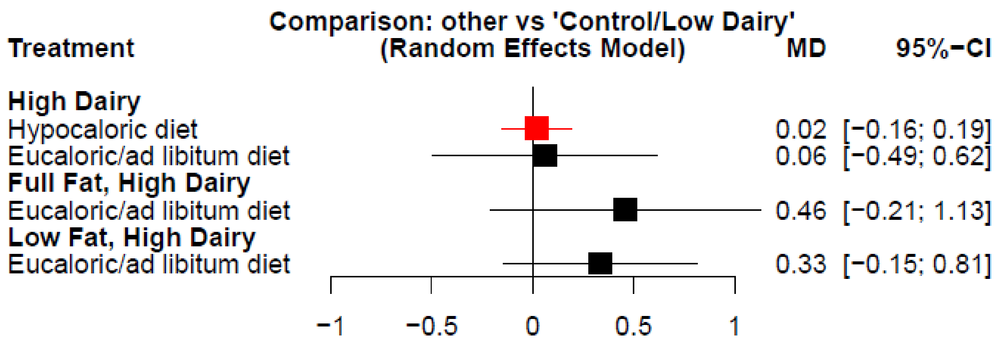

H) Systolic blood pressure (mmHg; n studies: 8; n participants: 665)

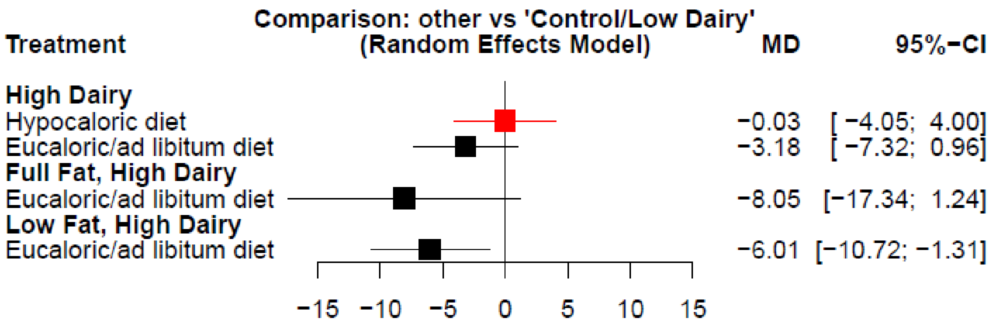

I) Energy intake (kcal/d; n studies:14; n participants: 945)

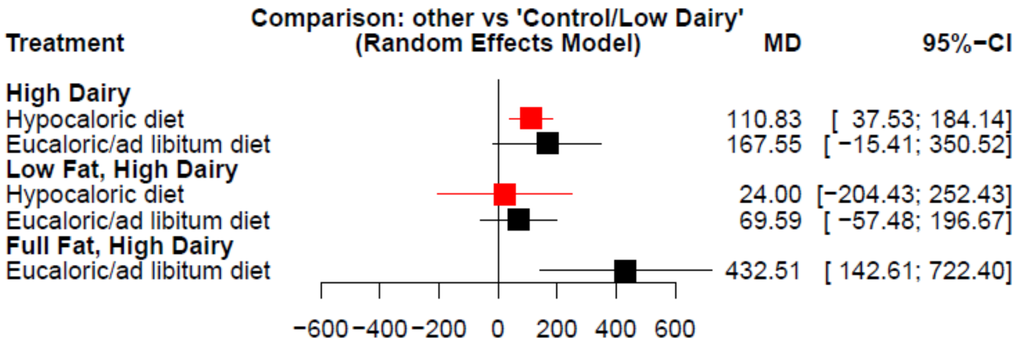

Supplemental Figure 10: Forest plots of subgroup analyses (hypocaloric diet [red] and eucaloric/ad libitum diet [black]) summarizing mean differences with 95% confidence intervals for (A) body weight, (B) fat mass, (C) waist circumference, (D) LDL-C, (E) HDL-C, (F) triglycerides, (G) fasting glucose, (H) systolic blood pressure and (I) energy intake as estimated from the network meta-analysis on total dairy intake with a combined control/low dairy group

Control/low dairy: usual diet or a diet with 0-2 dairy servings/day or an equal amount in grams/day; high dairy: ≥3 dairy servings/day or an equal amount in grams/day; full fat dairy: dairy products with its natural fat content; low fat dairy: skimmed or semi-skimmed dairy products

MD: CI: Confidence Interval; Mean Difference

References of included studies

43. Bellikci-Koyu E, Sarer-Yurekli BP, Akyon Y, Aydin-Kose F, Karagozlu C, Ozgen AG, et al. Effects of Regular Kefir Consumption on Gut Microbiota in Patients with Metabolic Syndrome: A Parallel-Group, Randomized, Controlled Study. Nutrients. 2019;11(9):2089.

44. Bendtsen LQ, Blædel T, Holm JB, Lorenzen JK, Mark AB, Kiilerich P, et al. High intake of dairy during energy restriction does not affect energy balance or the intestinal microflora compared with low dairy intake in overweight individuals in a randomized controlled trial. Applied physiology, nutrition, and metabolism = Physiologie appliquee, nutrition et metabolisme. 2018;43(1):1-10.

45. Campbell CG, Luedecke LO, Shultz TD. Yogurt consumption and estrogen metabolism in healthy premenopausal women. Nutrition Research. 1999;19(4):531-43.

46. Chen Y, Feng RN, Yang X, Dai JX, Huang M, Ji XN, et al. Yogurt improves insulin resistance and liver fat in obese women with nonalcoholic fatty liver disease and metabolic syndrome: a randomized controlled trial. American Journal of Clinical Nutrition. 2019;109(6):1611-9.

47. Engel S, Tholstrup T, Bruun JM, Astrup A, Richelsen B, Raben A. Effect of high milk and sugar-sweetened and non-caloric soft drink intake on insulin sensitivity after 6 months in overweight and obese adults: a randomized controlled trial. European Journal of Clinical Nutrition. 2018;72(3):358-66.

48. Gunther CW, Legowski PA, Lyle RM, McCabe GP, Eagan MS, Peacock M, et al. Dairy products do not lead to alterations in body weight or fat mass in young women in a 1-y intervention. American Journal of Clinical Nutrition. 2005;81(4):751-6.

49. Harvey-Berino J, Gold BC, Lauber R, Starinski A. The impact of calcium and dairy product consumption on weight loss. Obesity Research. 2005;13(10):1720-6.

50. Raziani F, Tholstrup T, Kristensen MD, Svanegaard ML, Ritz C, Astrup A, et al. High intake of regular-fat cheese compared with reduced-fat cheese does not affect LDL cholesterol or risk markers of the metabolic syndrome: a randomized controlled trial. American Journal of Clinical Nutrition. 2016;104(4):973-81.

51. Rideout TC, Marinangeli CP, Martin H, Browne RW, Rempel CB. Consumption of low-fat dairy foods for 6 months improves insulin resistance without adversely affecting lipids or bodyweight in healthy adults: a randomized free-living cross-over study. Nutrition Journal. 2013;12:56.

52. Schmidt KA, Cromer G, Burhans MS, Kuzma JN, Hagman DK, Fernando I, et al. The impact of diets rich in low-fat or full-fat dairy on glucose tolerance and its determinants: a randomized controlled trial. American Journal of Clinical Nutrition. 2021;113(3).

53. Tanaka S, Uenishi K, Ishida H, Takami Y, Hosoi T, Kadowaki T, et al. A Randomized Intervention Trial of 24-wk Dairy Consumption on Waist Circumference, Blood Pressure, and Fasting Blood Sugar and Lipids in Japanese Men with Metabolic Syndrome. Journal of Nutritional Science and Vitaminology. 2014;60(5):305-12.

54. Thomas DT, Wideman L, Lovelady CA. Effects of Calcium and Resistance Exercise on Body Composition in Overweight Premenopausal Women. Journal of the American College of Nutrition. 2010;29(6):604-11.

55. Thompson WG, Holdman NR, Janzow DJ, Slezak JM, Morris KL, Zemel MB. Effect of energy-reduced diets high in dairy products and fiber on weight loss in obese adults. Obesity Research. 2005;13(8):1344-53.

56. Van Loan MD, Keim NL, Adams SH, Souza E, Woodhouse LR, Thomas A, et al. Dairy Foods in a Moderate Energy Restricted Diet Do Not Enhance Central Fat,Weight, and Intra-Abdominal

Adipose Tissue Losses nor Reduce Adipocyte Size or Inflammatory Markers in Overweight and Obese Adults: A Controlled Feeding Study. Journal of Obesity. 2011;2011:989657.

57. Wennersberg MH, Smedman A, Turpeinen AM, Retterstol K, Tengblad S, Lipre E, et al. Dairy products and metabolic effects in overweight men and women: results from a 6-mo intervention study. American Journal of Clinical Nutrition. 2009;90(4):960-8.

58. Zemel MB, Thompson W, Milstead A, Morris K, Campbell P. Calcium and dairy acceleration of weight and fat loss during energy restriction in obese adults. Obesity Research. 2004;12(4):582-90.

59. Zemel MB, Richards J, Milstead A, Campbell P. Effects of calcium and dairy on body composition and weight loss in African-American adults. Obesity Research. 2005;13(7):1218-25.

60. Zemel MB, Teegarden D, Van Loan M, Schoeller DA, Matkovic V, Lyle RM, et al. Dairy-Rich Diets Augment Fat Loss on an Energy-Restricted Diet: A Multicenter Trial. Nutrients. 2009;1(1):83-100.

References of excluded studies

79. Baran D, Sorensen A, Grimes J, Lew R, Karellas A, Johnson B, et al. Dietary modification with dairy products for preventing vertebral bone loss in premenopausal women: a three-year prospective study. Journal of Clinical Endocrinology & Metabolism. 1990;70(1).

80. Rossi F, Alberto B, Jessica C, Simona B. Diets containing dairy foods positively affects weight and fat loss and cytokines blood levels in premenopausal obese women. Mediterranean Journal of Nutrition and Metabolism. 2015;8(2).

81. Nakhoul R, Beeson L, Fakhrawi D, Darnell A, Shulz E, Cordero-MacIntyre Z. Dairy calcium intake and relationship to Bone Mineral Density BMD, Bone Mineral Content BMC and leptin in obese post-menopausal women. FASEB Journal. 2011;25.

82. Banach K, Glibowski P, Jedut P. The Effect of Probiotic Yogurt Containing Lactobacillus Acidophilus LA-5 and Bifidobacterium Lactis BB-12 on Selected Anthropometric Parameters in Obese Individuals on an Energy-Restricted Diet: A Randomized, Controlled Trial. Applied Sciences-Basel. 2020;10(17).

83. Ghizi A, de Almeida Silva M, Moraes F, da Silva CL, Endringer DC, Scherer R, et al. Kefir improves blood parameters and reduces cardiovascular risks in patients with metabolic syndrome. PharmaNutrition. 2021;16.

84. Jones KW, Eller LK, Parnell JA, Doyle-Baker PK, Edwards AL, Reimer RA. Effect of a dairy- and calcium-rich diet on weight loss and appetite during energy restriction in overweight and obese adults: a randomized trial. European Journal of Clinical Nutrition. 2013;67(4).

85. Josse AR, Atkinson SA, Tarnopolsky MA, Phillips SM. Increased Consumption of Dairy Foods and Protein during Diet- and Exercise-Induced Weight Loss Promotes Fat Mass Loss and Lean Mass Gain in Overweight and Obese Premenopausal Women. Journal of Nutrition. 2011;141(9).

86. Rosado JL, Garca OP, Ronquillo D, Hervert D, Rodrguez G, Gutierrez J, et al. The inclusion of milk fortified with micronutrients increases the effectiveness of a calorie restricted diet to reduce obesity in women. The FASEB Journal Conference: Experimental Biology 2009 (EB 2009) New Orleans, LA United States. 2009;23(Suppl 1).

87. Rosado JL, Garcia OP, Ronquillo D, Hervert-Hernandez D, Caamano MDC, Martinez G, et al. Intake of Milk with Added Micronutrients Increases the Effectiveness of an Energy-Restricted Diet to Reduce Body Weight: A Randomized Controlled Clinical Trial in Mexican Women. Journal of the American Dietetic Association. 2011;111(10).

88. Thomson RL, Brinkworth GD, Noakes M, Buckley JD. Muscle strength gains during resistance exercise training are attenuated with soy compared with dairy or usual protein intake in older adults: A randomized controlled trial. Clinical Nutrition. 2016;35(1).

89. Ting GP, Tan SY, Chan SP, Karuthan C, Zaitun Y, Suriah AR, et al. A follow-up study on the effects of a milk supplement on bone mineral density of postmenopausal Chinese women in Malaysia. Journal of Nutrition, Health & Aging. 2007;11(1).

90. Torres MR, Sanjuliani AF. Effects of weight loss from a high-calcium energy-reduced diet on biomarkers of inflammatory stress, fibrinolysis, and endothelial function in obese subjects. Nutrition. 2013;29(1).

91. Hirota T, Kawasaki I, Hirota K. Milk intake accompanied with higher intake of vitamin D might efficiently decrease body fat mass and increase lean mass during moderate weight loss in normal weight young Japanese women. Osteoporosis International. 2010;21.

92. Thorpe MP, Jacobson EH, Layman DK, He XM, Kris-Etherton PM, Evans EM. A diet high in protein, dairy, and calcium attenuates bone loss over twelve months of weight loss and maintenance relative to a conventional high-carbohydrate diet in adults. Journal of Nutrition. 2008;138(6).

93. Bowen J, Noakes M, Clifton PM. A high dairy protein, high-calcium diet minimizes bone turnover in overweight adults during weight loss. Journal of Nutrition. 2004;134(3).

94. Bowen J, Noakes M, Clifton PM. Effect of calcium and dairy foods in high protein, energy-restricted diets on weight loss and metabolic parameters in overweight adults. International Journal of Obesity. 2005;29(8).

95. Crippa G ZDBECFMBERF. Randomized, double-blind, placebo-controlled, cross-over study on the antihypertensive effect of dietary integration with Grana Padano DOCG cheese. Journal of the American Society of Hypertension. 2016;10(4).

96. Parr EB, Coffey VG, Burke LM, Phillips SM, Hawley JA. Effect of increased dairy consumption on weight loss during 16 weeks of energy restriction and exercise training. International Journal of Sport Nutrition and Exercise Metabolism Conference: 2014 International Sports and Exercise Nutrition Conference, ISENC 2014 United Kingdom. 2015;25(Supplement 1).

97. Parr EB, Coffey VG, Cato LE, Phillips SM, Burke LM, Hawley JA. A Randomized Trial of High-Dairy-Protein, Variable-Carbohydrate Diets and Exercise on Body Composition in Adults with Obesity. Obesity. 2016;24(5).

98. Richelsen B, Kristensen K, Pedersen SB. Long-term (6 months) effect of a new fermented milk product on the level of plasma lipoproteins - A placebo-controlled and double blind study. European Journal of Clinical Nutrition. 1996;50(12).

99. Stancliffe RA, Thorpe T, Zemel MB. Dairy attentuates oxidative and inflammatory stress in metabolic syndrome. American Journal of Clinical Nutrition. 2011;94(2).

100. Zemel MB, Richards J, Mathis S, Milstead A, Gebhardt L, Silva E. Dairy augmentation of total and central fat loss in obese subjects. International Journal of Obesity. 2005;29(4).

101. Josse AR, Atkinson SA, Tarnopolsky MA, Phillips SM. Diets Higher in Dairy Foods and Dietary Protein Support Bone Health during Diet-and Exercise-Induced Weight Loss in Overweight and Obese Premenopausal Women. Journal of Clinical Endocrinology & Metabolism. 2012;97(1).

102. Alemán-Mateo H, Carreón VR, Macías L, Astiazaran-García H, Gallegos-Aguilar AC, Enríquez JR. Nutrient-rich dairy proteins improve appendicular skeletal muscle mass and physical performance, and attenuate the loss of muscle strength in older men and women subjects: a single-blind randomized clinical trial. Clinical Interventions In Aging. 2014;9.

103. Aleman-Mateo H, Macias L, Esparza-Romero J, Astiazaran-Garcia H, Blancas AL. Physiological effects beyond the significant gain in muscle mass in sarcopenic elderly men: evidence from a randomized clinical trial using a protein-rich food. Clinical Interventions In Aging. 2012;7:225-34.

104. Fonolla J, Lopez-Huertas E, Machado FJ, Molina D, Alvarez I, Marmol E, et al. Milk enriched with "healthy fatty acids" improves cardiovascular risk markers and nutritional status in human volunteers. Nutrition. 2009;25(4).

105. Loria-Kohen V, Espinosa-Salinas I, de Molina AR, Casas-Agustench P, Herranz J, Molina S, et al. A genetic variant of PPARA modulates cardiovascular risk biomarkers after milk consumption. Nutrition. 2014;30(10).

106. Petrogianni M, Kanellakis S, Moschonis G, Manios Y. Fortification of vitamin A in a phytosterol enriched milk maintains plasma beta-carotene levels. Journal of Food Science & Technology-Mysore. 2014;51(1).

107. Van de Water J, Keen CL, Gershwin ME. The influence of chronic yogurt consumption on immunity. The Journal of nutrition. 1999;129(7 Suppl).

108. Barr SI, McCarron DA, Heaney RP, Dawson-Hughes B, Berga SL, Stern JS, et al. Effects of increased consumption of fluid milk on energy and nutrient intake, body weight, and cardiovascular risk factors in healthy older adults. Journal of the American Dietetic Association. 2000;100(7).

109. Crichton GE, Howe PR, Buckley JD, Coates AM, Murphy KJ, Bryan J. Long-term dietary intervention trials: critical issues and challenges. Trials. 2012;13.

110. Crichton GE, Murphy KJ, Howe PR, Buckley JD, Bryan J. Dairy consumption and working memory performance in overweight and obese adults. Appetite. 2012;59(1).

111. Crichton Ge HPRCBJDCAMMKJ. Dairy consumption and cardiometabolic health: Outcomes of a 12-month crossover trial. Nutrition and metabolism. 2012;9.

112. Smilowitz JT, Wiest MM, Teegarden D, Zemel MB, German JB, Van Loan MD. Dietary fat and not calcium supplementation or dairy product consumption is associated with changes in anthropometrics during a randomized, placebo-controlled energy-restriction trial. Nutrition & Metabolism. 2011;8.

113. Zemel MB, Donnelly JE, Smith BK, Sullivan DK, Richards J, Morgan-Hanusa D, et al. Effects of dairy intake on weight maintenance. Nutrition & Metabolism. 2008;5.

114. Hinton PS, Rector RS, Donnelly JE, Smith BK, Bailey B. Total body bone mineral content and density during weight loss and maintenance on a low- or recommended-dairy weight-maintenance diet in obese men and women. European Journal of Clinical Nutrition. 2010;64(4).

115. Bailey BW, Smith BK, Sullivan DK, Donnelly JE. The effects of dairy consumption on metabolic risk factors for ischemic heart disease. Obesity (Silver Spring, Md). 2011;19.

116. Bajahzer MF, Bruun JM, Rosqvist F, Marklund M, Richelsen B, Risérus U. Effects of sugar-sweetened soda on plasma saturated and monounsaturated fatty acids in individuals with obesity: A randomized study. Front Nutr. 2022;9:936828.

117. Bowen J, Noakes M, Clifton P. High dairy-protein versus high mixed-protein energy restricted diets - the effect on bone turnover and calcium excretion in overweight adults. Asia Pacific Journal of Clinical Nutrition. 2003;12 Suppl.

118. Eagan MS, Lyle RM, Gunther CW, Peacock M, Teegarden D. Effect of 1-year dairy product intervention on fat mass in young women: 6-month follow-up. Obesity. 2006;14(12).

119. Hauge Wennersberg M, Smedman A, Turpeinen A, Retterstl K, Tengblad S, Lipre E, et al. Addition of milk products in the diet of overweight subjects has small effects on metabolic variables. Journal Of Diabetes. 2009;1.

120. Josse AR, Atkinson SA, Tarnopolsky MA, Phillips SM. Consumption of higher dairy and dietary protein during diet- and exercise-induced weight loss promotes a metabolically favourable body composition change in overweight and obese young women. FASEB Journal. 2011;25.

121. Piccolo BD, Keim NL, Fiehn O, Adams SH, Van Loan MD, Newman JW. Habitual Physical Activity and Plasma Metabolomic Patterns Distinguish Individuals with Low vs. High Weight Loss during Controlled Energy Restriction. Journal of Nutrition. 2015;145(4).

122. Rideout T, Marinangeli C, Rempel C. Consumption of low-fat dairy foods for 6 months improves insulin resistance without adversely affecting lipids or bodyweight in healthy adults. FASEB Journal. 2014;28(1 SUPPL. 1).

123. Woodhouse LR, Peerson J, Souza E, Domek J, Freytag T, Padda G, et al. Effects of dairy foods in a moderate energy restricted diet on inflammatory markers and plasma zinc in overweight and obese adults. FASEB Journal. 2011;25.

124. Zemel MB, Thompson W, Zemel P, Nocton AM, Milstead A, Morris K, et al. Dietary calcium and dairy products accelerate weight and fat loss during energy restriction in obese adults. American Journal of Clinical Nutrition. 2002;75(2).

125. Shlisky JD, Durward CM, Zack MK, Campbell JK, Jonnalagadda SS, Gugger C, et al. Effects of an energy-restricted, moderate-protein diet plan with non-fat dairy on weight changes: 12 weeks of weight loss (WL) followed by 12 weeks of WL maintenance. FASEB Journal. 2012;26.

126. Shlisky JD, Durward CM, Zack MK, Gugger CK, Campbell JK, Nickols-Richardson SM. An energy-reduced dietary pattern, including moderate protein and increased nonfat dairy intake combined with walking promotes beneficial body composition and metabolic changes in women with excess adiposity: a randomized comparative trial. Food Science & Nutrition. 2015;3(5).
